# *Ppp2r1a* haploinsufficiency increases excitatory synaptic transmission and decreases spatial learning by impairing endocannabinoid signaling

**DOI:** 10.1172/JCI185602

**Published:** 2025-08-21

**Authors:** Yirong Wang, Weicheng Duan, Hua Li, Zhiwei Tang, Ruyi Cai, Shangxuan Cai, Guanghao Deng, Liangpei Chen, Hongyan Luo, Liping Chen, Yulong Li, Jian-Zhi Wang, Bo Xiong, Man Jiang

**Affiliations:** 1 Department of Physiology, School of Basic Medicine and Tongji Medical College,; 2 Institute for Brain Research, Collaborative Innovation Center for Brain Science, and; 3 Department of Forensic Medicine, Tongji Medical College, Huazhong University of Science and Technology, Wuhan, China.; 4 State Key Laboratory of Membrane Biology, Peking University School of Life Sciences, Beijing, China.; 5 Peking University IDG/McGovern Institute for Brain Research, Beijing, China.; 6 Guangdong Provincial Key Laboratory of Food, Nutrition and Health, Department of Toxicology, School of Public Health, Sun Yat-sen University, Guangzhou, China.; 7 Peking-Tsinghua Center for Life Sciences, Academy for Advanced Interdisciplinary Studies, Peking University, Beijing, China.; 8 Chinese Institute for Brain Research, Beijing, China.; 9 Department of Pathophysiology, Key Laboratory of Ministry of Education for Neurological Disorders, School of Basic Medicine and Tongji Medical College,; 10 Tongji-Rongcheng Center for Biomedicine, and; 11 Hubei Key Laboratory of Drug Target Research and Pharmacodynamic Evaluation, Huazhong University of Science and Technology, Wuhan, China.

**Keywords:** Development, Neuroscience, Intellectual disability, Neurodevelopment, Synapses

## Abstract

Protein phosphatase 2A (PP2A) is a serine/threonine phosphatase in the brain. Mutations in *PPP2R1A*, encoding the scaffolding subunit, are linked to intellectual disability, although the underlying mechanisms remain unclear. This study examined mice with heterozygous deletion of *Ppp2r1a* in forebrain excitatory neurons (NEX-het-conditional knockout [NEX-het-cKO]). These mice exhibited impaired spatial learning and memory, resembling *Ppp2r1a*-associated intellectual disability. *Ppp2r1a* haploinsufficiency also led to increased excitatory synaptic strength and reduced inhibitory synapse numbers on pyramidal neurons. The increased excitatory synaptic transmission was attributed to increased presynaptic release probability, likely due to reduced levels of 2-arachidonoyl glycerol (2-AG). This reduction in 2-AG was associated with increased transcription of monoacylglycerol lipase (MAGL), driven by destabilization of enhancer of zeste homolog 2 (EZH2) in NEX-het-cKO mice. Importantly, the MAGL inhibitor JZL184 effectively restored both synaptic and learning deficits. Our findings uncover an unexpected role of PPP2R1A in regulating endocannabinoid signaling, providing fresh molecular and synaptic insights into the mechanisms underlying intellectual disability.

## Abstract 2

Loss of *Ppp2r1a* impairs brain signaling and memory in mice, but blocking endocannabinoid degradation with MAGL inhibition effectively restores these deficits.

## Graphical abstract

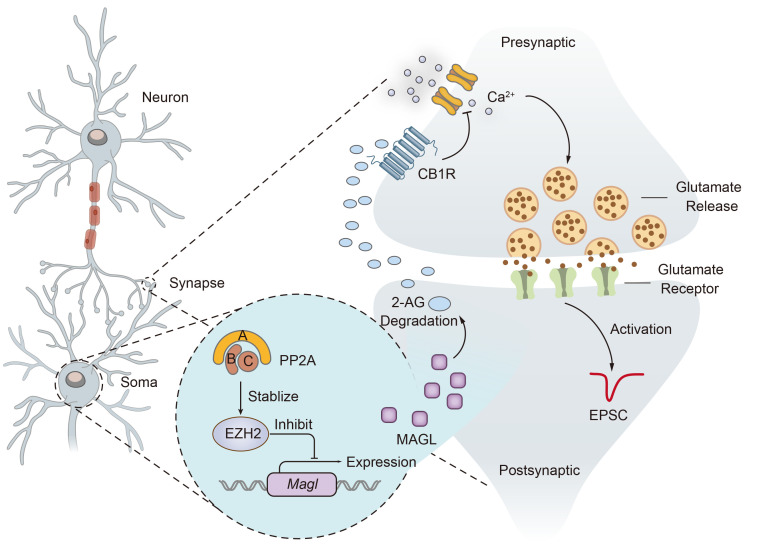

## Introduction

Neurodevelopmental disorders (NDDs) include autism spectrum disorder (ASD), intellectual disability, attention-deficit hyperactivity disorder, abnormal anxiety, epilepsy, and developmental delay (1, 2). Clinical studies have linked various candidate gene variants to NDDs, which result from multidimensional neurodevelopmental abnormalities, often due to issues with transcriptional, translational, and synaptic functions (1, 3). Among NDD risk factors, protein phosphatase 2A (PP2A) — a highly abundant serine/threonine phosphatase in the brain (4) — has emerged as a particularly compelling candidate (5–9). This heterotrimeric enzyme consists of scaffolding (PP2A-A), regulatory (PP2A-B), and catalytic subunits (PP2A-C) (10, 11). Traditionally, PP2A is best known for its dephosphorylation activity (12, 13). In vitro studies have identified over 300 potential substrates, including tau, GSK-3β, Akt, CaMKII, CRMP2, Dock6, and ERK (14–18). Recent evidence suggests that PP2A may also interact with RNA polymerase II (Pol II), regulating gene transcription through Pol II dephosphorylation (19). Clinically, pathogenic variants in *PPP2R1A* (encoding PP2A-A) and *PPP2R5D* (encoding PP2A-B) have been linked to NDDs characterized by severe intellectual disability, speech impairment, epilepsy, and developmental delay (5–9). These *PPP2R1A* mutations are proposed to disrupt holoenzyme assembly (5). However, the molecular and cellular mechanisms by which *PPP2R1A* deficiency contributes to abnormal brain function remain unclear.

The endocannabinoid (eCB) system — comprising endogenous ligands, receptors, and the enzymes that govern their synthesis and degradation — is widely expressed throughout the vertebrate nervous system (20). The 2 main receptors, cannabinoid type 1 and 2 receptors (CB1Rs and CB2Rs) (20), are activated by eCBs such as 2-arachidonoyl glycerol (2-AG) and anandamide (AEA) (21). As retrograde trans-synaptic messengers, eCBs typically suppress neurotransmitter release by activating presynaptic CB1Rs (22). Clinical studies have reported reduced eCB levels in individuals with ASD (23, 24), and genetic variants in diacylglycerol lipase α (DAGLα, 2-AG synthase) have been associated with NDDs, including ASD and abnormal brain development (25). Furthermore, recent clinical trials suggested that cannabidiol-rich cannabis treatment can improve behavioral performance in some individuals with ASD, indicating a potential link between eCB deficiency and neuropsychiatric disorders (26). However, it remains unclear how eCB signaling contributes to abnormal brain function in these disorders.

In our study, we asked whether *Ppp2r1a* dysfunction contributes to intellectual disability. Our data show that heterozygous deletion of *Ppp2r1a* in forebrain excitatory neurons (NEX-Cre; *Ppp2r1a*^f/+^, NEX-het-conditional knockout [NEX-het-cKO]) in mice impaired spatial learning and memory, mimicking the intellectual disability phenotype associated with human *PPP2R1A* variants. Electrophysiological recordings revealed enhanced excitatory synaptic strength in the medial prefrontal cortex (mPFC) of NEX-het-cKO mice due to an increased release probability (Pr). Further RNA sequencing experiments revealed that *Ppp2r1a* haploinsufficiency may upregulate the transcription of monoacylglycerol lipase (*Magl*), a key hydrolase responsible for 2-AG degradation, in an enhancer of zeste homolog 2 (EZH2)–dependent manner. Consistently, fiber photometry recordings showed a significant reduction in 2-AG release in NEX-het-cKO mice. Notably, treatment with the MAGL inhibitor JZL184 restored the excitatory synaptic Pr and ameliorated the spatial learning and memory deficits. These findings uncover an unexpected role of *Ppp2r1a* in regulating eCB signaling and provide compelling insights into synaptic dysfunction linked to *PPP2R1A*-associated NDDs.

## Results

### Ppp2r1a haploinsufficiency in forebrain excitatory neurons reduces anxiety level and impairs spatial learning.

Given the importance of *PPP2R1A* in NDDs, we first investigated its spatiotemporal expression patterns in vivo. At postnatal day 45 (P45), PPP2R1A expression was high in the brain and spinal cord, but relatively low in peripheral organs such as the heart, lung, liver, spleen, and kidney (Supplemental Figure 1A; supplemental material available online with this article; https://doi.org/10.1172/JCI185602DS1). Further analysis showed widespread PPP2R1A expression across the brain, with the highest levels in the cortex and hippocampus (Supplemental Figure 1B). Temporal profiling showed that PPP2R1A levels gradually increased after birth, plateauing in early adulthood (Supplemental Figure 1C). Reanalyzing single-nucleus RNA-Seq (snRNA-Seq) datasets (27, 28) demonstrated widespread *Ppp2r1a* expression, with notably high expression levels in glutamatergic neurons (Supplemental Figure 1, D and E). Given that glutamatergic neurons constitute approximately 80% of forebrain neurons (29) and play a crucial role in synaptic plasticity related to learning and memory (30), we focused subsequent studies on investigating the function of *Ppp2r1a* in forebrain excitatory neurons.

**Figure 1:**
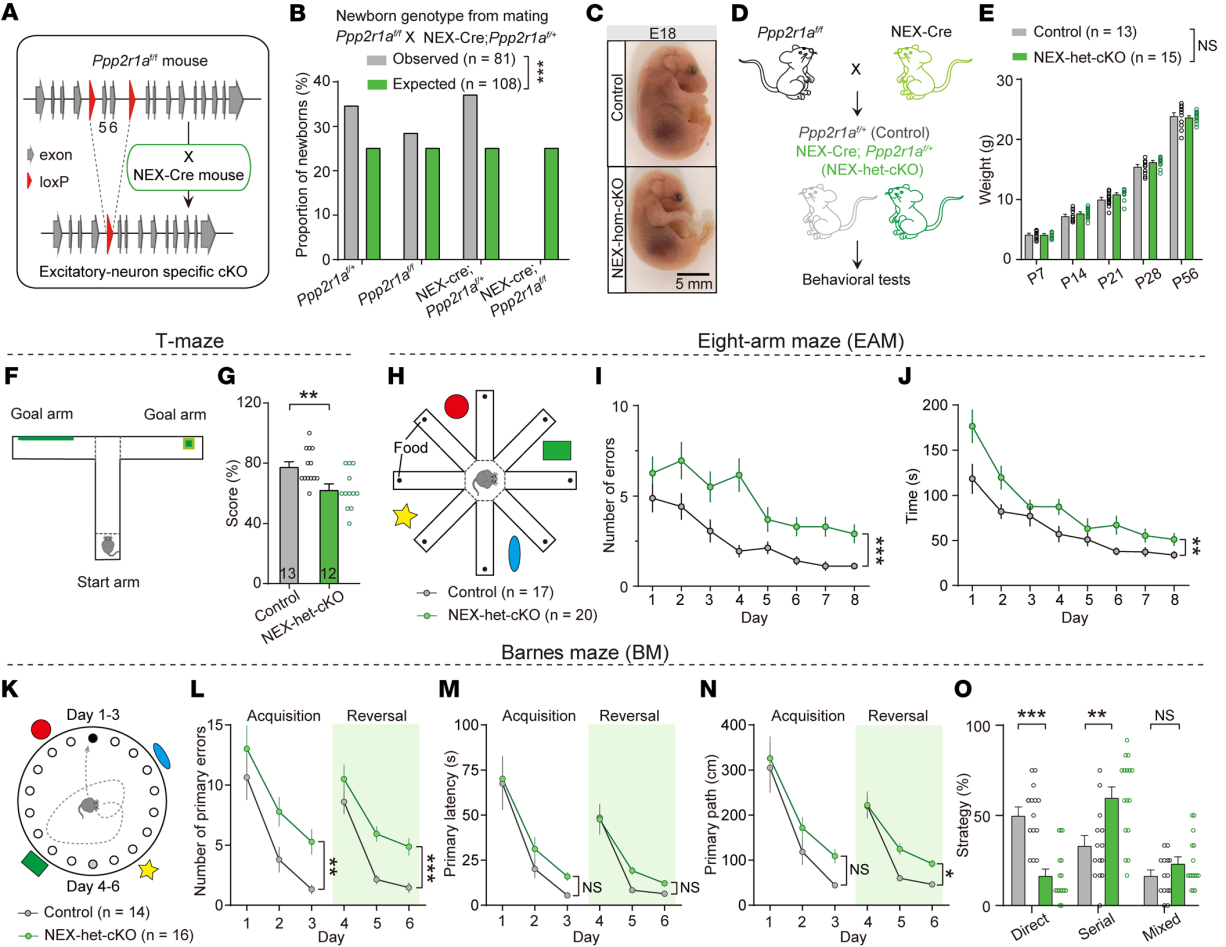
*Ppp2r1a* haploinsufficiency in forebrain excitatory neurons impairs spatial learning and memory. (**A**) Schematic representation of forebrain excitatory neuron–specific *Ppp2r1a* cKO in mice. (**B**) Genotype analysis of the offspring showing that homozygous *Ppp2r1a* deletion is lethal (observed, *n* = 81 mice; expected, *n* = 108 mice). (**C**) NEX-Cre; *Ppp2r1a*^fl/fl^ (NEX-hom-cKO) pups displaying dysplasia at E18. Scale bar: 5 mm. (**D**) Schematic illustrating that littermate control and NEX-het-cKO mice are used in all experiments. (**E**) Body weights are normal in NEX-het-cKO mice (Control, *n* = 13; NEX-het-cKO, *n* = 15). (**F** and **G**) NEX-het-cKO mice exhibited impaired performance in the T-maze (Control, *n* = 13; NEX-het-cKO, *n* = 12). (**H**–**J**) NEX-het-cKO mice exhibited impaired performance in the EAM (Control, *n* = 17; NEX-het-cKO, *n* = 20). (**H**) Schematic of the EAM. (**I**) Number of errors. (**J**) Time spent seeking out all pellets. (**K**–**O**) NEX-het-cKO mice demonstrated impaired performance in the BM (Control, *n* = 14; NEX-het-cKO, *n* = 16). (**K**) Schematic of the BM. (**L**) Number of primary errors. (**M**) Primary latency. (**N**) Primary path length. (**O**) Percentage of strategy used. Statistical comparisons were performed using 2-tailed unpaired Student’s *t* test (**G** and **O**), Chi-square test (**B**), and 2-way ANOVA followed by Bonferroni’s post hoc test (**E**, **I**, **J**, and **L–N**). All data are presented as mean ± SEM. *P < 0.05, **P < 0.01, ***P < 0.001.

We first crossed NEX-Cre mice with LSL-H2B-GFP reporter mice (Supplemental Figure 2A) and confirmed the high specificity of NEX-Cre mice for labeling forebrain excitatory neurons (31). We next crossed *Ppp2r1a*^fl/fl^ mice with NEX-Cre mice to achieve selective deletion of *Ppp2r1a* in these neurons starting from the embryonic stage (Figure 1A). Homozygous deletion (NEX-Cre; *Ppp2r1a*^fl/fl^, NEX-hom-cKO) resulted in perinatal lethality (Figure 1B), together with developmental abnormalities and dysplasia detectable at embryonic day 18 (E18) (Figure 1C). Clinical studies showed that heterozygous loss-of-function mutations in *PPP2R1A* (e.g., P179L, R182W, S219L, and R258H) are linked to intellectual disability (5, 9), suggesting that *PPP2R1A* haploinsufficiency may be a significant risk factor. To test this hypothesis, all subsequent experiments were performed using NEX-Cre; *Ppp2r1a*^f/+^ (NEX-het-cKO) and their littermate controls (*Ppp2r1a*^f/+^) (Figure 1D).

Compared with their littermate controls, the NEX-het-cKO mice had normal body weight (Figure 1E), brain weight, and brain size (Supplemental Figure 2, B–D). Behavioral assessment revealed no significant differences between NEX-het-cKO and control mice in the open field test, rearing behavior, marble burying test, grip strength measurements, 3-chamber social test, novel object recognition test, or tube test, indicating intact motor function, repetitive behavior, muscular strength, baseline sociability, social novelty preference, object recognition, and social dominance (Supplemental Figure 3, A–E and R–T, and Supplemental Figure 4). However, NEX-het-cKO mice made more entries into open arms of the elevated plus maze without a significant change in the percentage of time spent there (Supplemental Figure 3, F–H). Together with reduced grooming time (Supplemental Figure 3, I and J), these findings suggest decreased anxiety level. Additionally, gait analysis revealed a subtle deficit in motor coordination in NEX-het-cKO mice, as reflected by increased overlap irregularity (Supplemental Figure 3, K–Q).

To evaluate learning and memory abilities, key deficits in NDD patients with PPP2R1A mutations (reported in 97%, 29/30 cases) (8), we conducted several behavioral assays. NEX-het-cKO mice exhibited significant impairment in spatial working memory, as demonstrated by reduced T-maze performance (Figure 1, F and G). In the eight-arm maze (EAM), they made more errors and spent more time retrieving baits compared with controls (Figure 1, H–J). In the Barnes maze (BM), NEX-het-cKO mice made more errors, spent more time, and traveled longer path lengths to locate the correct escape hole during both the initial learning phase (days 1–3) and the reversal phase (days 4–6), when the escape box was relocated to the opposite hole (Figure 1, K–N). Strategy analysis revealed that control mice typically used a direct approach (going directly to the target hole), whereas NEX-het-cKO mice predominantly employed a serial approach (exploring the holes 1 by 1) (Figure 1O), further indicating deficits in spatial learning and memory.

Taken together, our results suggest that *Ppp2r1a* haploinsufficiency in forebrain excitatory neurons reduces anxiety levels and severely impairs spatial learning and memory, closely resembling the core symptoms of intellectual disability observed in patients harboring *PPP2R1A* mutations.

### Ppp2r1a haploinsufficiency increases excitatory synaptic transmission and decreases inhibitory synaptic transmission in layer 5 pyramidal neurons of the mPFC.

**Figure 2:**
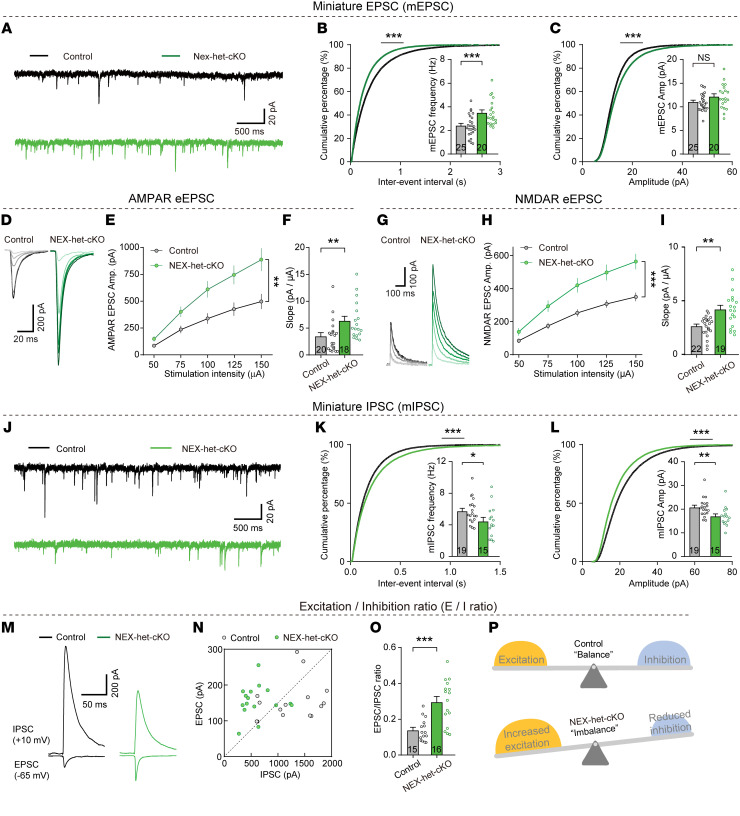
*Ppp2r1a* haploinsufficiency increases excitatory synaptic transmission, decreases inhibitory synaptic transmission, and alters excitation-inhibition balance. (**A–C**) *Ppp2r1a* haploinsufficiency increased mEPSC frequency (Control, *n* = 25 neurons; NEX-het-cKO, *n* = 20 neurons). (**A**) Representative mEPSC traces in mPFC L5 pyramidal neurons. (**B**) Cumulative distribution of interevent interval and summary of mEPSC frequency (inset). (**C**) Cumulative distribution and summary of mEPSC amplitude (inset). (**D–F**) *Ppp2r1a* haploinsufficiency increased AMPAR-mediated synaptic responses (Control, *n* = 20 neurons; NEX-het-cKO, *n* = 18 neurons). (**D**) Representative traces of evoked AMPAR-EPSCs. (**E**) Input/output plot of EPSC amplitude versus stimulation intensity. (**F**) Slope of input/output relationship. (**G**–**I**) *Ppp2r1a* haploinsufficiency increased NMDAR-mediated synaptic responses (Control, *n* = 22 neurons; NEX-het-cKO, *n* = 19 neurons). (**G**) Representative traces of evoked NMDAR-EPSCs. (**H**) Input/output plot of EPSC amplitude versus stimulation intensity. (**I**) Slope of input/output relationship. (**J**–**L**) *Ppp2r1a* haploinsufficiency decreased both the frequency and amplitude of mIPSCs (Control, *n* = 19 neurons; NEX-het-cKO, *n* = 15 neurons). (**J**) Representative mIPSC traces recorded in L5 pyramidal neurons. (**K**) Cumulative distribution of interevent interval and summary of mIPSC frequency (inset). (**L**) Cumulative distribution of mIPSC amplitude and summary graphs of mIPSC amplitude (inset). (**M**–**P**) *Ppp2r1a* haploinsufficiency increased E/I ratio (Control, *n* = 15 neurons; NEX-het-cKO, *n* = 16 neurons). (**M**) Representative traces of EPSCs and IPSCs. (**N**) Scatterplots depicting amplitudes of EPSCs and IPSCs recorded from both genotypes. (**O**) Increased E/I ratio in NEX-het-cKO mice. (**P**) Model illustrating that *Ppp2r1a* haploinsufficiency alters E/I balance. Statistical comparisons were performed using 2-tailed unpaired Student’s *t* test (**C**, **K**, and **L**), 2-tailed unpaired Student’s *t* test with Welch’s correction (**I** and **O**), 2-tailed Mann-Whitney test (**B** and **F**), 2-way ANOVA followed by Bonferroni’s post hoc test (**E** and **H**), and 2-tailed Kolmogorov-Smirnov test (cumulative distribution) (**B**, **C**, **K**, and **L**). All data are presented as mean ± SEM. *P < 0.05, **P < 0.01, ***P < 0.001.

Previous studies have linked impaired synaptic transmission in the mPFC to cognitive dysfunction, including spatial learning deficits (32, 33). We postulated that the spatial learning and memory impairments observed in NEX-het-cKO mice might be due to abnormalities in synaptic function in the mPFC. To investigate the role of PPP2R1A on excitatory synaptic transmission, we first recorded miniature excitatory postsynaptic currents (mEPSCs) in layer 5 (L5) pyramidal neurons of the mPFC at P35–P45. *Ppp2r1a* haploinsufficiency in forebrain excitatory neurons increased mEPSC frequency with a minimal effect on amplitude (Figure 2, A–C). Next, we measured evoked EPSCs mediated by α-amino-3-hydroxy-5-methyl-4-isoxazolepropionic acid receptors (AMPARs) (Figure 2, D–F) and *N*-methyl-d-aspartate receptors (NMDARs) (Figure 2, G–I) and observed a large increase in both in L5 pyramidal neurons from NEX-het-cKO mice compared with controls (Figure 2, D–I). These findings suggest that *Ppp2r1a* haploinsufficiency increases excitatory synaptic transmission in L5 pyramidal neurons of the mPFC.

Next, we recorded miniature inhibitory postsynaptic currents (mIPSCs) in L5 pyramidal neurons of the mPFC. Both the frequency and amplitude of mIPSCs were reduced in NEX-het-cKO mice compared with their littermate controls (Figure 2, J–L), suggesting reduced GABAergic transmission. Supporting this, immunostaining for vesicular GABA transporter (vGAT) — a selective marker for GABAergic synapses (34) — revealed a significant decrease in the density of vGAT^+^ puncta and a slight reduction in their size (Supplemental Figure 5). These findings suggest that *Ppp2r1a* haploinsufficiency reduces the number of inhibitory synapses in the mPFC.

Considering the established role of excitation-inhibition (E/I) imbalance in neuropsychiatric diseases (35), we next asked whether *Ppp2r1a* haploinsufficiency alters this balance. We first determined the reversal potentials of EPSCs and IPSCs (Supplemental Figure 6) and then isolated EPSCs and IPSCs by voltage-clamping neurons at the reversal potentials for IPSCs and EPSCs, respectively (Figure 2, M and N). Compared with controls, L5 pyramidal neurons in NEX-het-cKO mice exhibited a higher E/I ratio (Figure 2, O and P), likely due to both increased excitatory input and decreased inhibitory input. These findings suggest that *Ppp2r1a* haploinsufficiency disrupts synaptic E/I balance in the mPFC.

### Ppp2r1a haploinsufficiency increases presynaptic Pr of excitatory synapses.

**Figure 3:**
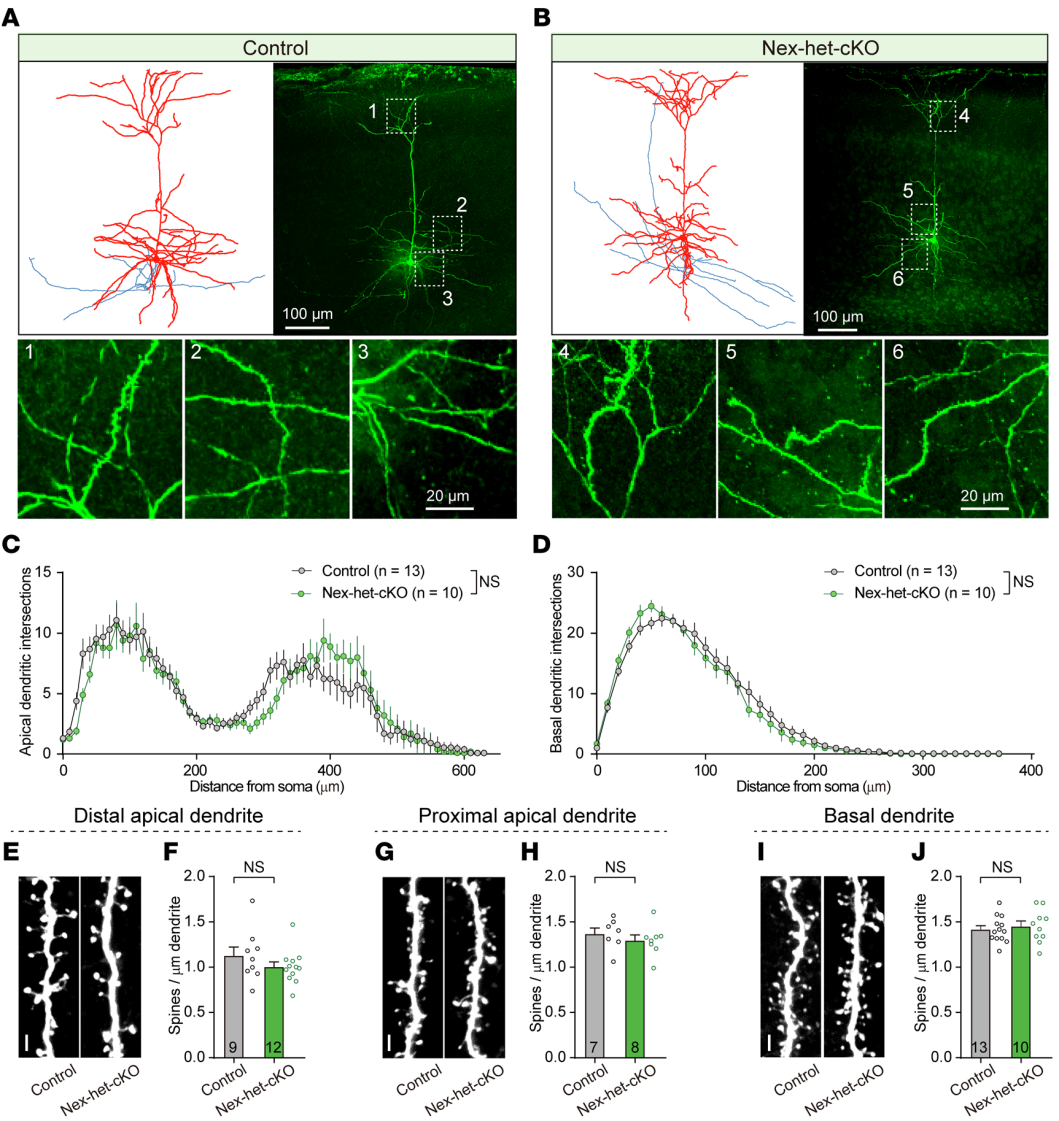
*Ppp2r1a* haploinsufficiency does not alter neuronal morphology. (**A–D**) Normal neuronal morphology in NEX-het-cKO mice (Control, *n* = 13 neurons; NEX-het-cKO, *n* = 10 neurons). (**A** and **B**) Morphological reconstructions of L5 pyramidal neurons from control (**A**) and NEX-het-cKO (**B**) mice. Left, representative reconstruction of biocytin-labeled neurons (red, soma and dendrites; blue, axons). Right, maximum intensity projections of biocytin-labeled neurons. Bottom, magnified area showing dendritic structures (1 and 4, distant apical dendrites; 2 and 5, proximal apical dendrites; 3 and 6, basal dendrites). Scale bars: 100 μm (top), 20 μm (bottom). (**C** and **D**) Group data for Sholl analysis showing normal apical (**C**) and basal (**D**) dendrites in NEX-het-cKO mice. (**E**–**J**) Normal spine density in NEX-het-cKO mice. Scale bar: 2 μm. Representative images (**E**) and summary graph (**F**) illustrating normal spine density in NEX-het-cKO mice (Control, *n* = 9 neurons from 6 mice; NEX-het-cKO, *n* = 12 neurons from 8 mice). (**G** and **H**) Same as **E** and **F** but for proximal apical dendrites (Control, *n* = 7 neurons from 6 mice; NEX-het-cKO, *n* = 8 neurons from 6 mice). (**I** and **J**) Same as **E** and **F** but for basal dendrites (Control, *n* = 13 neurons from 7 mice; NEX-het-cKO, *n* = 10 neurons from 8 mice). To assess spine density along distal apical, proximal apical, and basal dendrites, 3 dendritic segments were analyzed per neuron, and the average spine density was used for subsequent analyses. Statistical comparisons were performed using 2-tailed unpaired Student’s *t* test (**F**, **H**, and **J**) and 2-way ANOVA with Geisser-Greenhouse correction (**C** and **D**). All data are presented as mean ± SEM.

The elevated mEPSC frequency suggested an increase in excitatory synapse number or enhanced presynaptic Pr. To distinguish between these 2 possibilities, we first examined the dendritic morphology of L5 pyramidal neurons in the mPFC using biocytin-avidin labeling (Figure 3, A and B, and Supplemental Figure 7). Sholl analysis of both apical and basal dendrites revealed comparable dendritic complexity between control and NEX-het-cKO mice (Figure 3, C and D). Spine density quantification across dendritic regions also revealed no significant differences (Figure 3, E–J). Thus, *Ppp2r1a* haploinsufficiency did not significantly affect the morphology of L5 pyramidal neurons.

**Figure 4:**
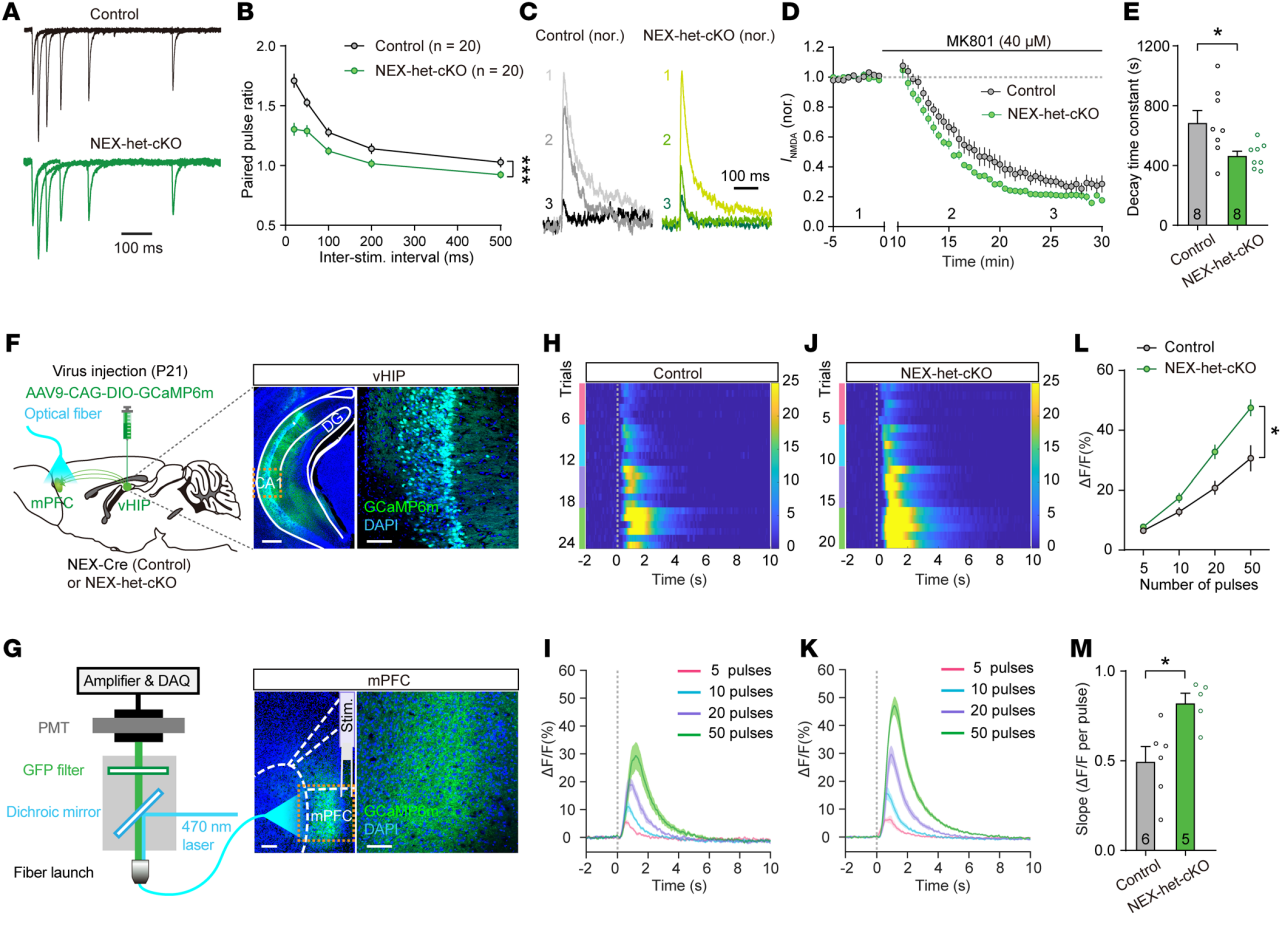
*Ppp2r1a* haploinsufficiency increases presynaptic Pr in excitatory synapses. (**A** and **B**) *Ppp2r1a* haploinsufficiency increased presynaptic Pr (Control, *n* = 20 neurons; NEX-het-cKO, *n* = 20 neurons). (**A**) Representative traces of the PPR of EPSCs. (**B**) Summary graph showing decreased PPR in NEX-het-cKO mice. (**C**) Representative traces of NMDAR-EPSCs recorded before and after perfusion with 40 μM MK801 (at 15 and 25 min). (**D**) Summary showing accelerated blockade of NMDAR-EPSCs in NEX-het-cKO mice. (**E**) Decreased decay time constant of MK801 blockade in NEX-het-cKO mice (Control, *n* = 8 neurons; NEX-het-cKO, *n* = 8 neurons). (**F**–**M**) *Ppp2r1a* haploinsufficiency increased the amplitude of presynaptic Ca^2+^ transients (Control, *n* = 6 mice; NEX-het-cKO, *n* = 5 mice). (**F**) Left, schematic of AAV-CAG-DIO-GCaMP6m injection in the ventral CA1(vCA1) of NEX-Cre (Control) or NEX-hetcKO mice and optical fiber recordings in mPFC slices. Right, representative coronal section showing GCaMP6m-expressing pyramidal neurons in the vCA1. Scale bars: 500 μm (left), 100 μm (right). DG: dentate gyrus. (**G**) Schematic of fiber photometry recordings in mPFC slices. Right, representative coronal section showing GCaMP6m-expressing axon terminals in mPFC. Scale bars: 200 μm (left), 100 μm (right). DAQ, data acquisition; PMT, photomultiplier tube. (**H** and **I**) Train stimulation with an increasing number of pulses (5, 10, 20, and 50) evoked GCaMP signals in control mice. (**H**) Heatmap of aligned GCaMP signals from individual mice, with number of pulses indicated by color. (**I**) Average traces of GCaMP signals across all animals from the control group. (**J** and **K**) Same as **H** and **I** but for NEX-het-cKO mice. (**L**) Input/output plot of GCaMP signals versus number of pulses. (**M**) Slope of input/output relationship. Statistical comparisons were performed using 2-tailed unpaired Student’s *t* test (**M**), 2-tailed unpaired Student’s *t* test with Welch’s correction (**E**), and 2-way ANOVA followed by Bonferroni’s post hoc test (**B** and **L**). All data are presented as mean ± SEM. *P < 0.05, ***P < 0.001.

To test whether *Ppp2r1a* haploinsufficiency affects presynaptic Pr, we recorded EPSC paired-pulse ratio (PPR), a commonly used inverse indicator of presynaptic Pr (36). The results showed a significant decrease in EPSC PPRs in L5 pyramidal neurons from NEX-het-cKO mice (Figure 4, A and B), suggesting enhanced presynaptic Pr at excitatory synapses. To further test this, we examined the rate of use-dependent blockade of pharmacologically isolated NMDAR-EPSCs by MK801, a measure directly correlated with presynaptic Pr (37). The blockade occurred significantly faster in NEX-het-cKO mice than in controls (Figure 4, C–E), confirming an increase in presynaptic Pr at excitatory synapses. To exclude postsynaptic alterations, we assessed synaptosomal protein levels of AMPAR subunits (GluA1 and GluA2), NMDAR subunits (GluN1, GluN2A, and GluN2B), and PSD95, but found no significant differences between groups (Supplemental Figure 8).

Presynaptic calcium (Ca^2+^) transient is a critical determinant of presynaptic Pr (36). To investigate whether the elevated presynaptic Pr observed in NEX-het-cKO mice stems from altered Ca^2+^ dynamics, we recorded axonal Ca^2+^ transients in the vCA1→mPFC glutamatergic pathway, a well-characterized hippocampal-prefrontal projection (38). We injected AAV-CAG-DIO-GCaMP6m into the vCA1 of NEX-Cre (control) and NEX-het-cKO mice and recorded GCaMP signals from vCA1→mPFC axon terminals using fiber photometry in mPFC brain slices (Figure 4, F and G). With comparable GCaMP6m expression in the vCA1 region of both genotypes (Supplemental Figure 9), we delivered 50-Hz electrical stimuli with varying pulse numbers and found a linear increase in GCaMP signal amplitude (Figure 4, H–L). Notably, axonal terminals in NEX-het-cKO mice exhibited significantly larger Ca^2+^ transients than controls (Figure 4, L and M). These results suggest that *Ppp2r1a* haploinsufficiency increases presynaptic Pr, possibly by enhancing presynaptic Ca^2+^ transients.

### Ppp2r1a haploinsufficiency increases MAGL expression.

**Figure 5:**
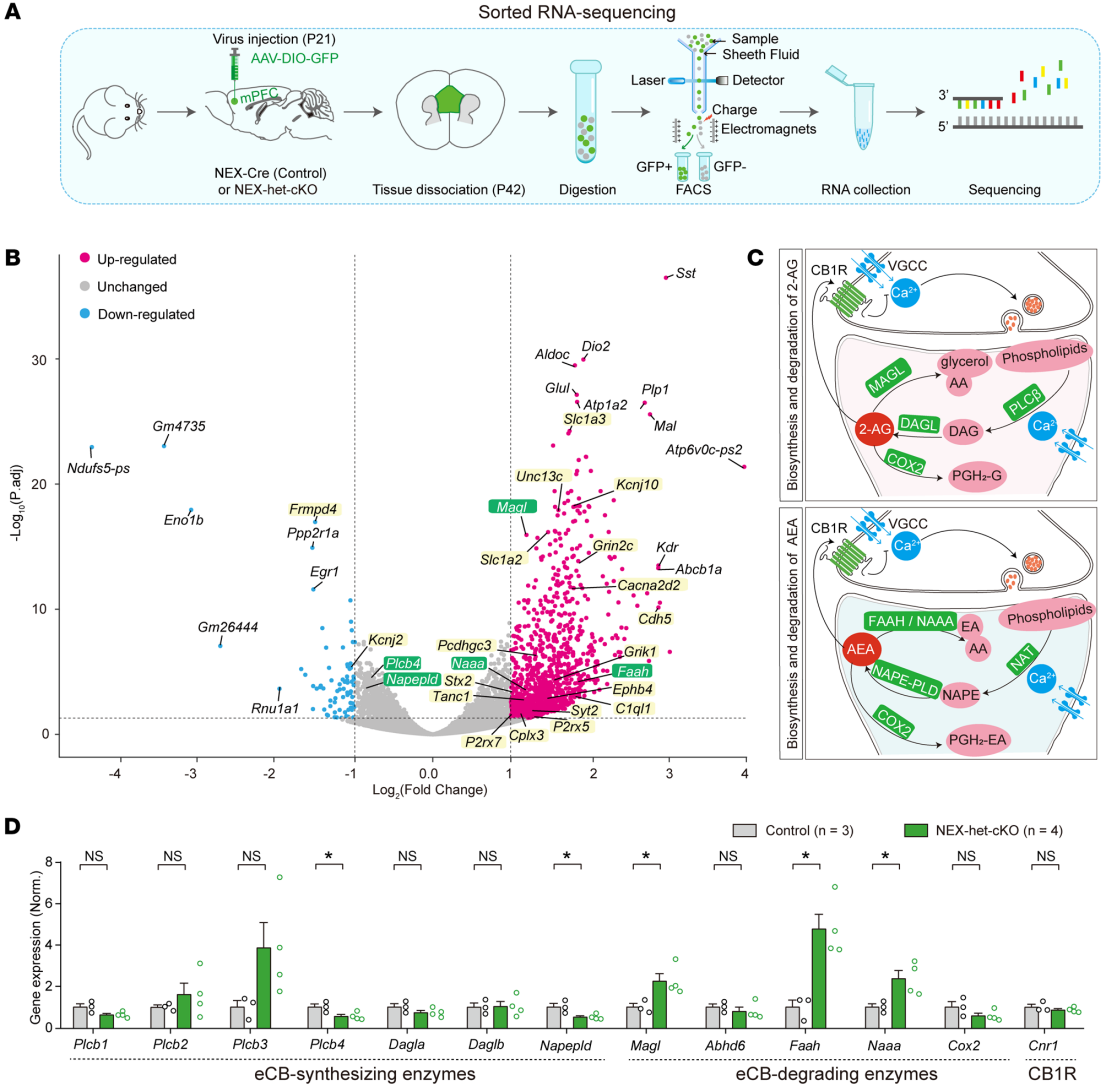
*Ppp2r1a* haploinsufficiency alters the transcription of eCB enzymes. (**A**) Workflow of sorted RNA-Seq experiments. (**B**) Volcano plot showing transcriptomic changes in NEX^+^ neurons of NEX-het-cKO mice. Pink dots represent upregulated DEGs with log_2_ (fold change) > 1 and *P*.adj < 0.05, and blue dots represent downregulated DEGs with log_2_ (fold change) < –1 and *P*.adj < 0.05 (green, eCB-associated enzymes; yellow, synaptic molecules). (**C**) Schematic of key enzymes involved in biosynthesis and degradation of 2-AG and AEA. VGCC, voltage-gated calcium channel; DAG, diacylglycerol, PGH2-G, prostaglandin H2-glycerol ester; PGH2-EA, Prostaglandin H2-ethanolamide; AA, arachidonic acid; EA, ethanolamine; NAT, N-acetyltransferase. (**D**) Bar graph showing mRNA expression levels (transcripts per million) of enzymes involved in eCB synthesis and degradation, alongside CB1R, based on sorted RNA-Seq of NEX^+^ neurons from control (*n* = 3) and NEX-het-cKO (*n* = 4) mice. *Abhd6*: α/β hydrolase domain containing 6; *Cnr1*, cannabinoid receptor 1. Statistical comparisons were performed using the negative binomial distribution model of DESeq2 (**B**) (also see Supplemental Table 1) and 2-tailed unpaired Student’s *t* test (**D**). All data are presented as mean ± SEM. *P < 0.05.

The behavioral abnormalities and synaptic dysfunctions in NEX-het-cKO mice may result from molecular changes. To reveal the transcriptional changes caused by *Ppp2r1a* haploinsufficiency in NEX^+^ neurons, we injected AAV-Ef1α-DIO-EYFP into the mPFC of NEX-Cre (control) and NEX-het-cKO mice and isolated EYFP^+^ cells (presumably NEX^+^ glutamatergic neurons) by FACS for subsequent RNA-Seq (sorted RNA-Seq) (Figure 5A). Principal component analysis and correlation heatmaps revealed clear transcriptional signatures between genotypes (Supplemental Figure 10, A and B). Of the 15,878 genes detected, 1,160 upregulated and 73 downregulated differentially expressed genes (DEGs) were identified, with | log_2_ (fold change) | > 1 and *P*.adj < 0.05 (Figure 5B and Supplemental Figure 10C). These DEGs clearly separated the 2 groups, supporting the robustness of the transcriptional differences (Supplemental Figure 10D). Functional enrichment analyses revealed that upregulated DEGs were associated with synapse organization and neurotransmitter regulation (Supplemental Figure 10E and Supplemental Table 1), while downregulated DEGs were mainly associated with presynaptic/postsynaptic cytosol, spines, and long-term memory (Supplemental Figure 10F and Supplemental Table 1). These results suggest that *Ppp2r1a* haploinsufficiency altered the expression of over 1,000 genes, likely contributing to synaptic transmission alterations and behavioral deficits.

The major eCBs in the brain, 2-AG and AEA, function as retrograde signals that bind to presynaptic CB1Rs and inhibit neurotransmitter release by suppressing presynaptic Ca^2+^ influx (39–42). The synthesis of 2-AG is primarily mediated by phospholipase C-β (PLC-β) and DAGLα, while its degradation involves MAGL and cyclooxygenase-2 (COX2) (22, 43) (Figure 5C). Similarly, AEA is synthesized mainly by *N*-acyl-phosphatidylethanolamine-hydrolyzing phospholipase D (NAPE-PLD) and degraded by fatty acid amide hydrolase (FAAH), *N*-acylethanolamine acid amidase (NAAA), and COX2 (22, 43) (Figure 5C). Given the central roles of these enzymes in regulating eCB signaling (43, 44), we examined whether *Ppp2r1a* haploinsufficiency alters their expression. Sorted RNA-Seq revealed altered expression of several eCB-related genes, including upregulation of 3 eCB-degrading enzymes (*Magl*, *Faah*, and *Naaa*) and downregulation of 2 eCB-synthesizing enzymes (*Plcb4* and *Napepld*) (Figure 5, B and D). These findings suggest that *Ppp2r1a* haploinsufficiency may alter the expression of key eCB-related enzymes.

**Figure 6:**
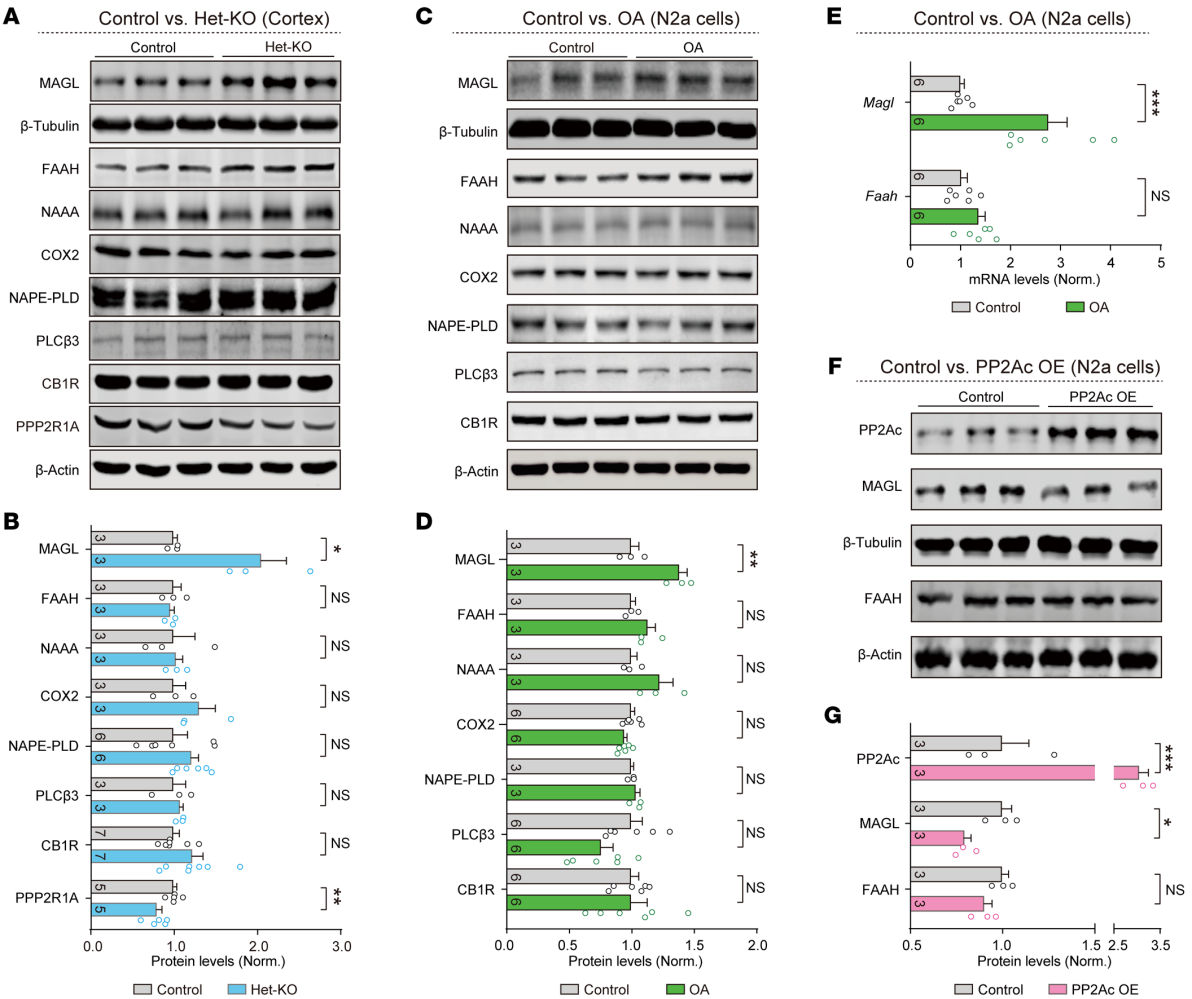
*Ppp2r1a* haploinsufficiency increases MAGL expression. (**A** and **B**) Western blot analysis showing increased MAGL protein level in het-KO mice. β-Tubulin was used as the loading control for MAGL protein quantification, and β-actin was used for all other proteins. (**C** and **D**) Western blot analysis showing increased MAGL protein level in N2a cells treated with 10 nM OA. (**E**) Reverse transcription qPCR analysis showing increased *Magl* mRNA level in OA-treated N2a cells. (**F** and **G**) Western blot analysis showing that overexpression of PP2Ac in N2a cells reduced MAGL, but not FAAH protein levels. Numbers of mice (**B**) or N2a experiments (**D**, **E**, and **G**) for both genotypes are indicated in the graphs. Statistical comparisons were performed using 2-tailed unpaired Student’s *t* test (**B**, **D**, **E**, and **G**). All data are presented as mean ± SEM. *P < 0.05, **P < 0.01, ***P < 0.001.

To validate the protein levels of these enzymes in *Ppp2r1a*-deficient neurons, we crossed CMV-Cre mice with *Ppp2r1a* cKO mice to generate constitutive *Ppp2r1a*^+/–^ (het-KO) mice, in which all cells were *Ppp2r1a* haploinsufficient. Cortical lysates of het-KO mice showed elevated MAGL protein levels (Figure 6, A and B), with no detectable changes in other eCB enzymes. To further validate this finding, we used serum-starved differentiated Neuro 2a (N2a) cells (45) and found that inhibition of PP2A by okadaic acid (OA), a potent inhibitor of PP2A (46), selectively increased MAGL protein levels, without detectable changes in FAAH, NAAA, COX2, NAPE-PLD, PLCβ3, and CB1R (Figure 6, C and D). In addition, *Magl* mRNA levels similarly increased in OA-treated N2a cells (Figure 6E). Conversely, overexpression of PP2Ac, the catalytic subunit of PP2A, reduced MAGL expression (Figure 6, F and G), indicating bidirectional PP2A-dependent regulation. Taken together, these findings suggest that *Ppp2r1a* haploinsufficiency increases MAGL expression.

### PPP2R1A regulates Magl transcription in an EZH2-dependent manner.

**Figure 7:**
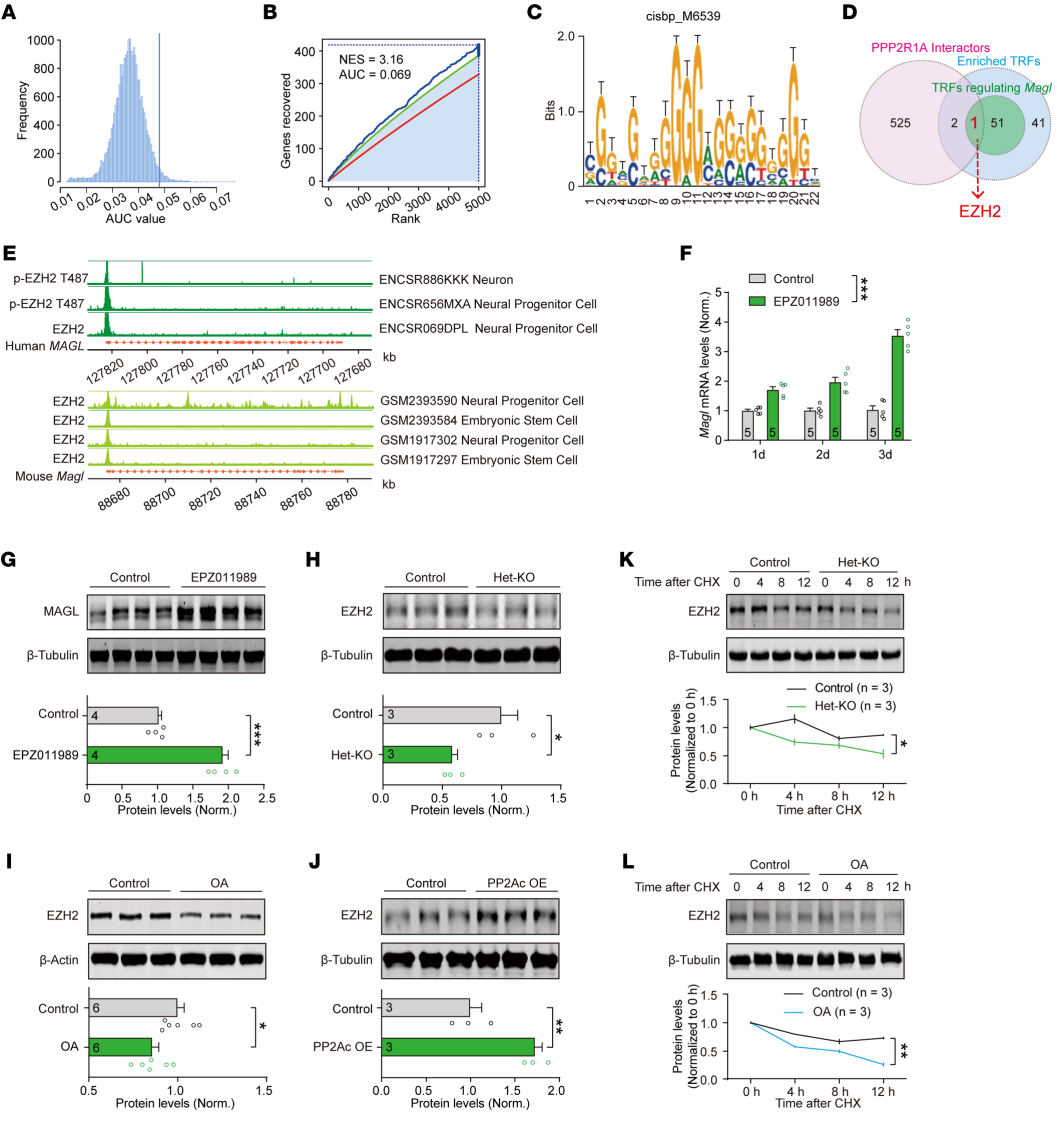
PPP2R1A regulates *Magl* transcription in an EZH2-dependent manner. (**A**) The distribution of AUC through motif enrichment analyses using sorted RNA-Seq data. Vertical line denoting the AUC threshold (AUC > mean + 2 × SD). (**B**) The recovery curve of motif enrichment analyses. The red line is the averaged recovery curve of all motifs, the green line represents mean + 2 × SD, and the blue line is the recovery curve of cisbp_M6539. The maximum enrichment level between cisbp_M6539 and the green curve is indicated by dotted line. (**C**) Possible sequence of the enriched motif cisbp_M6539. (**D**) Intersectional analyses identified EZH2 as the candidate TRF that both interacts with PPP2R1A and regulates *Magl* transcription. Pink circle, PPP2R1A-interacting proteins from BioGRID database; blue circle, predicted TRFs that regulate the DEGs; green circle, TRFs likely to regulate *Magl* transcription. (**E**) ChIP-Seq analyses showed EZH2 binding to the *MAGL*/*Magl* promoter, as identified by IDR (irreproducible discovery rate) < 0.05. (**F**) EPZ011989 (250 nM) treatment increased *Magl* mRNA levels. (**G**) Immunoblots revealing that EPZ011989 treatment increased MAGL protein levels. (**H**–**J**) Immunoblots revealing that PPP2R1A bidirectionally regulated the EZH2 expression. (**H**) EZH2 expression was reduced in het-KO mice. (**I**) OA (10 nM) treatment decreased EZH2 expression in N2a cells. (**J**) PP2Ac overexpression enhanced EZH2 expression in N2a cells. (**K** and **L**) PPP2R1A deficiency impaired EZH2 stability. (**K**) Cycloheximide (CHX; 300 μg/mL) chase assay showed accelerated EZH2 degradation in het-KO mice. (**L**) Same as **K** but for N2a cells treated with OA. Numbers of mice (**H** and **K**) or N2a experiments (**F**, **G**, **I**, **J**, and **L**) for both genotypes are indicated in the graphs. Statistical comparisons were performed using 2-tailed unpaired Student’s *t* test (**G**–**J**) and 2-way ANOVA followed by 2-tailed Bonferroni’s *t* test (**F**, **K**, and **L**). All data are presented as mean ± SEM. *P < 0.05, **P < 0.01, ***P < 0.001.

Our results show that both the mRNA and protein levels of MAGL are elevated in NEX-het-cKO mice (Figures 5 and 6). We hypothesize that PPP2R1A deficiency upregulates *Magl* transcription, potentially through disruption of specific transcription regulatory factors (TRFs). To identify candidate TRFs regulating DEGs, we performed a comprehensive motif analysis of promoter-proximal regions using the RcisTarget algorithm (47). Screening 24,453 known motifs against sorted RNA-Seq data revealed 72 significantly enriched motifs (area under the curve [AUC] > mean + 2SD; normalized enrichment score [NES] > 3) (Figure 7, A and B). These motifs corresponded to 95 TRFs (Figure 7D and Supplemental Table 2), with 52 potentially regulating *Magl* transcription (Figure 7D; Supplemental Table 2). Subsequent BioGRID database (48) screening of PPP2R1A-interacting proteins via coimmunoprecipitation and mass spectrometry yielded 528 candidates (Figure 7D and Supplemental Table 2). Intersectional analysis pinpointed EZH2, the enzymatic component of the Polycomb Repressive Complex 2 (49), as a potential interactor that may regulate *Magl* transcription via the cisbp_M6539 motif (Figure 7, C and D, and Supplemental Figure 11). Independent validation using overrepresentation analysis of ENCODE TRF ChIP-Seq gene set library (50) confirmed significant EZH2 binding to DEGs (Supplemental Figure 12, A and B, and Supplemental Table 3). Given that EZH2-mediated histone modifications are typically associated with transcription repression (49), our findings suggest that PPP2R1A may regulate *Magl* transcription through an EZH2-dependent mechanism.

To further validate whether EZH2 directly binds to the *Magl* promoter, we integrated 7 EZH2 ChIP-Seq experiments from human and mouse neuronal cells. All datasets consistently confirmed EZH2 binding to the *Magl* promoter (Figure 7E). While the ENCODE library also identified estrogen receptor 1 (ESR1) and E1A binding protein P300 (EP300) as enriched TRFs, neither showed marked *Magl* promoter binding (Supplemental Figure 12C). Therefore, our analyses suggest that PPP2R1A may regulate *Magl* transcription through an EZH2-dependent mechanism.

To investigate whether EZH2 regulates *Magl* transcription, we treated N2a cells with EPZ011989, a potent and selective EZH2 inhibitor (51), and observed an upregulation of both *Magl* mRNA (Figure 7F) and protein levels (Figure 7G), confirming that EZH2 can repress *Magl* transcription under physiological conditions.

Previous studies indicated that PP2A directly interacts with EZH2, and inhibiting PP2A reduces the protein abundance of EZH2, thereby attenuating its regulatory function on multiple genes (52). To verify whether *Ppp2r1a* haploinsufficiency influences EZH2 expression, we analyzed EZH2 protein levels in het-KO mice and observed a significant reduction (Figure 7H). Pharmacological inhibition of PP2A with OA in N2a cells similarly decreased EZH2 levels (Figure 7I). Conversely, increasing PP2A activity by PP2Ac overexpression elevated EZH2 levels (Figure 7J), indicating dynamic regulation of EZH2 by PP2A. Given that PP2A physically interacts with EZH2 (52) and that dephosphorylation of EZH2 could modulate its function (53), we hypothesized that reduced PP2A activity in het-KO mice might destabilize EZH2. Using the cycloheximide chase assay to inhibit protein synthesis, we found that both *Ppp2r1a* haploinsufficiency and inhibition of PP2A by OA accelerated the degradation of EZH2 (Figure 7, K and L). These data collectively suggest that *Ppp2r1a* haploinsufficiency decreases EZH2 stability, potentially leading to increased *Magl* transcription.

### Ppp2r1a haploinsufficiency impairs eCB release.

**Figure 8:**
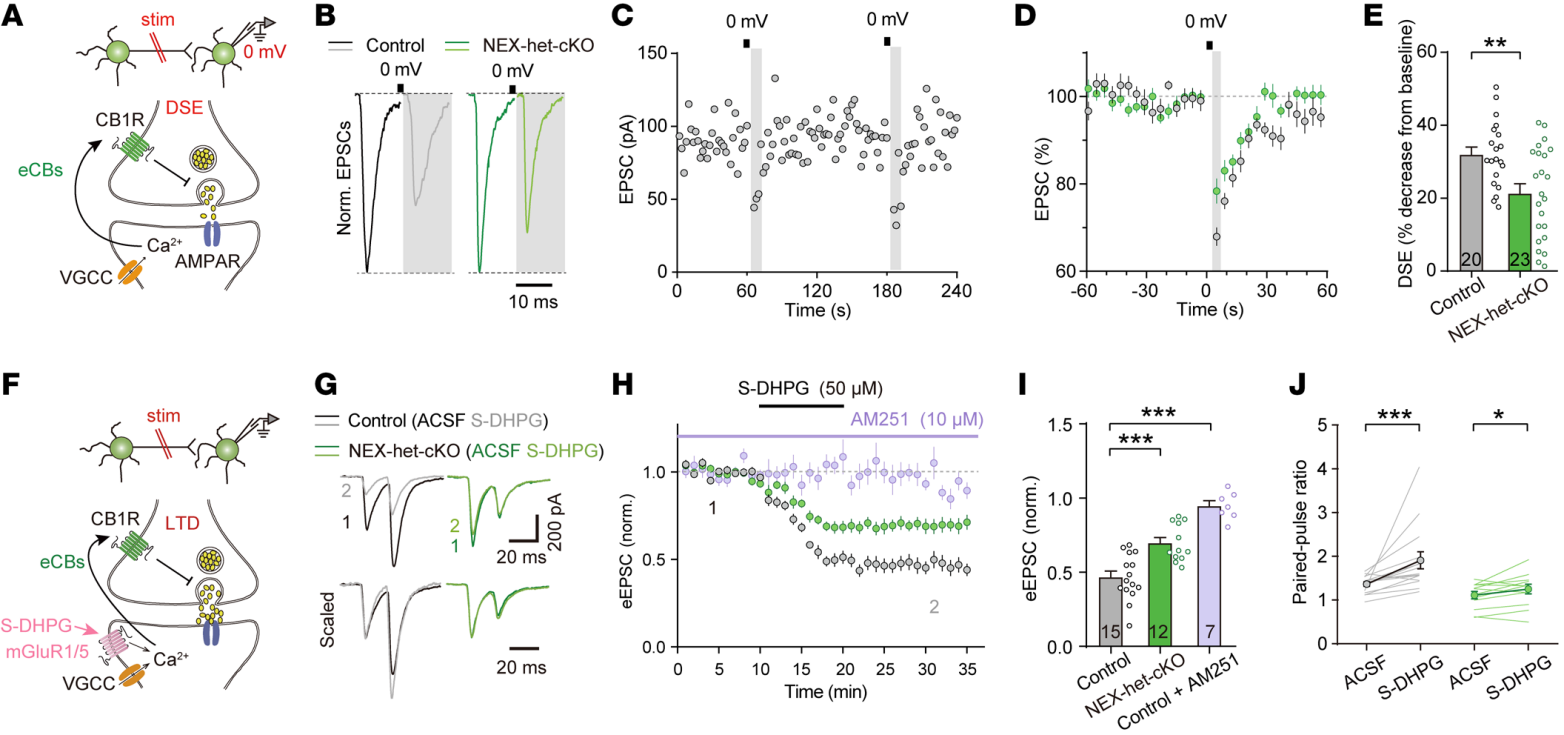
*Ppp2r1a* haploinsufficiency impairs eCB-dependent synaptic plasticity at excitatory synapses. (**A–E**) *Ppp2r1a* haploinsufficiency impaired the strength of DSE (Control, *n* = 20 neurons; NEX-het-cKO, *n* = 23 neurons). (**A**) Schematic of DSE experiments. Top, whole-cell recordings from L5 pyramidal neurons during induction of DSE; bottom, DSE induction pathway in response to depolarization. (**B**) Representative EPSC traces before and after DSE induction. The black line represents depolarization (0 mV, 5 s). (**C**) Representative DSE experiment performed in control neurons. (**D**) Time-course summary of normalized EPSC amplitude during the DSE experiment. (**E**) DSE amplitude was decreased in NEX-het-cKO mice. (**F**–**J**) *Ppp2r1a* haploinsufficiency impaired eCB-LTD (Control, *n* = 15 neurons; NEX-het-cKO, *n* = 12 neurons; Control + AM251, *n* = 7 neurons). (**F**) Schematic of the eCB-LTD experiment. (**G**) Representative EPSC traces before and after S-DHPG (50 μM) application. (**H**) Normalized time-course summary of S-DHPG–mediated eCB-LTD in both genotypes. Note that the CB1R antagonist AM251 (10 μM) blocked this eCB-LTD. (**I**) Summary graph showing that eCB-LTD was impaired in NEX-het-cKO mice and blocked by AM251. (**J**) Summary graph showing that EPSC PPRs were increased in both control and NEX-het-cKO mice, suggesting that eCB-LTD was mediated by a reduced presynaptic Pr. Statistical comparisons were performed using 2-tailed unpaired Student’s *t* test (**E** and **J**), 2-tailed paired Student’s *t* test (**J**), 1-way ANOVA followed by 2-tailed Bonferroni’s *t* test (**I**), 2-tailed Wilcoxon’s test (**J**), and 2-tailed Mann-Whitney test (**J**). All data are presented as mean ± SEM. *P < 0.05, **P < 0.01, ***P < 0.001.

Given that *Ppp2r1a* haploinsufficiency increased MAGL expression, we hypothesized that the elevated excitatory synaptic transmission in NEX-het-cKO mice might result from dysregulated eCB signaling. To test this, we recorded EPSCs evoked by electrical stimuli and measured depolarization-induced suppression of excitation (DSE) (39), a proxy for eCB secretion, in mPFC L5 pyramidal neurons (Figure 8A). While depolarization (0 mV for 5 s) (39) transiently suppressed EPSCs in both genotypes (Figure 8, B–D), the magnitude of DSE was significantly reduced in NEX-het-cKO neurons compared with controls (Figure 8E), indicating impaired eCB signaling.

We further conducted an eCB-mediated long-term depression (eCB-LTD) experiment using the group I mGluR agonist (*S*)-3,5-dihydroxyphenylglycine (S-DHPG; 50 μM) (54). S-DHPG reduced EPSCs by approximately 55% in control neurons, but only by approximately 30% in NEX-het-cKO neurons (Figure 8, F–I). Additionally, the inhibitory effect of S-DHPG was blocked by the CB1R antagonist AM251 (Figure 8, H and I), confirming the involvement of eCB in this form of synaptic plasticity. Moreover, EPSC PPRs significantly increased in control neurons after eCB-LTD, but this increase was attenuated in NEX-het-cKO neurons, suggesting impaired eCB modulation of presynaptic Pr (Figure 8J). These findings suggest that eCB signaling is impaired at excitatory synapses in NEX-het-cKO mice.

**Figure 9:**
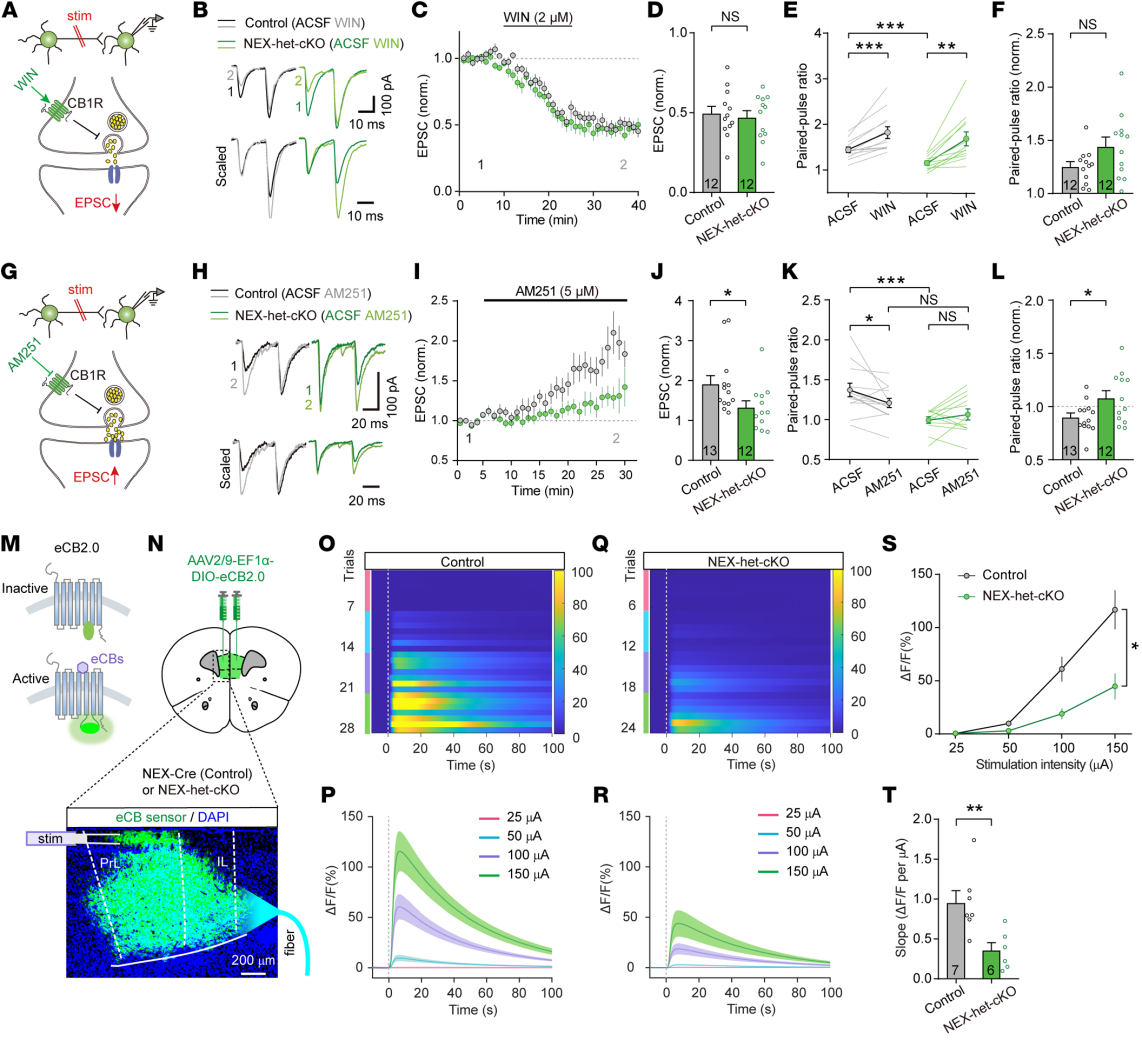
*Ppp2r1a* haploinsufficiency decreases eCB signaling in excitatory synapses. (**A**–**F**) CB1R activation exerted identical effects in both genotypes (Control, *n* = 12 neurons; NEX-het-cKO, *n* = 12 neurons). (**A**) Schematic of the WIN experiment. (**B**) Representative EPSCs before and after WIN (2 μM) application. (**C**) Normalized time-course summary of EPSC amplitude. (**D**) Unchanged WIN-mediated inhibition of EPSC amplitude. (**E**) WIN increased EPSC PPRs equally across both genotypes. (**F**) Normalized PPRs in both genotypes. (**G**–**L**) *Ppp2r1a* haploinsufficiency impaired eCB signaling (Control, *n* = 13 neurons; NEX-het-cKO, *n* = 12 neurons). (**G**) Schematic of AM251 experiment. (**H**) Representative EPSCs before and after AM251 (5 μM) application. (**I**) Normalized time-course summary of EPSC amplitude. (**J**) Effect of AM251 on EPSC amplitude was reduced in NEX-het-cKO mice. (**K**) AM251-mediated reduction in PPR was blocked in NEX-het-cKO mice. (**L**) Normalized PPRs in both genotypes. (**M**–**T**) GRAB_eCB2.0_ signals were significantly reduced in NEX-het-cKO mice (Control, *n* = 7 mice; NEX-het-cKO, *n* = 6 mice). (**M**) Schematic of GRAB_eCB2.0_ activation. (**N**) Schematic of viral injection and fiber photometry recordings of GRAB_eCB2.0_ signals in the mPFC. Scale bar: 200 μm. (**O** and **P**) Electrical stimulation evoked transient increases in GRAB_eCB2.0_ signals in controls. (**O**) Heatmap of aligned GRAB_eCB2.0_ signals from individual mice, with stimulation intensities indicated by colors. (**P**) Average traces of GRAB_eCB2.0_ signals across all controls. (**Q** and **R**) Same as **O** and **P** but for NEX-het-cKO mice. (**S**) Input/output plot of GRAB_eCB2.0_ signals versus stimulation intensity. (**T**) Slope of input/output relationship. Statistical comparisons were performed using 2-tailed unpaired Student’s *t* test (**D**, **E**, **K**, **L**, and **T**), 2-tailed unpaired Student’s *t* test with Welch’s correction (**F**), 2-tailed Mann-Whitney test (**E**, **J**, and **K**), 2-tailed paired Student’s *t* test (**E** and **K**), 2-tailed Wilcoxon’s test (**E** and **K**), and 2-way ANOVA followed by Bonferroni’s post hoc test (**S**). All data are presented as mean ± SEM. *P < 0.05, **P < 0.01, ***P < 0.001.

Given the reduction in DSE and eCB-LTD, we proposed that impaired eCB signaling may be due to defective CB1R function or reduced eCB release. To test CB1R function, we measured the effects of the CB1R agonist WIN55,212 (WIN) on EPSCs (Figure 9, A–D). In both control and NEX-het-cKO mice, WIN similarly attenuated EPSCs and increased EPSC PPRs (Figure 9, B–F), indicating intact CB1R function in excitatory synapses. To investigate eCB release, we applied the CB1R antagonist AM251 in the bath (Figure 9G) and found that EPSC amplitude nearly doubled in control mice post-AM251 application, while the increase in EPSC amplitude was significantly weaker in NEX-het-cKO mice (Figure 9, H–J). Furthermore, the effect of AM251 on the EPSC PPRs was significantly weaker in NEX-het-cKO mice (Figure 9, H, K, and L). Importantly, AM251 treatment reduced the difference in EPSC PPRs between control and NEX-het-cKO mice (Figure 9K). These findings suggest that reduced eCB release in NEX-het-cKO mice mediates the increased Pr of excitatory synapses observed in these mice.

eCB signaling has been extensively characterized in inhibitory synapses formed by cholecystokinin-positive (CCK-positive) GABAergic neurons (55), which represent only approximately 5% of cortical GABAergic neurons (56). Given our observation of reduced inhibitory transmission in NEX-het-cKO mice (Figure 2, J–L), we asked whether altered eCB signaling contributes to this phenotype. To assess CB1R function in inhibitory synapses, we recorded the IPSCs in L5 pyramidal neurons following perisomatic stimulation and applied WIN in the bath (Supplemental Figure 13, A and B). WIN similarly suppressed IPSCs (Supplemental Figure 13, B–D) and increased IPSC PPRs (Supplemental Figure 13, E and F) in both genotypes, indicating normal CB1R function in inhibitory synapses. We next examined the effect of tonic eCB release on IPSCs by applying AM251 (Supplemental Figure 13, G and H). However, AM251 had no significant effect on either IPSC amplitude or PPR in either group (Supplemental Figure 13, H–L), suggesting that tonic eCB signaling is minimal at inhibitory synapses under baseline conditions. These results suggest that altered eCB signaling is unlikely to contribute to the reduced inhibitory synaptic transmission observed in NEX-het-cKO mice.

To directly assess eCB release, we used a genetically encoded fluorescent sensor specifically designed for eCBs (GRAB_eCB2.0_, also referred to as eCB2.0) (57) and bilaterally injected AAV-DIO-eCB2.0 into the mPFC of NEX-Cre (control) and NEX-het-cKO mice (Figure 9, M and N). Four weeks after viral injection, we recorded the fluorescent GRAB_eCB2.0_ signals from mPFC slices using fiber photometry during 50 Hz electrical stimulation (Figure 9, O–R). AM251 completely abolished the evoked signal, confirming its specificity (Supplemental Figure 14, A, B, and G). Both AEA and 2-AG significantly enhanced the GRAB_eCB2.0_ signal (Supplemental Figure 14, C–F and H), further validating the sensor’s specificity. The amplitude of the GRAB_eCB2.0_ signal increased with stimulation intensity (Figure 9, O–S), indicating depolarization-dependent eCB release. Strikingly, NEX-het-cKO mice exhibited a significant reduction in GRAB_eCB2.0_ signal amplitude compared with controls (Figure 9, S and T). Together with the electrophysiological results, these findings suggest that the increased presynaptic Pr observed at excitatory synapses in NEX-het-cKO mice is likely due to impaired eCB release.

### Reduced 2-AG release mediates increased presynaptic Pr in NEX-het-cKO mice.

**Figure 10:**
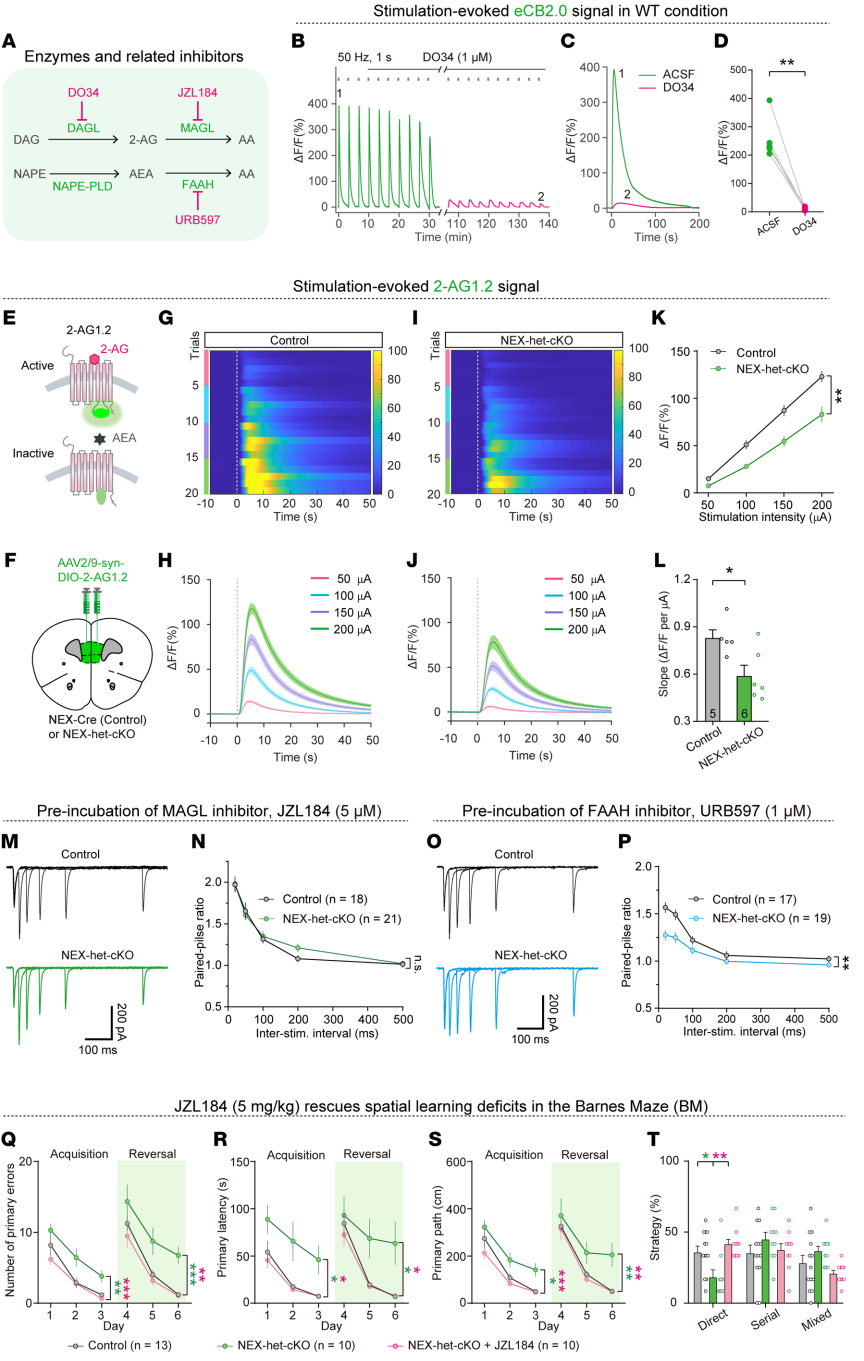
Reduced 2-AG release mediates increased presynaptic Pr and learning deficits in NEX-het-cKO mice. (**A**) Diagram showing synthesis and degradation pathways of 2-AG and AEA, together with related inhibitors. (**B**–**D**) The 2-AG was the principal eCB evoked by electrical stimulation. Representative traces (**B** and **C**) and summary (**D**) showing that DO34 (1 μM) abolished GRAB_eCB2.0_ signals (*n* = 5 mice). (**E**–**L**) The 2-AG release was significantly reduced in NEX-het-cKO mice (Control, *n* = 5 mice; NEX-het-cKO, *n* = 6 mice). (**E**) Schematic of GRAB_2-AG1.2_ activation by 2-AG but not by AEA. (**F**) Schematic of AAV-Syn-DIO-2-AG1.2 injection in the mPFC of NEX-Cre (Control) or NEX-het-cKO mice. Heatmap (**G**) and average traces (**H**) of GRAB_2-AG1.2_ signals evoked by increasing stimulation intensities in control. (**I** and **J**) Same as in **G** and **H** but for NEX-het-cKO mice. (**K**) Input/output plot of GRAB_2-AG1.2_ signals versus stimulation intensity. (**L**) Slope of input/output relationship. (**M** and **N**) MAGL inhibitor JZL184 rescued the reduced EPSC PPRs in NEX-het-cKO mice (Control, *n* = 18 neurons; NEX-het-cKO, *n* = 21 neurons). (**O** and **P**) FAAH inhibitor URB597 did not affect the reduced EPSC PPRs in NEX-het-cKO mice (Control, *n* = 17 neurons; NEX-het-cKO, *n* = 19 neurons). (**Q**–**T**) JZL184 ameliorated spatial learning and memory deficits in NEX-het-cKO mice (Control, *n* = 13 mice; NEX-het-cKO, *n* = 10 mice; NEX-het-cKO + JZL184, *n* = 10 mice). Green asterisks, control versus NEX-het-cKO; red asterisks, NEX-het-cKO versus NEX-het-cKO + JZL184. (**Q**) Number of primary errors. (**R**) Primary latency. (**S**) Primary path length. (**T**) Percentage of strategy used. Statistical comparisons were performed using 2-tailed unpaired Student’s *t* test (**L**), 2-tailed paired Student’s *t* test (**D**), 1-way ANOVA followed by 2-tailed Bonferroni’s *t* test (Serial and Mixed; **T**), Kruskal-Wallis test followed by Dunn’s test (Direct; **T**), and 2-way ANOVA followed by Bonferroni’s post hoc test (**K**, **N**, **P**, and **Q**–**S**). Data are presented as mean ± SEM. *P < 0.05, **P < 0.01, ***P < 0.001.

Given the impairment of eCB release in NEX-het-cKO mice, we investigated the specific contributions of 2-AG and AEA to this phenomenon. Both RNA-Seq and Western blotting indicated increased MAGL expression in NEX-het-cKO mice (Figure 5D and Figure 6, A and B), potentially contributing to reduced 2-AG signaling. To explore this hypothesis, we perfused the selective DAGLα inhibitor DO34 in artificial cerebrospinal fluid (ACSF) (57, 58), which blocks 2-AG biosynthesis, and tested its effects on GRAB_eCB2.0_ signals (Figure 10, A–D). Notably, DO34 treatment nearly abolished the fluorescent GRAB_eCB2.0_ signals (Figure 10, B–D), suggesting that these signals primarily reflect 2-AG release rather than AEA.

To further validate this, we used 2 recently developed genetically encoded sensors, namely, GRAB_2-AG1.2_ and GRAB_AEA1.2_ (or simply 2-AG1.2 and AEA1.2) (Figure 10E and Supplemental Figure 16A), which were engineered from CB1Rs with targeted mutations (unpublished observations). These sensors selectively detect the release of 2-AG and AEA, respectively (Figure 10E and Supplemental Figure 16A). We bilaterally injected AAV-Syn-DIO-2-AG1.2 and AAV-CaMKII-AEA1.2 into the mPFC of NEX-Cre mice (Figure 10F and Supplemental Figure 16B) and assessed sensor specificity in acute mPFC slices. Puff application of 2-AG evoked a robust increase in the GRAB_2-AG1.2_ signal (13.74% ± 4.78%) (Supplemental Figure 15, C, D, and H) but only a slight increase in the GRAB_AEA1.2_ signal (4.15% ± 2.92%) (Supplemental Figure 16, C, E, and F). In contrast, AEA application elicited a strong response from GRAB_AEA1.2_ (16.27% ± 2.84%) (Supplemental Figure 16, D–F) but only a weak response from GRAB_2-AG1.2_ (3.02% ± 0.89%) (Supplemental Figure 15, E, F, and H), confirming the high specificity of both sensors. Furthermore, the GRAB_2-AG1.2_ response to electrical stimulation was abolished by AM251 (Supplemental Figure 15, A, B, and G), validating its specificity.

To compare 2-AG release between control and NEX-het-cKO mice, we bilaterally injected AAV-Syn-DIO-2-AG1.2 into the mPFC of NEX-Cre (control) or NEX-het-cKO mice at P21 (Figure 10F). Four weeks after injection, fiber photometry recordings revealed significantly attenuated GRAB_2-AG1.2_ signals in the mPFC of NEX-het-cKO mice upon local electrical stimulation (Figure 10, G–L). In parallel, AAV-CaMKII-AEA1.2 was bilaterally injected into the mPFC of both groups (Supplemental Figure 16B). However, electrical stimulation failed to evoke detectable GRAB_AEA1.2_ signals in both genotypes (Supplemental Figure 16, G–I). These results further support the conclusion that 2-AG, rather than AEA, is the primary eCB released from pyramidal neurons in response to electrical stimulation and that this release is impaired in NEX-het-cKO mice.

Given the well-established involvement of the hippocampus in spatial learning, we hypothesized that excitatory synaptic function may also be altered in the hippocampus of NEX-het-cKO mice. To test this, we recorded PPRs of EPSCs in dorsal CA1 (dCA1) pyramidal neurons by stimulating the Schaffer collateral pathway (Supplemental Figure 17A). As expected, EPSC PPRs were significantly reduced in NEX-het-cKO mice (Supplemental Figure 17, B and C), indicating an increase in presynaptic Pr at CA3-CA1 excitatory synapses. To assess 2-AG signaling, we bilaterally injected AAV-Syn-DIO-2-AG1.2 into the dCA1 of NEX-Cre (control) or NEX-het-cKO mice and measured fluorescent GRAB_2-AG1.2_ signals upon Schaffer collateral stimulation (Supplemental Figure 18, A and B). Consistent with the cortical findings, GRAB_2-AG1.2_ signal was significantly reduced in NEX-het-cKO mice (Supplemental Figure 18, C–H), suggesting impaired 2-AG release in the hippocampus.

Collectively, these results support the conclusion that impaired 2-AG signaling contributes to the enhanced excitatory synaptic transmission and spatial learning deficits observed in the NEX-het-cKO mice, likely by broadly affecting excitatory synapses across the forebrain.

### MAGL inhibitor JZL184 rescues Ppp2r1a haploinsufficiency-induced memory deficits.

We subsequently investigated whether alterations in MAGL activity contribute to the increase in presynaptic Pr. Perfusion of JZL184, a potent MAGL inhibitor, eliminated the differences in EPSC PPRs between the control and NEX-het-cKO mice (Figure 10, M and N). In contrast, the application of the FAAH blocker URB597 had no effect on the reduced EPSC PPRs observed in NEX-het-cKO mice (Figure 10, O and P). JZL184 also effectively corrected the increased presynaptic Ca^2+^ transients found in NEX-het-cKO mice (Supplemental Figure 19). These findings suggest that reduced 2-AG signaling due to increased MAGL activity mediates the increase in presynaptic Pr in NEX-het-cKO mice.

To determine whether increased MAGL activity contributes to spatial learning and memory deficits, we administered JZL184 via intraperitoneal injection (59). Remarkably, JZL184 treatment restored memory performance in NEX-het-cKO mice, normalizing the error rates, latency, and distance traveled to locate the correct hole in the BM (Figure 10, Q–S). Additionally, JZL184 administration increased the proportion of NEX-het-cKO mice employing a direct strategy (Figure 10T). These findings suggest that suppressing MAGL activity is sufficient to rescue the spatial learning and memory deficits observed in NEX-het-cKO mice.

## Discussion

Our research demonstrated that *Ppp2r1a* haploinsufficiency in forebrain excitatory neurons diminished anxiety levels and impaired spatial learning and memory, highlighting its important role in these neurons. Furthermore, *Ppp2r1a* haploinsufficiency increased presynaptic Pr at excitatory synapses, attributed to reduced eCB signaling. Further analyses revealed a key molecular pathway involved in the regulation of eCB signaling, in which PPP2R1A represses the transcription of the 2-AG hydrolase MAGL through an EZH2-dependent mechanism. These findings provide fresh insights into the molecular and cellular mechanisms underpinning NDDs.

### Ppp2r1a deficiency is linked to intellectual disability.

Protein phosphorylation and dephosphorylation are fundamental posttranslational modifications in cells (60), with PP2A playing a pivotal role in dephosphorylation (61). Dysregulated PP2A activity — resulting from altered catalytic activity, subunit expression, methylation, and/or phosphorylation (62) — has been implicated in tau hyperphosphorylation (63) and amyloidogenesis (64), both of which are central pathological processes in neurodegenerative diseases (13, 65).

Clinical studies have linked heterozygous loss-of-function mutations in PP2A subunits, including *PPP2R1A* and *PPP2R5D*, to intellectual disability (5–9), though the underlying mechanisms remain unclear. We confirmed that *Ppp2r1a* haploinsufficiency in mice recapitulates spatial learning and memory deficits (Figure 1, F–O), validating this model for studying human intellectual disability. Our study establishes a causal link between *Ppp2r1a* haploinsufficiency and intellectual disability. Notably, *Ppp2r1a* haploinsufficiency restricted to forebrain excitatory neurons was sufficient to reproduce these impairments, underscoring the pivotal role of this neuronal population. We hypothesize that whole-brain heterozygous *Ppp2r1a*-knockout mice may exhibit additional symptoms due to dysfunction in other cell types (e.g., GABAergic neurons) or regions (e.g., subcortical structures), which we aim to explore in future studies. Together, these findings broaden the current understanding of PP2A’s role beyond neurodegenerative diseases to include NDDs and may inform future development of targeted therapeutic strategies.

### Synaptic dysfunction is a core mechanism underlying NDDs.

Since PP2A regulates the dephosphorylation of many substrate proteins (66), *Ppp2r1a* dysfunction may broadly disrupt phosphorylation homeostasis, extending beyond tau and amyloid. However, the precise mechanisms by which *Ppp2r1a* deficiency contributes to NDDs, particularly intellectual disability, remain poorly understood.

We demonstrated that *Ppp2r1a* haploinsufficiency enhances excitatory synaptic transmission and reduces inhibitory synaptic transmission (Figure 2, A–L), potentially leading to E/I imbalance (Figure 2P). This elevated excitatory synaptic transmission arises from increased presynaptic Pr (Figure 4), possibly due to impaired eCB signaling (Figures 8–10). Previous studies have shown that dysfunction in inhibitory synapses from parvalbumin-positive (PV^+^) neurons reduces mIPSC frequency in mPFC pyramidal neurons (67). Hence, the reduced mIPSC frequency observed in NEX-het-cKO mice may reflect altered PV^+^ neuron function (Figure 2, J–L), which preferentially innervates the perisomatic region of pyramidal neurons (68). Given that PV^+^ neurons lack CB1Rs (69), the observed inhibitory synaptic deficits are likely mediated by eCB-independent mechanisms, possibly involving disrupted pathways governing inhibitory synapse formation. Although we did not detect significant modulation of evoked IPSCs by tonic eCB signaling (Supplemental Figure 13), we cannot rule out a potential contribution from reduced 2-AG release at CCK^+^ inhibitory synapses, which do express CB1Rs (55). This possibility will be addressed in future studies.

Collectively, our findings support the hypothesis that synaptic dysfunction underlies the behavioral abnormalities observed in NDDs (70) and highlight the contribution of synaptic dysfunction to these deficits.

### Impaired eCB signaling in NDDs.

eCBs act as retrograde signals that suppress neurotransmitter release (22), and their dysregulation is linked to neuropsychiatric and neurodegenerative disorders (71). Although clinical studies have shown that genetic variants of eCB enzymes or abnormal serum eCB levels are significantly associated with NDDs (23–25), the underlying mechanisms remain poorly understood.

The strength of eCB signals can be modulated by changes in CB1R expression or function, as well as alterations in eCB synthesis and degradation. Prior studies have shown that elevating 2-AG levels can ameliorate social deficits in *Shank3B*-knockout mice (72), highlighting the therapeutic potential of targeting eCB signaling in ASD treatment. Our results demonstrated that *Ppp2r1a* haploinsufficiency upregulates MAGL expression (Figure 6, A and B), resulting in reduced eCB signaling (Figures 8 and 9). This impairment was attributed to a decrease in 2-AG release (Figure 10, E–L). Notably, treatment with the MAGL inhibitor JZL184 rescued both synaptic and behavioral abnormalities in NEX-het-cKO mice (Figure 10, M–T, and Supplemental Figure 19). These findings provide strong evidence that disrupted eCB signaling is linked to synaptic dysfunction and deficits in spatial learning and memory in NDD mouse models and that JZL184 treatment can effectively mitigate both deficits. Together with previous studies (26, 72), our work highlights the therapeutic potential of targeting eCB system for the treatment of NDDs.

*Does PPP2R1A regulate protein function by dephosphorylation or transcriptional regulation?* PP2A, a serine/threonine phosphatase in the brain, regulates the dephosphorylation of over 300 substrate proteins (17), with its subunit composition influencing substrate specificity (61). Previous studies have shown that neuronal activity can regulate the phosphorylation of various synaptic proteins, such as synaptobrevin, syntaxin 1A, SNAP25, synaptotagmin 1, RIM1, and Cacna1a (73). In addition, PP2A can affect transcription by dephosphorylating RNA Pol II (19). Our transcriptomic analysis by sorted RNA-Seq revealed 1,160 upregulated and 73 downregulated DEGs in NEX-het-cKO mice (Figure 5B and Supplemental Figure 10C), suggesting that PPP2R1A broadly influences transcription. Among the upregulated DEGs, somatostatin (Sst) — a marker for a subset of GABAergic neurons — was unexpectedly elevated (Figure 5B). However, reanalysis of the raw sequencing data revealed that Sst expression was extremely low in both genotypes (see Supplemental Table 1), suggesting that its apparent upregulation likely resulted from minor FACS contamination. Mechanistically, previous studies have shown that phosphatases can regulate EZH2 dephosphorylation at the S21 residue, thereby modulating its function (53). Phosphorylation at other residues (e.g., S734, T350, T372, T419, T492, and Y641) has been implicated in EZH2 stability and degradation (74–76). Hyperphosphorylation of EZH2 may promote EZH2 ubiquitination and subsequent proteasomal degradation (74–76). We therefore propose that *Ppp2r1a* haploinsufficiency impairs EZH2 dephosphorylation, thereby reducing its stability (Figure 7, K and L). This, in turn, dampens EZH2-mediated transcriptional repression of *Magl*, increasing MAGL expression (Figure 6, A–E) and subsequently reducing eCB signaling at excitatory synapses in NEX-het-cKO mice (Figures 8 and 9). Because mutations in multiple subunits, including *PPP2R1A*, *PPP2R5B*, *PPP2R5C*, and *PPP2R5D*, have been linked to intellectual disability (5–9), future studies will investigate their roles in eCB signaling and related neuropsychiatric disorders.

## Methods

### Sex as a biological variable.

We used only male mice in this study because laboratory mice exhibit sex- and strain-dependent behavioral parameters (77).

### Mice.

Mice were housed (3–5 mice per cage) with food and water provided ad libitum under a 12 h light/dark cycle. The following strains were used: (a) *Ppp2r1a*-cKO (JAX 017441), (b) NEX-Cre (MGI2668659) (31) (provided by Zilong Qiu, Shanghai Jiao Tong University, China with permission from Klaus Nave, Max Planck Institute, Goettingen, Germany), (c) CMV-Cre (JAX 006054), and (d) LSL-H2B-GFP (JAX 036761). The genotyping primers for these lines are listed in Supplemental Table 4. All animals were maintained on a C57BL/6 background. To obtain forebrain excitatory neuron–specific *Ppp2r1a* cKO (NEX-Cre; *Ppp2r1a*^f/+^, NEX-het-cKO) mice, *Ppp2r1a* cKO mice were bred with NEX-Cre mice. To obtain constitutive *Ppp2r1a* KO (*Ppp2r1a*^+/−^, het-KO) mice, *Ppp2r1a* cKO mice were bred with the CMV-Cre mice. All the details of the experimental designs are listed in Supplemental Table 5. More detailed information can be found in Supplemental Methods.

### Statistics.

All values in the text and figure legends are presented as mean ± SEM. All statistics were calculated using GraphPad Prism v8.0 (GraphPad Software Inc.) or R v4.2.2 (R Foundation). Data analysis was performed by experimenters blind to experimental conditions. Normality distribution of data was confirmed using the Shapiro-Wilk test, and the equality of variances of data was examined with the Brown-Forsythe test. Based on these assessments, appropriate parametric or nonparametric tests with post hoc analyses were applied for multiple comparisons as indicated. Parametric tests, such as 2-tailed paired or unpaired Student’s *t* test, or 1- or 2-way ANOVA tests followed by Bonferroni’s post hoc test, were used when the datasets met normality and equal variance criteria. For datasets that did not meet these criteria, nonparametric tests were employed, including 2-tailed unpaired Mann-Whitney test, Wilcoxon’s matched-pair signed rank test, Kruskal-Wallis test, and repeated-measure 2-way ANOVA tests with the Greenhouse-Geisser correction. Additionally, the Kolmogorov-Smirnov test, χ^2^ test, negative binomial distribution model of DESeq2, and 1-tailed Fisher’s exact test were used as required. All the experiments were conducted with at least 3 biological replicates. Details, including number of animals/neurons, *P* values, and statistical tests, are provided in the figures, figure legends, and Supplemental Table 6.

### Study approval.

All experimental procedures were conducted in accordance with the guidelines of the Animal Care and Use Committee of Huazhong University of Science and Technology, China (2018 IACUC 3976).

### Data availability.

All datasets in this study are included in this published article and its supplemental files. Values for all data points shown in graphs are reported in the Supporting Data Values file. Raw sequence data have been deposited in the Genome Sequence Archive at the National Genomics Data Center, China National Center for Bioinformation/Beijing Institute of Genomics, Chinese Academy of Sciences (CRA018198) and are publicly accessible at https://ngdc.cncb.ac.cn/gsa This paper does not report original code, and all software used in this study is publicly available.

## Author contributions

MJ conceived and, along with BX, supervised the project. YW, ZT, GD, and Liangpei Chen performed mouse breeding. YW performed slice electrophysiology, morphological construction, and immunohistochemical experiments. H Li performed virus injection and animal behavioral experiments. RC, SC, and YL developed the GRAB_2-AG1.2_ and GRAB_AEA1.2_ sensors. H Li and YW performed the fiber photometry recordings in mPFC brain slices. WD and YW performed the sorted RNA-Seq, N2a cell culture, and biochemistry experiments. YW, WD, H Li, and H Luo analyzed the data. Liping Chen provided the *Ppp2r1a*^fl/fl^ mice. JZW provided the PP2Ac plasmid and critical comments for this study. MJ and BX wrote the manuscript with input from all authors. The order of co-first authors (YW, WD, H Li) reflects the relative scope, depth, and centrality of their contributions. YW led key experiments, including electrophysiology, morphological studies, sequencing, and data analysis, and oversaw animal breeding and project progress. WD focused on bioinformatics analyses and collaborated in biochemical experiments, making critical contributions to elucidating the molecular mechanisms. H Li contributed primarily to virus injections, behavioral experiments, and fiber photometry recordings, providing important insights into the behavioral impact of Ppp2r1a haploinsufficiency. YW’s broader and more foundational role justifies her position first, followed by WD and H Li.

## Supplementary Material

Supplemental data

Unedited blot and gel images

Supplemental table 1

Supplemental table 2

Supplemental table 3

Supporting data values

## Version 1 08/21/2025

In-Press Preview

## Version 2 09/02/2025

Electronic publication
