# Supplementary material for: Ppp2r1a haploinsufficiency increases excitatory synaptic transmission and decreases spatial learning by impairing endocannabinoid signaling
Source: J Clin Invest. 2025 Aug 21;135(17):e185602. doi: 10.1172/JCI185602 (PMC12404745; doi:10.1172/JCI185602)
Supplement: Supplemental data [file jci-135-185602-s304.pdf]

Supplemental Figures for

Ppp2r1a Haploinsufficiency Increases Excitatory Synaptic Transmission and Decreases Spatial Learning by Impairing Endocannabinoid Signaling

Yirong Wang, Weicheng Duan, Hua Li, Zhiwei Tang, Ruyi Cai, Shangxuan Cai, Guanghao Deng, Liangpei Chen, Hongyan Luo, Liping Chen, Yulong Li, Jian-Zhi Wang, Bo Xiong, Man Jiang

The PDF file includes:

Supplemental Methods

Supplemental Figures 1 to 19

Supplemental Tables 4 to 6

Supplemental References

Supplemental Methods

Virus preparation

One adeno-associated virus (AAV) serotype (AAV2/9) was used. All AAVs (AAV-CAG-DIO-GCaMP6m, AAV-EF1α-DIO-eCB2.0, AAV-Syn-DIO-2AG1.2, AAV-CaMKII-AEA1.2, and AAV-EF1α-DIO-EYFP) were purchased from Shanghai Taitool Bioscience (China) or Brain Case (China). The final AAV genomic titers ranged from 2 to 8 × 10^{12} particles/mL. The AAVs are listed in Supplemental Table 4.

Stereotactic injection

Mice at postnatal day 21–25 (P21–P25) were anesthetized with tribromoethanol (150–250 mg/kg, i.p.), followed by the immobilization of their heads on a stereotaxic frame (RWD, Shenzhen, China) for viral injection. Standard surgery was performed to expose the brain surface over the mPFC, dorsal CA1 (dCA1), or ventral CA1(vCA1). Coordinates for the mPFC injection were: bregma +2.1 mm, lateral ±0.35 mm, and dura −2.0/−1.8 mm. Coordinates for the dCA1 injections were: bregma −1.66 mm, lateral ±1.2 mm, and dura −1.5 mm. Coordinates for the vCA1 injections were: bregma −3.0 mm, lateral ±3.1 mm, and dura −2.3/−3.7 mm. AAVs (300 nL) were stereotaxically injected using a glass micropipette connected to a Nanoliter Injector (MICRO-2T, World Precision Instruments, Inc., USA) at a slow flow rate of 100 nL/min. The pipette was held for a further 10 min after viral injection. The wounds were sutured, and the mice were kept warm on an electric heating pad until complete recovery from anesthesia. The mice were then returned to their home cages and housed until further experiments. Experiments related to viral injection are summarized in Supplemental Table 5.

Mouse behaviors

Preparation for behavioral tests. Prior to all behavioral tests, mice were handled in the test environment for 10 min daily for at least three days. The mice were also habituated to the test room for at least 30 min before the behavioral test commenced. To eliminate potential contamination from other mice, all experimental equipment was cleaned with 75% ethanol and dried before and between trials. All experiments were videotaped and analyzed using a tracking system (Biobserve, Germany).

Open field test (OFT). A white plastic box (40 cm × 40 cm × 40 cm) was used as the open field arena for the experiment. Mice were placed in the center of the arena and allowed to freely explore for 10 min. Total distance traveled, time spent in the center zone (20 cm × 20 cm), distance traveled in the center, and number of visits to the center were analyzed.

Elevated plus maze (EPM). The EPM behavioral apparatus consisted of two open arms (30 cm × 6 cm) and two closed arms (30 cm × 6 cm × 15 cm) raised 45 cm above the floor. Mice were placed in the center of the maze, facing one of the open arms, and allowed to explore freely for 5 min. The anxiety level was assessed by time spent and number of entries into the open arms.

T-maze. The T-maze consisted of a start arm (45 cm × 9 cm × 15 cm) and two target arms (30 cm × 9 cm × 15 cm) on either side. At the start of the experiment, a mouse was placed in the start area for 10 s, after which the gate opened to allow the mouse to freely explore both target arms. Upon complete entry into one of the goal arms, the mouse was confined there for 30 s with the gate closed. The mouse was then returned to its home cage before the next trial, which was repeated 11 times at 90-s intervals. The trial in which the test mouse chose an arm different from the

previous one was recorded as a successful trial.

Eight-arm radial maze (EAM). The EAM is a closed structure consisting of a regular octagonal central platform (6 cm on each side) and eight interconnected radiating arms (30 cm × 6 cm × 15 cm). Mice were subjected to food restriction to maintain body weight at 85% of baseline. Before the experiment commenced, mice underwent a 10-min habituation phase, during which they were permitted to freely explore the eight open arms, each of which had a peanut pellet placed at its end. Habituation was performed twice daily (4 h apart) for two consecutive days. No additional food other than the peanut pellets was provided throughout the experiment to maintain a motivational state. The experiment consisted of a training phase and test phase. During training, four non-adjacent arms were opened with a peanut pellet at their distal end. The mouse was placed on the central platform to freely explore for 5 min before being returned to its home cage. The eight-arm maze was then disinfected with 75% alcohol and air dried. During the test phase, all eight arms were accessible and peanut pellets were placed in the previously blocked four arms. Mice were given 5 min to search for food. The number of errors and time required to find all pellets were recorded. The experiment was conducted continuously over eight consecutive days.

Barnes maze (BM). The Barnes maze consisted of a circular platform, 75 cm in diameter, with 18 equidistant apertures (each 5 cm in diameter) around its perimeter, one leading to an escape box below. The maze was surrounded by spatial cues of different shapes and colors. The test was conducted under bright lighting to prompt the mouse to search for the escape box. During the training phase, the experimental mouse was first confined to the central region with a transparent chamber for 20 s before being instructed to explore and locate the escape box. Successful training was defined as all four limbs of the mouse entering the escape chamber within 3 min, followed by an additional acclimation period of 1 min to the escape box. During the acquisition phase (days 1–3), mice were initially confined to the central region with an opaque box for 20 s before searching for the escape box within 3 min. The mouse was tested twice daily for three consecutive days with the escape box positioned in a fixed location, followed by the reversal acquisition phase (days 4–6), during which the box was moved to the opposite side to assess cognitive flexibility and short-term memory. Data from the two trials performed each day were averaged. For each trial, the number of primary errors, primary latency, primary path, and search strategy were analyzed. Primary errors were defined as incorrect responses (head deflection or nose poking into incorrect holes) made before the first encounter with the escape chamber. Primary latency (or primary path) referred to the time spent (or distance traveled) before the escape chamber was first located. For the JZL184 rescue experiment (1), JZL184 (5 mg/kg) was administered to NEX-het-cKO mice by intraperitoneal injection daily on days 1–6. The same amount of saline was administered to the control and NEX-het-cKO mice accordingly.

Gait analysis. A 50 cm long and 10 cm wide tunnel was lined with a piece of white paper of the same size, with nesting material from the home cage placed at one end. The forepaws and hindpaws of the mouse were painted with red and blue non-toxic water-based ink, respectively. The mouse was then allowed to walk through the tunnel, leaving its footprints on the white paper. Parameters measured included stride length, stride irregularity, stride width (front base or hind base), overlap, and overlap irregularity. Stride length was defined as the average distance between consecutive hind footprints. Stride irregularity referred to the coefficient of variation (CV) of stride length. Stride width was the average distance between the left and right forepaws or hindpaws. Overlap was defined as distance between the forepaw and hindpaw footprints on the

same side, the CV of which was calculated as overlap irregularity.

Three-chamber social test. The three-chamber social test was conducted in a three-chamber box over three sessions. The apparatus consisted of three chambers (30 cm × 30 cm × 30 cm) with connecting doors. Two inverted polycarbonate cylinders with a circular base radius of 5 cm and a height of 15 cm were placed in the center of the side chambers. During the habituation session, the test mouse was first placed in the central chamber and allowed to freely explore all three chambers for 10 min. In the subsequent sociability session, an age-matched male C57BL/6 mouse (stranger 1) was placed in the cylinder on one side chamber, while the other cylinder on the opposite side was left empty. The subject mouse was allowed to freely explore the two cylinders (with or without stranger 1) for 10 min. Sniffing time was defined as the duration during which the subject mouse entered a 10 cm radius zone centered each cylinder. The social index was calculated by: social index = $(T_{stranger} - T_{empty}) / (T_{stranger} + T_{empty})$, where $T_{stranger}$ and T_{empty} represent sniffing time directed towards the two cylinders, respectively. Finally, during the social novelty session, another stranger mouse (stranger 2) was placed into the empty cylinder. The subject mouse was given another 10 min to freely explore the two cylinders (with stranger 1 or stranger 2). Sniffing time directed towards the familiar mouse (S1) and novel mouse (S2) was recorded. The social discrimination index (SDI) was calculated by: discrimination index = $(T_{novel} - T_{familiar}) / (T_{novel} + T_{familiar})$.

Grooming and rearing test. The test mouse was placed in a transparent cage lined with a layer of fresh bedding (< 1 cm thick) and allowed to freely explore for 20 min. Spontaneous grooming and rearing behaviors of the mouse during the last 10 min were analyzed.

Marble burying test. The marble burying test was conducted in a cage lined with fresh bedding material (~5 cm thick). A total of 20 glass marbles, each measuring 15 mm in diameter, were arranged in a grid pattern (5 × 4) on the bedding. The subject mouse was placed in the cage for 30 min to examine marble burying behavior. The number of marbles covered with bedding material (> 75%) was scored.

Novel object recognition (NOR) test. The novel object recognition test consisted of two phases. During the training phase, a pair of identical objects was placed equidistant from the wall in a square chamber and the test mouse was allowed to freely explore for 10 min. One hour after training, during the subsequent test phase, one of the objects was replaced by a novel one. The time spent sniffing each object was measured during a total sniffing time of 20 s. The discrimination index was calculated by: $(T_{novel} - T_{familiar}) / (T_{novel} + T_{familiar})$, where T_{novel} and $T_{familiar}$ represent time spent exploring novel and familiar objects, respectively.

Tube test. The tube test was conducted using a transparent Plexiglas tube (30 cm long, inner diameter of 3 cm), which provided limited space for unidirectional traversal by only one adult mouse, precluding the possibility of reversal. During the training period, mice were trained to traverse through the tube from alternating ends. Each mouse underwent eight trials per day for two consecutive days. After training, two mice from separate groups were randomly placed at opposite ends of the tube and released simultaneously to ensure that they met in the middle of the tube. The mouse that retreated from the tube first was considered the loser, while the other mouse was considered the winner, in a best of three trials. Chi-squared test was used to determine whether there was a significant difference in the chance of winning between two groups.

Grip strength. Grip strength was quantified using a digital force gauge. Each mouse was allowed to grasp a horizontal bar of the gauge with its forepaws, while a steadily increasing backward force was applied in parallel until the mouse released its grip. The mouse underwent six consecutive trials with 10-s intervals between each trial. The final score for each mouse was calculated as the mean of peak values, excluding the highest and lowest values.

Immunohistochemistry

Mice (P45–P60) were anesthetized with isoflurane and perfused transcardially with 0.01 M phosphate-buffered saline (PBS) followed by 4% paraformaldehyde (PFA, in 0.01 M PBS, pH 7.4). Brains were removed and post-fixed in 4% PFA for 4 h at 4 °C before dehydration in 30% sucrose solution (in 0.01 M PBS). Coronal slices (30 μm) containing the mPFC region were then sectioned using a Leica CM1050 microtome (Leica Biosystems, Germany) and collected in a 24-well plate with PBS.

For immunohistochemical staining against vGAT and NeuN, slices were permeabilized with 0.5% Triton X-100 in PBS for 30 min, then blocked in buffer (0.5% triton X-100 and 20% goat serum in PBS) for 2 h at room temperature (RT), followed by incubation with primary antibodies against vGAT (1:500; SYSY, 131004) and NeuN (1:1,000; Abcam, ab177487) in buffer (0.3% Triton X-100 and 10% goat serum in PBS) at 4 °C overnight. Staining was visualized with secondary antibodies conjugated to Alexa-488 (1:1,000, Invitrogen, A-11073) and Alexa-546 (1:1,000, Invitrogen, A-11035) in PBS at RT for 2 h. The sections were then stained with 4',6-diamidino-2-phenylindole (DAPI, D1306, 1:10,000; Thermo Fisher Scientific, USA) for 10 min at RT. The sections were then rinsed with PBS, mounted on slides, coverslipped, and imaged using a Zeiss confocal microscope (LSM800, Germany, 63×, NA 1.4 oil lens). All acquisition parameters were kept constant between experimental conditions. On average, 2 images were acquired from layers 1, 2/3 and 5 of the mPFC across three brain sections from each animal. Image backgrounds were normalized, and immunoreactive puncta were analyzed with ImageJ software (puncta size range 0.1-4 μm^2). For each experiment, 18 images per condition were collected and analyzed. Quantitative results from two images per slice were averaged to obtain a representative value for that slice. In total, 9 slices per condition were analyzed per experiment (n = 9 slices from 3 mice for both control and NEX-het-cKO groups). The antibodies used for immunohistochemistry are listed in Supplemental Table 4.

To quantify the number of $GCaMP6m^+$ neurons in the vCA1, brains were fixed and dehydrated 4 weeks after the viral injection of AAV-CAG-DIO-GCaMP6m. Coronal slices (30 μm thick) of the ventral hippocampus were then cut as described above, stained with DAPI and imaged using a Zeiss confocal microscope (LSM800, Germany, 20× lens). The number of pyramidal neurons expressing GCaMP6m was quantified using ImageJ software.

To reveal the distribution pattern of NEX^+ neurons, the brains of NEX-Cre; LSL-H2B-GFP mice were fixed and dehydrated as described above, followed by sagittal sectioning (30 μm). Sagittal sections were stained with DAPI, then scanned at 10× magnification using an Olympus automatic slide scanner (SV120, Japan). The acquired images were further processed using ImageJ software.

Slice electrophysiology

Adult male mice (5–8 weeks old) were anesthetized with isoflurane and decapitated. Their brains were then rapidly removed and chilled in ice-cold slicing solution containing (in mM): 228 sucrose, 26 $NaHCO_3$, 1 NaH_2PO_4, 11 D-glucose, 2.5 KCl, 0.5 $CaCl_2$, and 7.0 $MgSO_4$, oxygenated with a

mixture of 95% O_2 and 5% CO_2. Coronal slices (300 μm) of the mPFC were prepared using a vibratome (VT1200S, Leica), and immediately transferred to artificial cerebrospinal fluid (ACSF) containing (in mM): 119 NaCl, 2.5 KCl, 26 $NaHCO_3$, 1 NaH_2PO_4, 11 D-glucose, 1.3 $MgSO_4$, and 2.5 $CaCl_2$, oxygenated as described above. Slices were recovered at 35 °C for 30 min and equilibrated at RT for at least one hour before recording. Slices were immobilized in a submerged chamber and perfused continuously with oxygenated ACSF at 35 °C. Neurons were visualized with an infrared-differential interference contrast (IR-DIC) microscope (BX51WI, Olympus, Japan) equipped with a water immersion objective (40×, NA 0.80). Whole-cell patch-clamp recordings were performed using a MultiClamp 700B amplifier (Molecular Devices) and Digidata 1550B4 digitizer (Molecular Devices). Patch pipette (borosilicate glass, Sutter) resistance was 4–6 MΩ. Signals were sampled at a frequency of 20 kHz, filtered at 10 kHz, and analyzed using Clampfit v10.6 (Molecular Devices) and MATLAB (Mathworks) software. Neurons with an access resistance (Ra) > 20 MΩ or a 30% increase in Ra were excluded from further analyses. The chemicals used for slice electrophysiology are listed in Supplemental Table 4.

To record excitatory postsynaptic currents (EPSCs), layer 5 (L5) pyramidal neurons in the mPFC were recorded with an internal solution containing (in mM): 140 $CsMeSO_3$, 8 CsCl, 10 HEPES, 0.25 EGTA, 2 Mg-ATP, 0.3 Na_3-GTP, and 7 phosphocreatine (pH 7.25–7.3; osmolarity 295–300). Miniature EPSCs (mEPSCs) were recorded with tetrodotoxin (TTX, 1 μM), picrotoxin (PTX, 50 μM), and D-(-)-2-amino-5-phosphonopentanoic acid (D-AP5, 50 μM) in ACSF. To record E/I ratios, neurons were voltage-clamped at the reversal potential of EPSCs and IPSCs, respectively. Liquid junction potential (15.3 mV) was not corrected.

To record evoked EPSCs, a bipolar tungsten stimulating electrode (~150 μm apart between two tips) was positioned in layer 1 of the mPFC on the surface of the slices (~200 μm lateral from the recorded neuron). To prevent firing of the recorded neurons, 5 mM QX314 was included in the internal solution. AMPAR-mediated EPSCs were recorded at a holding potential of −70 mV. NMDAR-mediated EPSCs were recorded at a holding potential of 40 mV with 50 μM PTX and 6-cyano-7-nitroquinoxaline-2,3-dione disodium (CNQX, 20 μM) in ACSF. To obtain the input-output (I-O) relationship, extracellular stimuli were applied at increasing intensities. To record EPSC PPRs, two consecutive pulses (0.05 ms each) were delivered, separated by varying interstimulus intervals (20, 50, 100, 200, 500 ms). To test whether JZL184 or URB597 could correct the reduced EPSC PPRs observed in NEX-het-cKO mice, JZL184 (5 μM) or URB597 (1 μM) was applied to both control and NEX-het-cKO slices for at least 10 min prior to recordings. To confirm the change in presynaptic Pr, (+)-MK-801 (40 μM) was added to ACSF to block NMDAR-mediated currents in a use-dependent manner.

To evoke depolarization-induced suppression of excitation (DSE), the recorded neurons were depolarized to 0 mV for 5 s. The amplitude of the eEPSCs was compared before and after DSE induction. Three trials were recorded for each neuron and averaged. To induce S-DHPG-mediated eCB-LTD, S-DHPG (50 μM) was applied to the bath solution for 10 min following the baseline period. To test the dependence of eCB-LTD on CB1Rs, AM251 (10 μM) was added to the bath for the entire duration of the experiment. To test the modulatory effects of CB1Rs on EPSCs or IPSCs, the CB1R agonist WIN (2 μM) or CB1R antagonist AM251 (5 μM) was included in the ACSF. The eEPSCs and PPRs were recorded during the whole period.

To record inhibitory postsynaptic currents (IPSCs), L5 pyramidal neurons were recorded with an internal solution containing (in mM): 75 CsCl, 68 K-gluconate, 10 HEPES, 0.25 EGTA, 2 Mg-

ATP, 0.3 Na_3-GTP, 0.1 spermine, and 7 phosphocreatine (pH 7.25–7.3; osmolarity 295–300). Miniature IPSCs (mIPSCs) were recorded with 1 μM TTX, 20 μM CNQX, and 50 μM D-AP5 in ACSF.

Fiber photometry recording

A fiber photometry system (ThinkerTech, China) was used to record fluorescent signals. Briefly, blue LED light (470 nm, ~20 μW) was reflected by a dichroic mirror (MD498, Thorlabs) and coupled to a multimode fiber (200 μm / NA 0.37, Inper, China). Fluorescent signals were band-pass filtered (MF525-39, Thorlabs) and collected by a photomultiplier tube (model H10721-210, Hamamatsu, Japan). To record fluorescent signals in brain slices, an optical fiber was positioned ~150 μm above the L5 of the mPFC slice or the CA1 region of a dorsal hippocampal slice, specifically targeting the neurons with abundant expression of fluorescent proteins. A bipolar tungsten stimulating electrode was placed in layer 1 of the mPFC slice (~180 μm lateral to the fiber tip) or the stratum radiatum of the dCA1 (~120 μm lateral to the fiber tip). Photometric data were acquired using FiberPhotometry v3.0 software (ThinkerTech, China) and analyzed using MATLAB. Data obtained from three slices per mouse were averaged for statistical analysis.

To record GCaMP signals in axonal fibers originating from vCA1 neurons, AAV-CAG-DIO-GCaMP6m was injected bilaterally into the vCA1 of NEX-het-cKO or NEX-Cre (control) mice. Four weeks after stereotaxic injection, mPFC slices were prepared for fiber photometry recordings. Trains of electrical pulses (200 μA, 0.2 ms; 5, 10, 20, or 50 pulses at 50 Hz) were delivered at 60-s intervals. To test whether JZL184 could block the enhanced Ca^{2+} transients observed in NEX-het-cKO mice, JZL184 (5 μM) was applied to both control and NEX-het-cKO slices for at least 10 min before GCaMP signals were recorded.

To measure eCB release, AAV-EF1α-DIO-eCB2.0 was injected bilaterally into the mPFC of NEX-het-cKO or NEX-Cre (control) mice. Trains of electrical pulses (25, 50, 100, or 150 μA; 0.1 ms, 15 pulses at 50 Hz) were delivered to evoke eCB release at 200-s intervals. The $GRAB_{eCB2.0}$ signals were quantified and normalized to the baseline level. AM251 (5 μM) was added to ACSF to confirm the specificity of the eCB sensors. Puff application of 2-AG (200 μM) or AEA (100 μM) was used to test the specificity of the eCB sensors. To test the contribution of 2-AG release to $GRAB_{eCB2.0}$ signals, electrical pulses (200 μA, 0.5 ms, 50 pulses at 50 Hz) were delivered every 150 s. The DAGL inhibitor DO34 (1 μM) was added to the ACSF after a 10-min baseline recording. To directly measure 2-AG or AEA release, AAV-Syn-DIO-2-AG1.2 or AAV-CaMKII-AEA1.2 was injected bilaterally into the mPFC or CA1. A series of electrical pulses of varying intensity (50, 100, 150, or 200 μA; 0.1 ms, 25 pulses at 50 Hz) were delivered to the mPFC slices at 200 s intervals. Puff application of 2-AG (400 μM) or AEA (100 μM) was used to test the specificity of the two sensors.

Morphological reconstruction and analysis

L5 pyramidal neurons in the mPFC were recorded for 10–15 min with Cs-methanesulfonate-based internal solution containing 0.2% biocytin (B4261, Sigma). The recording pipette was then carefully withdrawn to preserve cell membrane integrity. The slices were fixed in 4% PFA overnight at 4 °C, then washed three times in 0.01 M PBS, permeabilized with 0.5% Triton X-100 for 1 h at RT and incubated in Alexa Fluor 488-conjugated streptavidin (S11223, 1:1,000, Thermo Fisher Scientific) for 2 h at RT. Z-stack images (0.75 μm/image) were acquired using a LSM 800 confocal microscope (Zeiss, 20× objective, NA 0.8). The three-dimensional (3D) reconstruction

of dendritic trees was performed using ImageJ software (NIH, Bethesda, Maryland, USA). Dendritic complexity was calculated through Sholl analysis. To analyze dendritic spines, z-stack images were acquired at 0.5 μm intervals using the same confocal microscope (Zeiss, 63× oil objective, NA 1.4) with 2 × digital zoom. To assess spine density along distal apical, proximal apical, and basal dendrites, three dendritic segments were analyzed per neuron, and the average spine density was used for subsequent analyses.

***In vitro* biochemistry studies**

Cell culture. The N2a cell line (CL-0168) was purchased from Procell Life Science & Technology Co., Ltd. The cells were confirmed to be free of mycoplasma contamination. The N2a cells were cultured in 6-well plates using 1 × basal Dulbecco's Modified Eagle Medium (DMEM) (Gibco, USA) supplemented with 10% FBS (BI) and 1% penicillin-streptomycin (Gibco, USA). After 12 h of incubation to allow cell attachment and stabilization, synchronization and differentiation were induced through serum starvation by replacing the medium with DMEM containing 1% FBS and 1% penicillin-streptomycin for 24 h. Upon reaching 60–70% confluence, cells were subjected to chemical treatments or transfection in DMEM with 10% FBS and 1% penicillin-streptomycin. For chemical treatments, cells were exposed to either 10 nM okadaic acid (OA, ab141831, Abcam, USA) for 24 h or 250 nM EPZ011989 (HY-16986, MedChemExpress, USA) for one to four days. For PP2Ac overexpression, cells were transfected with either ubiquitin (Ub)-PP2Ac-IRES-EGFP or Ub-EGFP using Lipofectamine 3000 (Invitrogen). After transfection, cells were cultured for another 24 h before RNA or protein extraction.

Protein extraction, synaptosome fractionation, and western blotting. N2a cells or cortical tissues were homogenized in RIPA lysis buffer (Beyotime Biotechnology, China), supplemented with protease inhibitor cocktail (Servicebio, China) and phosphatase inhibitor (Biosharp, China). Samples were then vortexed vigorously to ensure complete dissociation, followed by centrifugation at 12,000 × *g* for 10 min at 4 °C to remove debris. The supernatant, containing the total proteins, was carefully transferred to a new tube, and stored until use. For protein half-life experiments, cortical tissues or N2a cells were treated with 300 μg/mL Cycloheximide (CHX, HY-12320, MedChemExpress, USA) for 0, 4, 8 or 12 hours before protein extraction.

Synaptic protein extraction reagent (Syn-PER, Thermo Fisher Scientific) was used for synaptosome isolation. Cortical tissue was homogenized in Syn-PER reagent supplemented with a protease inhibitor cocktail (Servicebio, China) and phosphatase inhibitor (Biosharp, China). The homogenate was centrifuged at 1,200 × *g* for 10 min at 4 °C. The pellet was discarded, and the supernatant (total protein) was transferred to a new tube, then centrifuged at 15,000 × *g* for 20 min at 4 °C to isolate the synaptosome (pellet) from the cytosolic (supernatant) fraction. The synaptosome pellet was resuspended in Syn-PER reagent for downstream analyses.

Protein concentration was measured using a BCA Protein Assay Kit (Elabscience). Samples were standardized to an equivalent concentration, mixed with 5 × sodium dodecyl-sulfate polyacrylamide gel electrophoresis (SDS-PAGE) protein loading buffer (Beyotime Biotechnology, China), and boiled at 98 °C for 5 min. For western blotting of GluA1, GluA2, GluN1, GluN2A, GluN2B, and PSD95 proteins, boiling was omitted to avoid the formation of high-molecular-weight aggregates.

For western blotting experiments, 20–40 μg of protein from each sample was loaded and separated by gel electrophoresis (One-Step PAGE Gel Fast Preparation Kit, 8%–15%, Vazyme, China).

Proteins were then transferred from the gels to an Immobilon-P membrane (0.2 μm, Merck, USA). The membrane was treated with 5% non-fat dry milk in TBST at RT for 1 h, then incubated with primary antibodies in Western Antibody Dilution Buffer (Elabscience) overnight at 4 ℃. The membrane was washed three times with TBST and incubated with Alexa Fluor Plus 800-conjugated secondary antibodies in TBST for 1 h at RT. The following primary antibodies were used: anti-GluA1 (1:20,000, Proteintech, China), anti-GluA2 (1:1,000, Proteintech), anti-GluN1 (1:1,000, Proteintech), anti-GluN2A (1:1,000, Proteintech), anti-GluN2B (1:1,000, Proteintech), anti-PSD95 (1:2,000, Thermo Fisher Scientific), anti-PLCβ3 (1:5,000, Proteintech), anti-MAGL (1:600, Proteintech), anti-FAAH (1:1,000, Proteintech), anti-COX2 (1:1,000, Thermo Fisher Scientific), anti-PPP2R1A (1:1,000, Proteintech), anti-CB1R (1:1,000, Proteintech), anti-NAPE-PLD (1:200, Abcam), anti-NAAA (1:1,000, Santa Cruz), anti-EZH2 (1:5,000, Proteintech), anti-EZH2 (1:1,000, Abcam), anti-β-Actin (1:5,000, Proteintech), anti-β-tubulin (1:2,000, Thermo Fisher Scientific), anti-GAPDH (1:5,000, Proteintech), and anti-PP2A-Cα (1:1,000, Immunoway, USA). Proteins were visualized using goat anti-mouse Alexa Fluor Plus 800 (1:40,000, Thermo Fisher Scientific), goat anti-rabbit Alexa Fluor Plus 800 (1:40,000, Thermo Fisher Scientific), and goat anti-guinea pig DyLight 800 secondary antibodies (1:20,000, Thermo Fisher Scientific). Total protein bands were stained using a Revert 700 Total Protein Staining Kit (926-11010, LI-COR, USA). The Odyssey CLx infrared imaging system (LI-COR) was used to acquire images of protein bands. Band intensity of each sample was calculated using Image Studio v5.2 software, divided by the intensity of the loading control protein (β-Actin, β-Tubulin, GAPDH, or total protein) on the same blot. The antibodies used for western blotting are listed in Supplemental Table 4.

RNA isolation and quantitative reverse transcription polymerase chain reaction (RT-qPCR). Total RNA was extracted from brain tissue or N2a cells using Trizol reagent (Ambion, USA). Reverse transcription was performed with 1 μg of total RNA using HiScript®III RT SuperMix (Vazyme, Nanjing, China). RT-qPCR was performed using the LightCycler 96 Real-Time PCR System (Roche, Switzerland) with ChamQ SYBR qPCR Master Mix (Vazyme, Nanjing, China). Melting curve analysis was performed to validate the specificity of the amplification. Transcription levels were normalized to the internal control. The experiments were performed in triplicate. The primers used included: *β-Actin*-F 5′-GGCTGTATTCCCCTCCATCG-3′; *β-Actin*-R 5′-CCAGTTGGTAACAATGCCATGT-3′; *Magl*-F 5′-CGGACTTCCAAGTTTTTGTCAGA-3′; *Magl*-R 5′-GCAGCCACTAGGATGGAGATG-3′; *Faah*-F 5′-GAGGCTCCCCTCTGGGTTTA-3′; and Faah-R 5′-GCCAGGCTATCCACATCCC-3′.

Fluorescence-activated cell sorting (FACS)

Three weeks after AAV-Ef1α-DIO-EYFP injection into the mPFC of NEX-het-cKO or NEX-Cre (control) mice, coronal slices were prepared using a vibratome as described above. The mPFC region was dissected and digested with papain dissociation solution containing 0.1 mg/mL papain (Worthington, USA), 1 mM $CaCl_2$, and 0.5 mM EDTA in Hank's Balanced Salt Solution (HBSS) for 30 min at 37 ℃. The brain tissue was rinsed with ice-cold DMEM (Gibco) containing 10% fetal bovine serum (FBS, BI, USA) to terminate digestion, then resuspended in oxygenated slicing solution, followed by gentle trituration with pipette tips until a single-cell suspension was achieved. The resulting suspension was then filtered through a 70-μm nylon cell strainer (Biosharp, China) to remove debris and stained with DAPI (1:10,000, Thermo Fisher Scientific) on ice in the dark. Intact $EYFP^+$ neurons were sorted using a BD FacsAria III machine (BD Biosciences, USA).

Sorted RNA sequencing

Total RNA of EYFP$^+$ neurons was extracted using an Arcturus PicoPure RNA Isolation Kit (Thermo Fisher Scientific, USA). The quality, purity, and quantity of RNA samples were evaluated with the Agilent RNA 6000 Pico Kit (Agilent Technologies, USA) on the Agilent 2100 Bioanalyzer System (Eukaryote Total RNA Pico Series II, Agilent Technologies, Inc.). Samples with a minimum RNA Integrity Number (RIN) ≥ 7 and RNA concentration of at least 100 pg/μL were included for further experiments. After fragmentation, RNA samples were used for synthesis of double-stranded cDNA, which was indexed using a SMARTer Stranded Total RNA-Seq Kit v2-Pico Input Mammalian (Takara Bio, Japan) and purified using Agencourt AMPure XP beads (Beckman Coulter). Deletion of ribosomal cDNA using ZapR Master Mix and the second PCR amplification were then performed. Finally, library quantification was carried out using a Qubit dsDNA HS Kit (Thermo Fisher Scientific), and size distribution analysis was conducted using an Agilent High Sensitivity DNA Kit (Agilent Technologies, Inc.). The cDNA libraries typically displayed a local maximum within the range 300–500 bp. Eligible libraries were sequenced using a NovaSeq 6000 sequencer (Illumina, USA).

Quantitation, identification, and annotation of DEGs

As described in previous study (2), high-quality sequencing data were obtained by removing reads containing adapters or poly-N regions and low-quality reads from the raw data using Cutadapt v3.7. All subsequent analyses were performed based on the trimmed reads. The reads were then aligned to the GRCm38 *Mus musculus* reference genome (Ensembl release) using HISAT2 v2.0.5. and accurately quantified using FeatureCounts v1.5.0-p3, a powerful tool for RNA-seq quantification. The resulting data were imported into R software for DEG analysis using the DESeq2 R package (3). DEGs were identified using a threshold of $|\log_2(\text{fold-change})| > 1$ and P.adj < 0.05 for sorted RNA-Seq data. Sample quality was assessed by calculating distances between each other using the ***dist*** function. Principal component analysis (PCA) was performed using only two principal component variables. To validate their reliability, DEGs of interest were visualized as a normalized expression heatmap using hierarchical clustering. Gene Ontology (GO) and Kyoto Encyclopedia of Genes and Genomes (KEGG) enrichment analyses were performed using the clusterProfiler package to elucidate the functions and pathways associated with the DEGs (4).

TRFs motif enrichment analyses of the DEGs

Using pre-calculated whole-genome motif rankings and the recovery approach (5), we employed the R package RcisTarget to identify potential transcription-regulatory factor (TRF) binding motifs from DEGs identified in sorted RNA-Seq analyses. Briefly, we conducted TRF motif enrichment analyses based on genome-wide rankings for each motif, utilizing the mm10_refseq-r80_10 kb_up_and_down_tss.mc9nr dataset to identify motifs within 10 kb of the promoter region of DEGs and calculated the area under the curve (AUC) of the cumulative recovery curve in each motif gene set as a metric to quantify the enrichment of these regions. By combining the distribution of the AUC and the normalized enrichment score (NES) [NES = (AUC − AUC_mean) / AUC_std)], we determined the top enriched motifs with AUC value > mean + 2SD and NES > 3. The enriched motifs were followed by annotation of potential TRFs targeting them. In addition, we searched the BioGRID database (6) (https://thebiogrid.org/) and obtained a list of proteins that could physically interact with PPP2R1A based on the experimental evidence from co-

immunoprecipitation and mass spectrum, and then screened for reliable TRFs that were directly regulated by PPP2R1A protein. Finally, the network of PPP2R1A-TRFs-DEGs with motif information was visualized using Cytoscape 3.9.0 (7)

TRFs overrepresentation analyses

The ENCODE_TF_ChIP-seq_2015 gene set library was obtained from the Enrichr website (https://maayanlab.cloud/Enrichr/#libraries), which consists of 816 gene sets derived from 816 independent ChIP-seq experiments of 181 TRFs in the ENCODE project (8). The DEGs identified from sorted RNA-Seq data sets were subjected to over-representation analysis (ORA) using the 816 gene sets, with a significance threshold of $P < 0.05$. ORA assays were performed in R software using a one-tailed Fisher's exact test with a 95% confidence level, as calculated by the R function "fisher.test", and a Bonferroni correction was applied for multiple comparisons. The total number of human protein-coding genes was determined to be 20,438 based on the GRCh38 assembly in the analysis.

Chip-Seq analyses

Seven EZH2 Chip-Seq datasets were retrieved from the ENCODE and GEO databases, covering data from four different cells across two species. The merged bigWig files of irreproducibility discovery rate (IDR) thresholded peaks were obtained directly from the ENCODE database (https://www.encodeproject.org/) (8), including ENCSR886KKK (human neurons, EZH2phosphoT487 Chip-seq), ENCSR656MXA (human neural progenitor cells, EZH2phosphoT487 Chip-seq), and ENCSR069DPL (human neural progenitor cells, EZH2 Chip-seq). The original sequencing files from the two datasets were downloaded from GEO database, including GSE89929 (EZH2 Chip-seq of mouse neural progenitor cells: GSM2393590, GSM1917394 and GSM1917395; EZH2 Chip-seq of mouse embryonic stem cells: GSM2393584, GSM1917392 and GSM1917393) (9) and GSE74330 (EZH2 Chip-seq of mouse neural progenitor cells: GSM1917302 and GSM1917305; EZH2 Chip-seq of mouse embryonic stem cells: GSM1917297 and GSM1917300) (10). The original FASTQ files were processed and analyzed using the same pipeline in above ENCODE Chip-Seq analysis, based on the GRCm38 *Mus musculus* reference genome. Peaks were called for each sample, and the final peak sets were generated based on the optimal set calculated by the SPP peak caller and IDR thresholded peaks (IDR < 0.05) after subtracting the input signal. The peaks were all visualized using the IGV tool. All ESR1 Chip-Seq datasets and three EP300 Chip-Seq datasets of neuronal cells were retrieved from the ENCODE, including ENCSR000BKL, ENCSR000BIZ, ENCSR000BIY, ENCSR000BQR, ENCSR463GOT, ENCSR000BZZ, ENCSR000BJS, ENCSR000BQD, ENCSR000BKN, ENCSR000BLL, ENCSR000EHV, ENCSR000BMA, ENCSR000BUA. Similarly, the merged bigWig files of IDR thresholded peaks were used to estimate the binding between the TRFs and MAGL.

Supplemental Figures and Figure Legends

Supplemental Figure 1

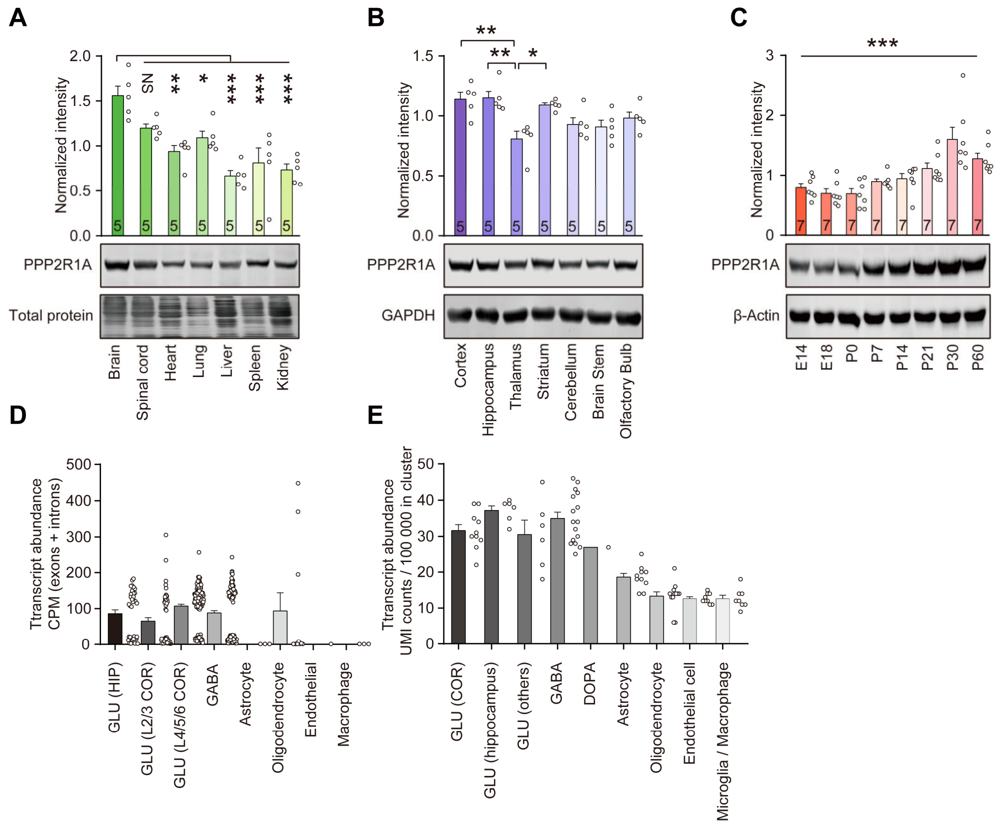

Supplemental Figure 1. Spatiotemporal expression pattern of *Ppp2r1a* in the mouse brain.

(**A**) Western blot analysis showing that PPP2R1A is most abundantly expressed in the mouse brain (n = 5 mice).

(**B**) Western blot analysis showing the regional distribution of PPP2R1A protein levels across different brain regions (n = 5 mice).

(**C**) Temporal expression pattern of PPP2R1A protein from embryonic day 18 (E18) to postnatal day 60 (P60) (n = 7 mice for each group).

(**D**) single-cell RNA-seq from the Allen Brain Institute showing *Ppp2r1a* mRNA expression levels in neurons and glial cells (11), reported in counts per million (CPM).

(**E**) Single-cell RNA-seq data from McCarroll Lab depicting *Ppp2r1a* mRNA levels in neurons and glial cells (12), expressed as unique molecular identifiers (UMI).

Statistical comparisons were performed using one-way ANOVA followed by Bonferroni *post-hoc* test (**A-C**). Abbreviations: GLU, glutamatergic neurons; GABA, GABAergic neurons; DOPA, dopaminergic neurons; HIP, hippocampus; COR, cortex. All data are presented as mean ± SEM. NS, non-significant, *$P < 0.05$, **$P < 0.01$, ***$P < 0.001$.

Supplemental Figure 2

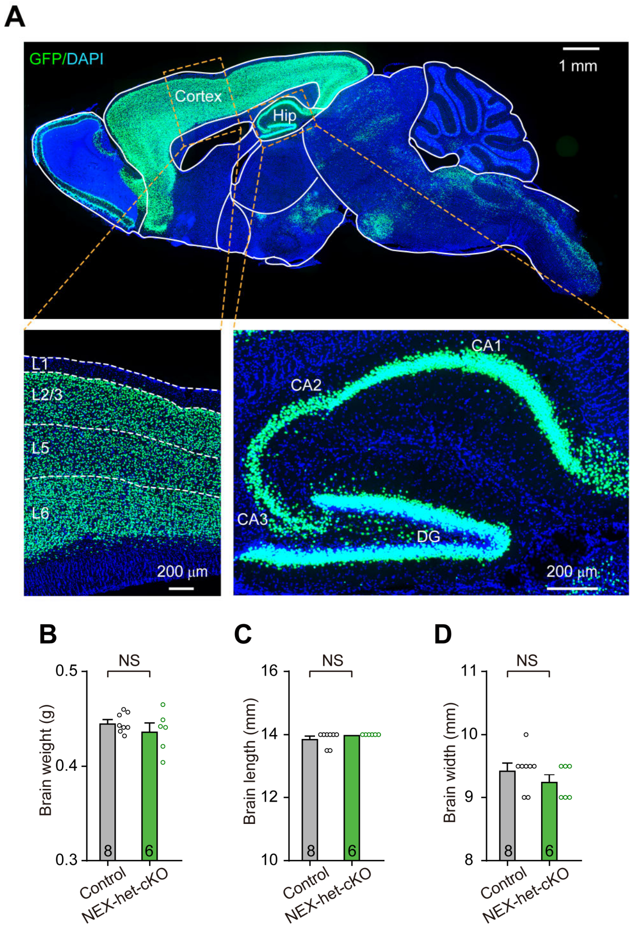

Supplemental Figure 2. Heterozygous deletion of *Ppp2r1a* in forebrain excitatory neurons do not change brain size.

(**A**) Representative sagittal section derived from NEX-Cre; LSL-H2B-GFP mouse. Top, distribution of NEX^{+} neurons in brain; bottom, higher resolution images of cortex and hippocampus. Scale bars: 1 mm (top), 200 µm (bottom).

(**B-D**) Normal brain weight, brain length and brain width in NEX-het-cKO mice, respectively (Control, n = 8 mice; NEX-het-cKO, n = 6 mice).

Statistical comparisons were performed using two-tailed unpaired Student's *t*-test (**B**), two-tailed Mann-Whitney test (**C**, **D**). All data are presented as mean ± SEM. NS, non-significant.

Supplemental Figure 3

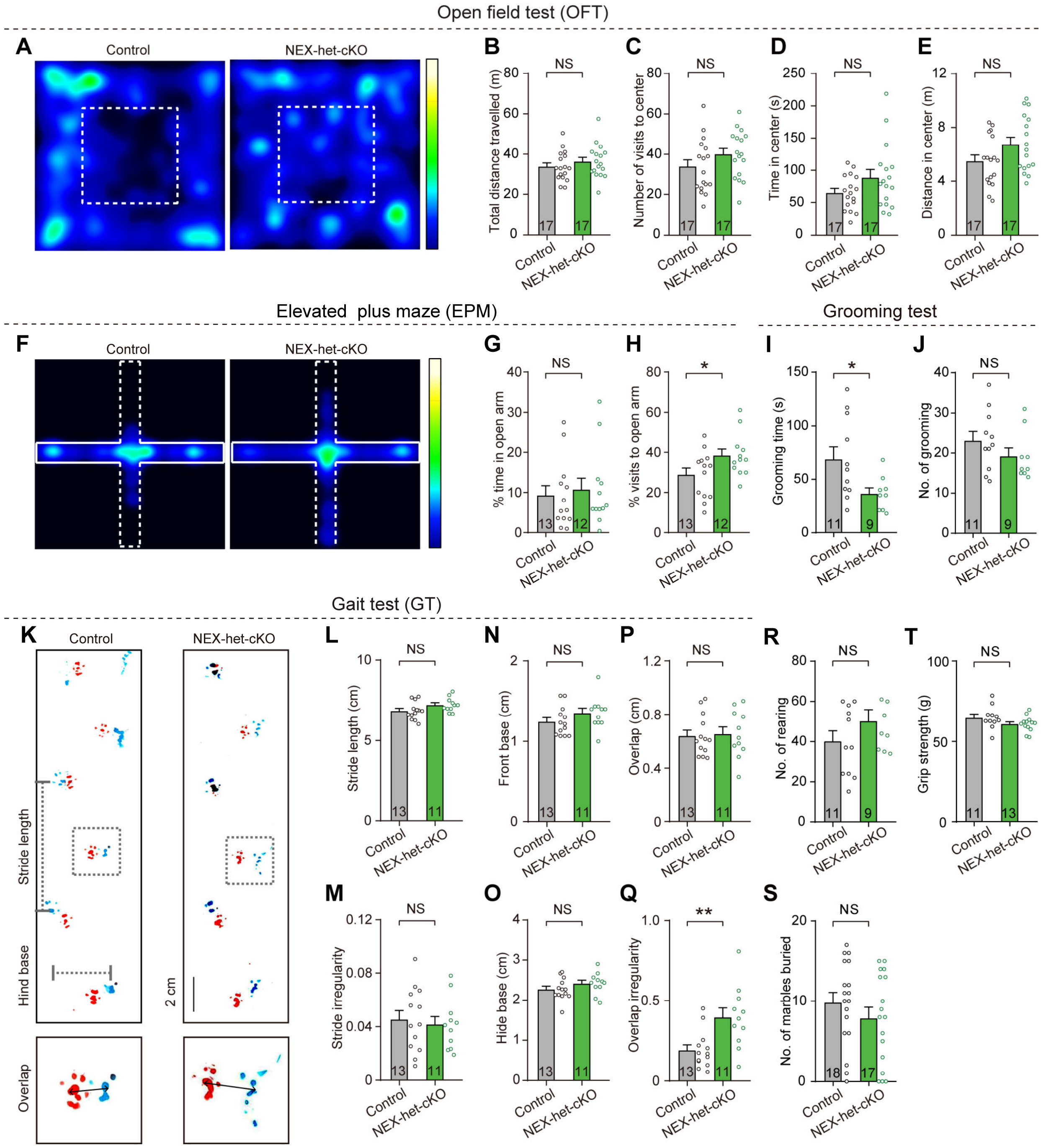

Supplemental Figure 3. NEX-het-cKO mice exhibit reduced anxiety levels and mild motor coordination.

(**A-E**) NEX-het-cKO mice showed normal performance in the OFT (Control, n = 17; NEX-het-cKO, n = 17). (**A**) Representative heatmap. (**B**) Total distance traveled. (**C**) Number of visits to center. (**D**) Time in center. (**E**) Distance in center.

(**F-H**) NEX-het-cKO mice exhibited reduced anxiety-like behavior in the EPM (Control, n = 13; NEX-het-cKO, n = 12). (**F**) Representative heatmap. Percentage of time (**G**) and visits (**H**) in open arms.

(**I-J**) Reduced grooming time (**I**) but normal grooming bouts (**J**) in NEX-het-cKO mice (Control, n = 11 mice; NEX-het-cKO, n = 9 mice).

(**K-Q**) Gait test (GT) showed increased overlap irregularity in NEX-het-cKO mice (Control, n = 13 mice; NEX-het-cKO, n = 11 mice). (**K**) Representative images from footprint analyses. (**L-Q**) Group data of stride length (**L**), stride irregularity (**M**), front base (**N**), hind base (**O**), overlap (**P**), and overlap irregularity (**Q**).

(**R**) Rearing behavior was normal in NEX-het-cKO mice (Control, n = 11 mice; NEX-het-cKO, n = 9 mice).

(**S**) NEX-het-cKO mice showed normal marble burying behavior (Control, n = 18 mice; NEX-het-cKO, n = 17 mice).

(**T**) Grip strength was unaffected in NEX-het-cKO mice (Control, n = 11 mice; NEX-het-cKO, n = 13 mice).

Statistical comparisons were performed using two-tailed unpaired Student's *t*-test (**B-C**, **E**, **H**, **I**, **R-T**, **L-Q**) and two-tailed Mann-Whitney test (**D**, **G**, **J**). All data are presented as mean ± SEM. NS, non-significant, $^{*}P < 0.05$, $^{**}P < 0.01$.

Supplemental Figure 4

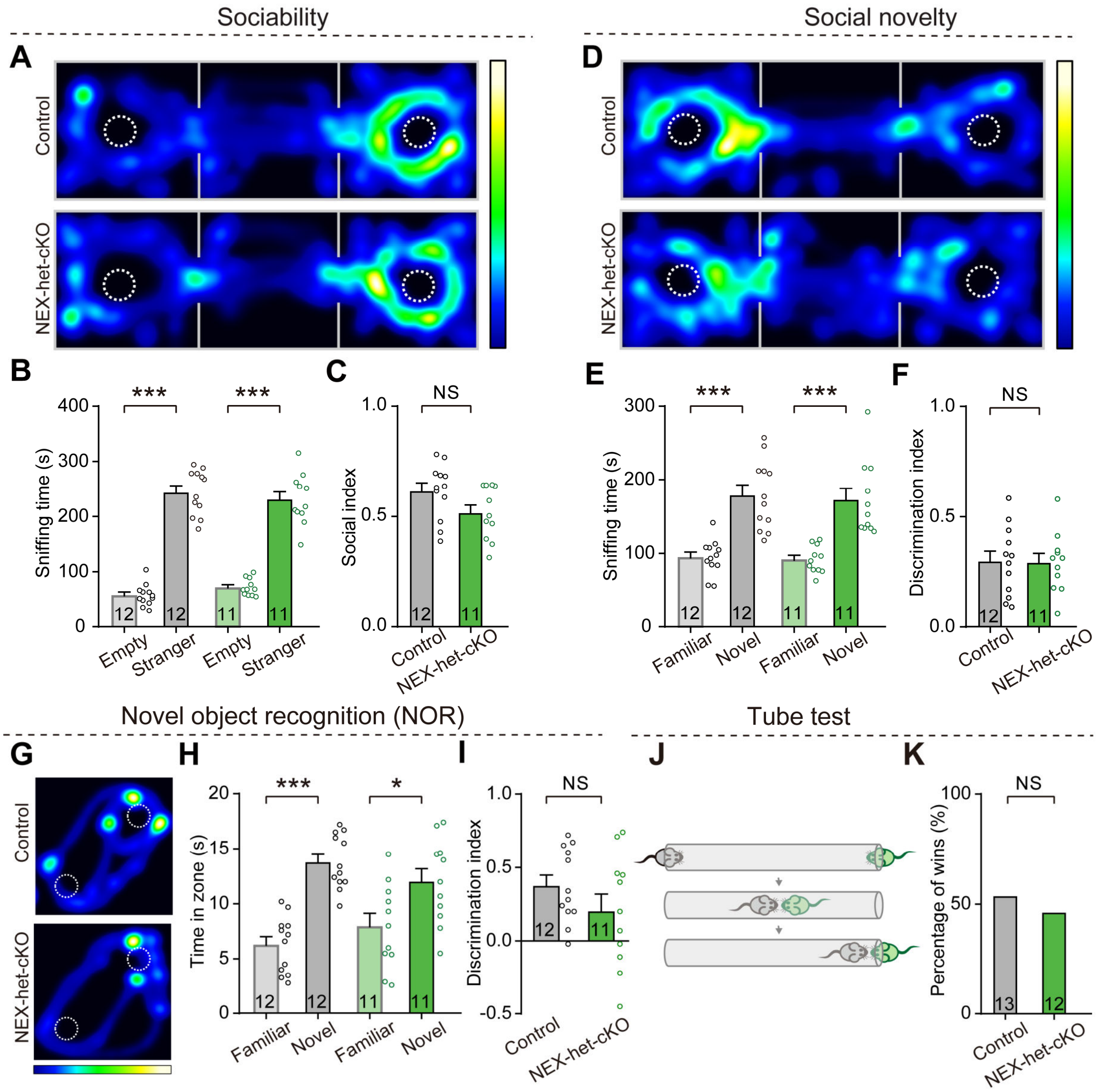

Supplemental Figure 4. NEX-het-cKO mice exhibit normal social behavior and social hierarchy.

(**A-F**) NEX-het-cKO mice showed normal sociability (**A-C**) and social novelty preference (**D-F**) in three-chamber social test (Control, n = 12 mice; NEX-het-cKO, n = 11 mice).

(**G-I**) NEX-het-cKO mice showed a normal preference for the novel object (Control, n = 12 mice; NEX-het-cKO, n = 11 mice). (**G**) Representative track map of control and NEX-het-cKO mice. (**H**) Interaction time with the familiar and the novel objects. (**I**) Discrimination index.

(**J-K**) Percentage of wins in paired trials between control and NEX-het-cKO mice showed no difference in social hierarchy (Control, n = 13 mice; NEX-het-cKO, n = 12 mice). (**J**) Schematic of tube test. (**K**) Percentage of wins. Note that control mice won 43 out of 80 trials, while NEX-het-cKO mice won the remaining trials.

Statistical comparisons were performed using two-tailed unpaired Student's *t*-test (**B-C**, **E-F**, **H-I**) and Chi-square tests (**K**). All data are presented as mean ± SEM. NS, non-significant, $*P < 0.05$, $***P < 0.001$.

Supplemental Figure 5

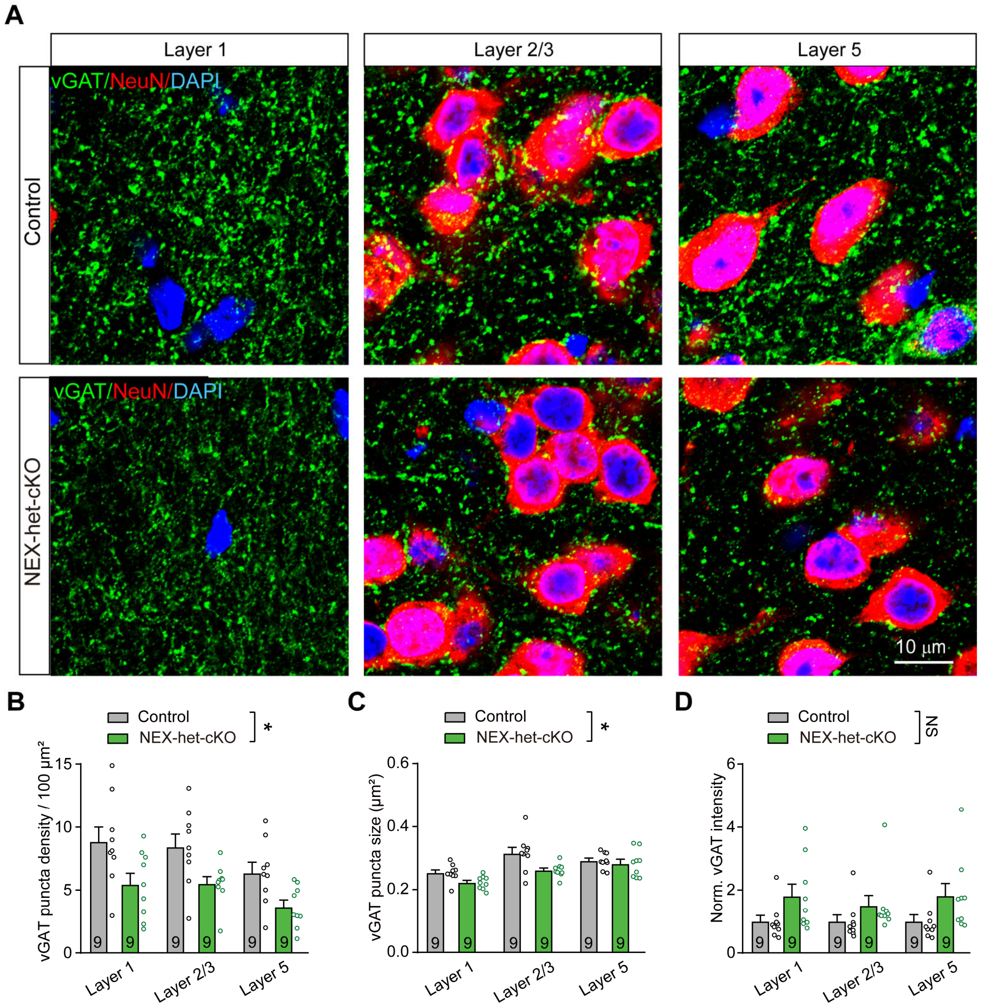

Supplemental Figure 5. *Ppp2r1a* haploinsufficiency reduces the number of inhibitory synapses.

(**A**) Representative images of layer 1, layer 2/3 and layer 5 of mPFC sections immunostained for vGAT (green), NeuN (red), and DAPI (blue).

(**B-D**) The number of inhibitory synapses was reduced in NEX-het-cKO mice (Control, n = 9 slices from 3 mice; NEX-het-cKO, n = 9 slices from 3 mice). (**B**) Quantitative analysis of $vGAT^+$ puncta density showing reduced number of inhibitory synapses in NEX-het-cKO mice. (**C**) Same as panel (**B**) but for $vGAT^+$ puncta size. (**D**) Same as panel (**B**) but for fluorescence intensity of $vGAT^+$ puncta.

Statistical comparisons were performed using two-way ANOVA followed by Bonferroni test (**B-D**). All data are presented as mean ± SEM. NS, non-significant, $*P < 0.05$

Supplemental Figure 6

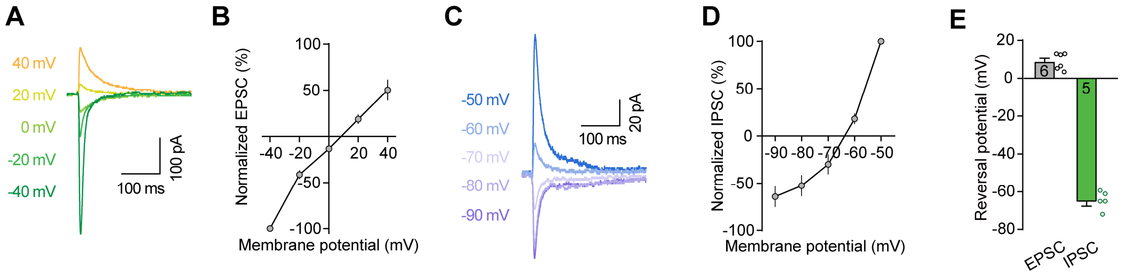

Supplemental Figure 6. Measurement of reversal potentials of EPSCs and IPSCs.

(**A**) Representative EPSC traces recorded from mPFC L5 pyramidal neurons with holding potentials (V_{hold}) ranging from −40 mV to 40 mV.

(**B**) Current-voltage (I-V) relationship of average EPSCs showing a near-linear trend and a reversal potential of 8.87 ± 1.77 mV (n = 6 neurons).

(**C**) Representative traces of IPSCs with V_{hold} ranging from −90 mV to −50 mV.

(**D**) I-V relationship of averaged IPSCs with a reversal potential of −64.49 ± 2.20 mV (n = 5 neurons).

(**E**) Summary bar graph showing reversal potentials for EPSCs and IPSCs (EPSC, n = 6 neurons; IPSC, n = 5 neurons). Note that liquid junction potential (15.3 mV) was not corrected.

Supplemental Figure 7

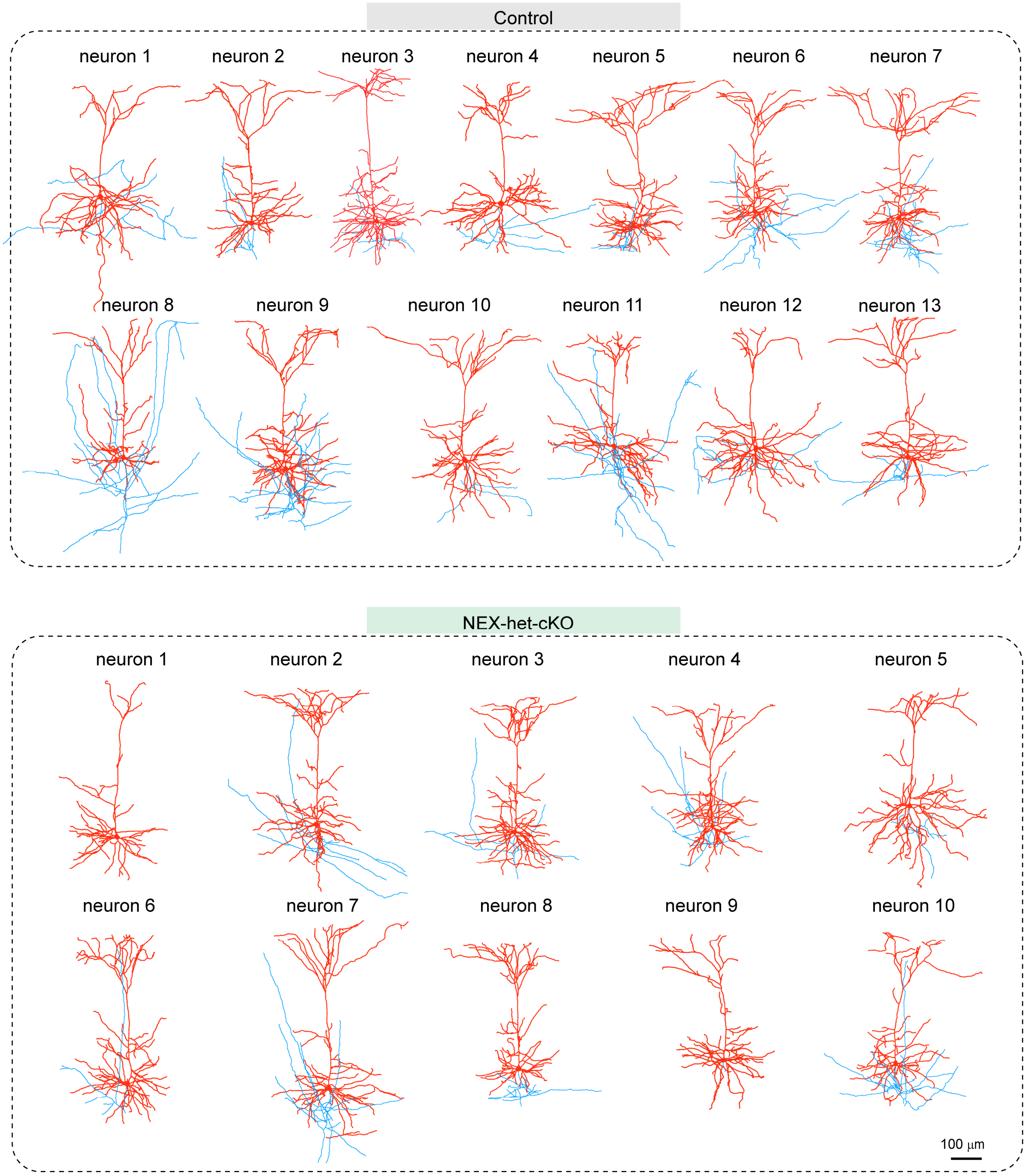

Supplemental Figure 7. Three-dimensional reconstruction of layer 5 pyramidal neurons in the mPFC.

Thirteen neurons from control mice (top) and 10 neurons from NEX-het-cKO mice (bottom) were reconstructed, respectively. Red, soma and dendrites; blue, axons.

Supplemental Figure 8

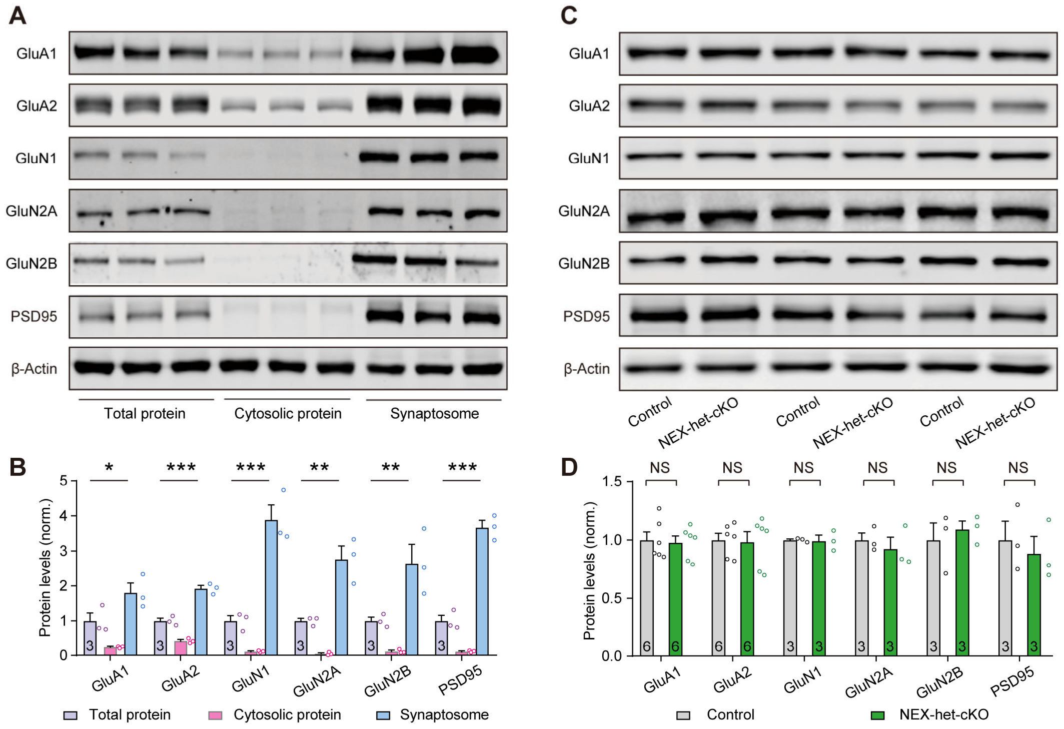

Supplemental Figure 8. Expression levels of glutamate receptors are unchanged in NEX-het-cKO mice.

(**A-B**) Western blotting analysis confirmed the enrichment of key postsynaptic proteins in the synaptosomal fraction. (**A**) Representative western blot showing increased levels of glutamate receptors and PSD95 in the synaptosomal fraction compared to cytosolic fractions. (**B**) Summary graph of protein levels in cytosolic and synaptosomal fractions compared to total protein, demonstrating significant enrichment of all synaptic proteins in synaptosomal fractions.

(**C-D**) Postsynaptic protein expression remained unchanged in the synaptosomal fraction of NEX-het-cKO mice. (**C**) Representative western blots of synaptosomes isolated from control and NEX-het-cKO mice. (**D**) Quantification showing no significant differences in postsynaptic protein levels between genotypes.

Statistical comparisons were performed using one-way ANOVA followed by Bonferroni *post-hoc* test (**B**) and two-tailed unpaired Student's *t*-test (**D**). Numbers of mice (**B**, **D**) are indicated in the graphs. All data are presented as mean ± SEM. NS, non-significant, $^{*}P < 0.05$, $^{**}P < 0.01$, $^{***}P < 0.001$.

Supplemental Figure 9

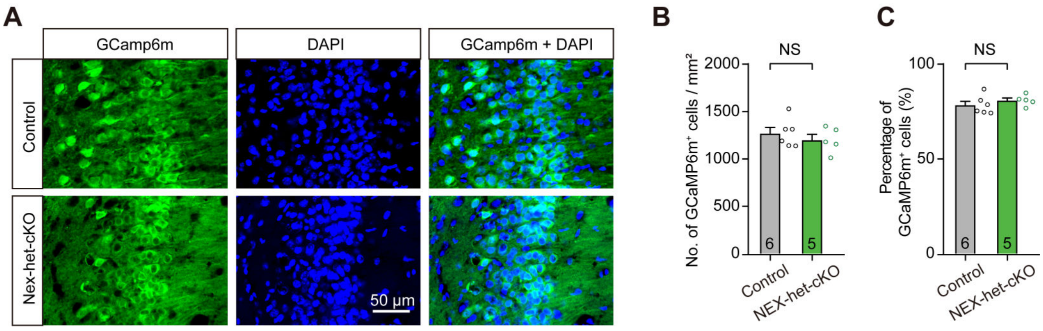

Supplemental Figure 9. Comparable GCaMP6m expression in vCA1 region across genotypes.

(**A**) Representative images showing GCaMP6m expression (green) in the vCA1 region of control and NEX-het-cKO mice. Nuclei are labeled with DAPI (blue).

(**B-C**) Quantification of the density (B) and percentage (C) of neurons expressing GCaMP6m in control (n = 6 mice) and NEX-het-cKO (n = 5 mice) groups.

Statistical comparisons were performed using two-tailed unpaired Student's *t*-test (**B-C**). All data are presented as mean ± SEM. NS, non-significant.

Supplemental Figure 10

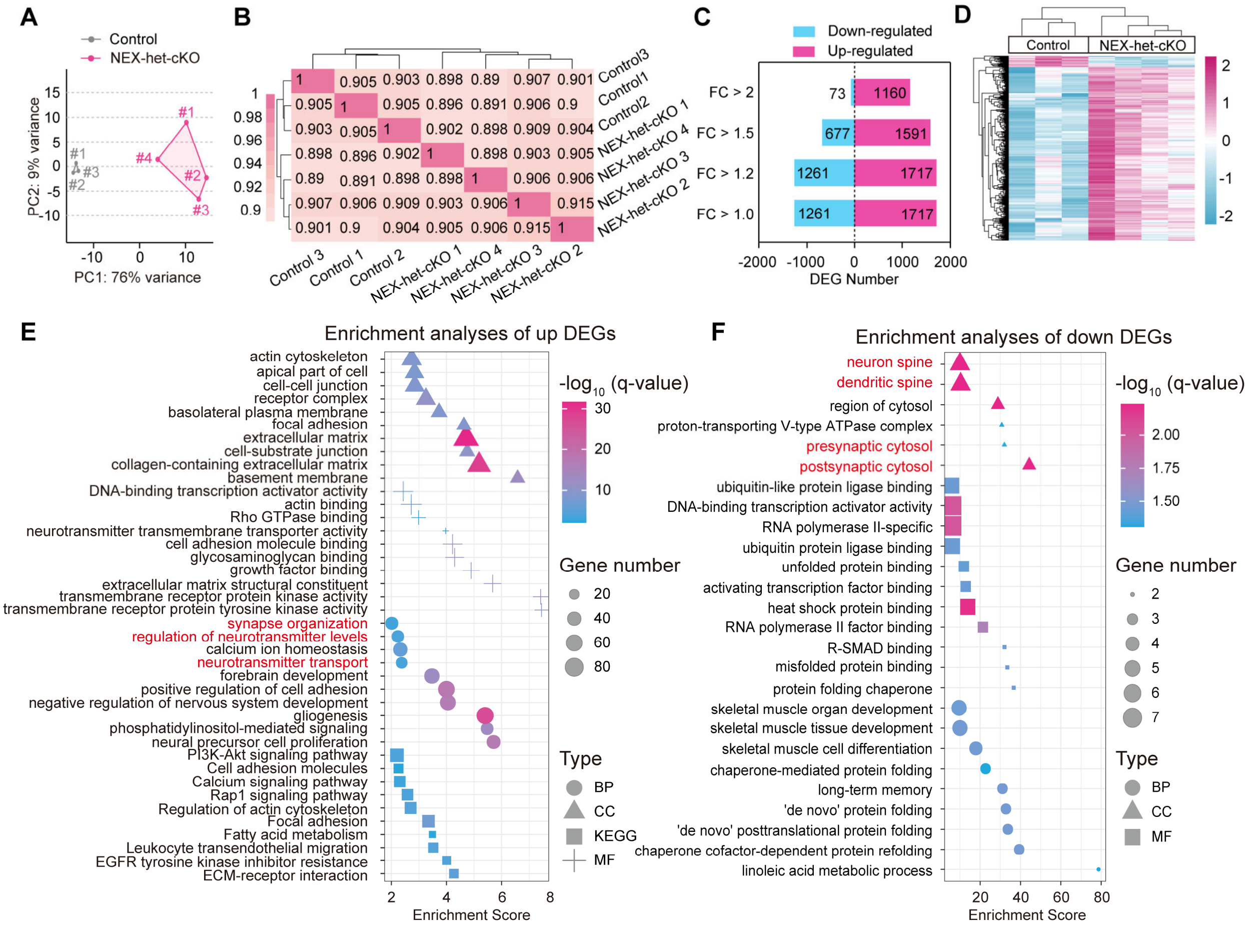

Supplemental Figure 10. Correlation and DEG analyses of sorted RNA-seq datasets.

(**A**) Principal component analysis (PCA) of sorted RNA-seq data effectively discriminated samples from control (#1-3) and NEX-het-cKO (#1-4) mice.

(**B**) Pearson correlation and hierarchical clustering of control and NEX-het-cKO replicates.

(**C**) Differential expression analysis of sorted RNA-Seq data identified 1 160 up-regulated and 73 down-regulated DEGs with FC > 2 and *P*.adj < 0.05. FC, fold change.

(**D-F**) Ppp2r1a haploinsufficiency resulted in a wide and significant change of gene expression patterns. (**D**) Heatmap and hierarchical clustering analyses of DEGs in sorted RNA-seq data. **(E)** Gene Ontology (GO) and Kyoto Encyclopedia of Genes and Genomes (KEGG) enrichment analyses based on 1 160 up-regulated DEGs identified by sorted RNA-seq. Genes with distinct functions were symbolized with diverse shapes, including MF (molecular function), BP (biological process), CC (cellular component), and KEGG (pathway). The size of symbols indicates the number of genes within each category. Biological processes related to synapse organization and release of neurotransmitter are highlighted in red. **(F)** Same as panel **(E)** but for 73 down-regulated DEGs identified by sorted RNA-seq. Cellular components associated with presynaptic/postsynaptic cytosol and spines are highlighted in red.

Statistical comparisons were performed using negative binomial distribution model of DESeq2 (**A**, **C**) (Supplemental Table 1), and one-tailed Fisher's exact test (**E-F**) (Supplemental Table 1).

Supplemental Figure 11

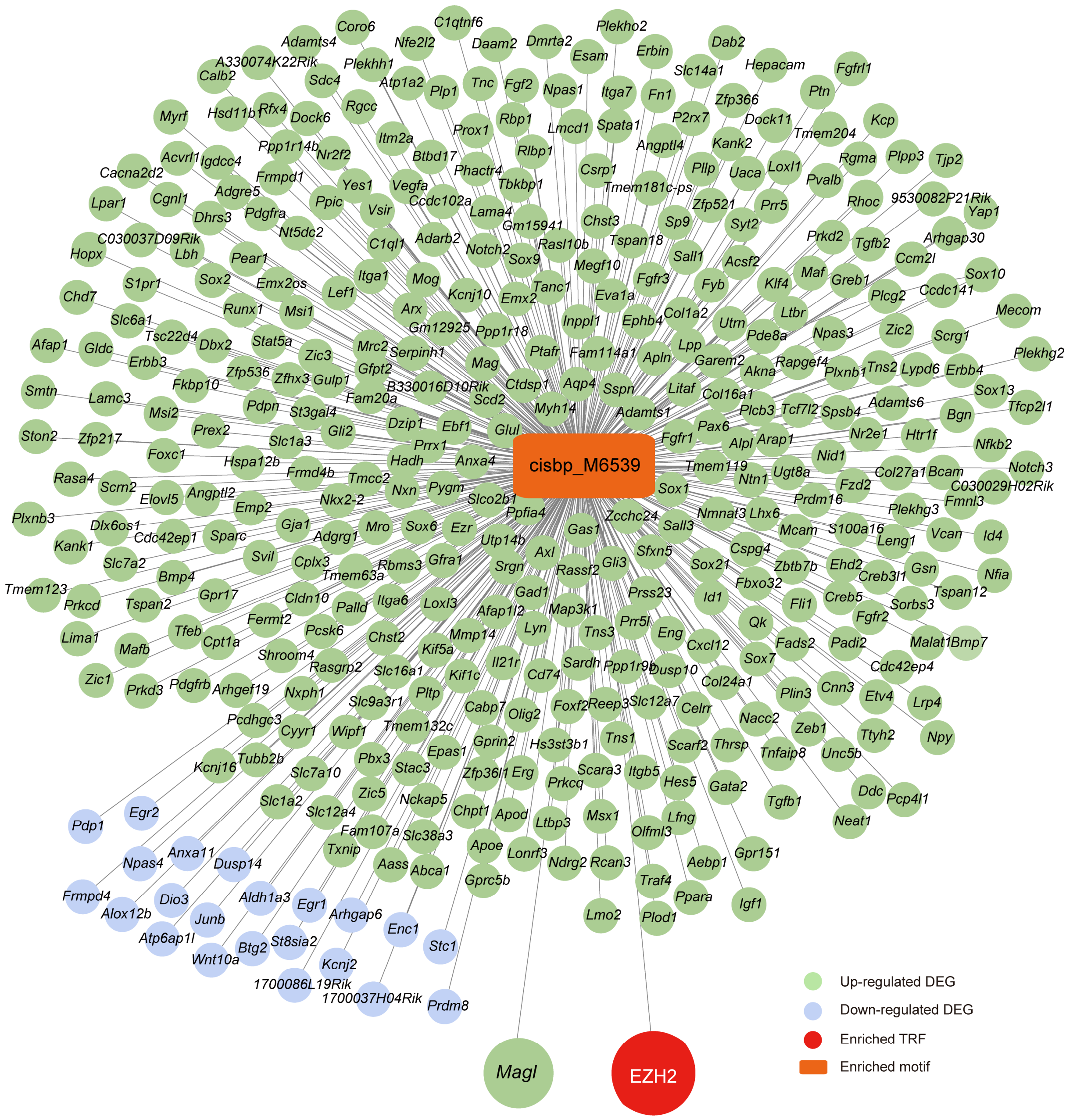

Supplemental Figure 11. EZH2 regulates the transcription of DEGs via the cisbp_M6539 motif. The regulatory network involving EZH2, the cisbp_M6539 motif and DEGs was analyzed using the RcisTarget algorithm. EZH2 may regulate the transcription of 395 up-regulated (green) and 22 down-regulated (purple) DEGs via cisbp_M6539 motif.

Supplemental Figure 12

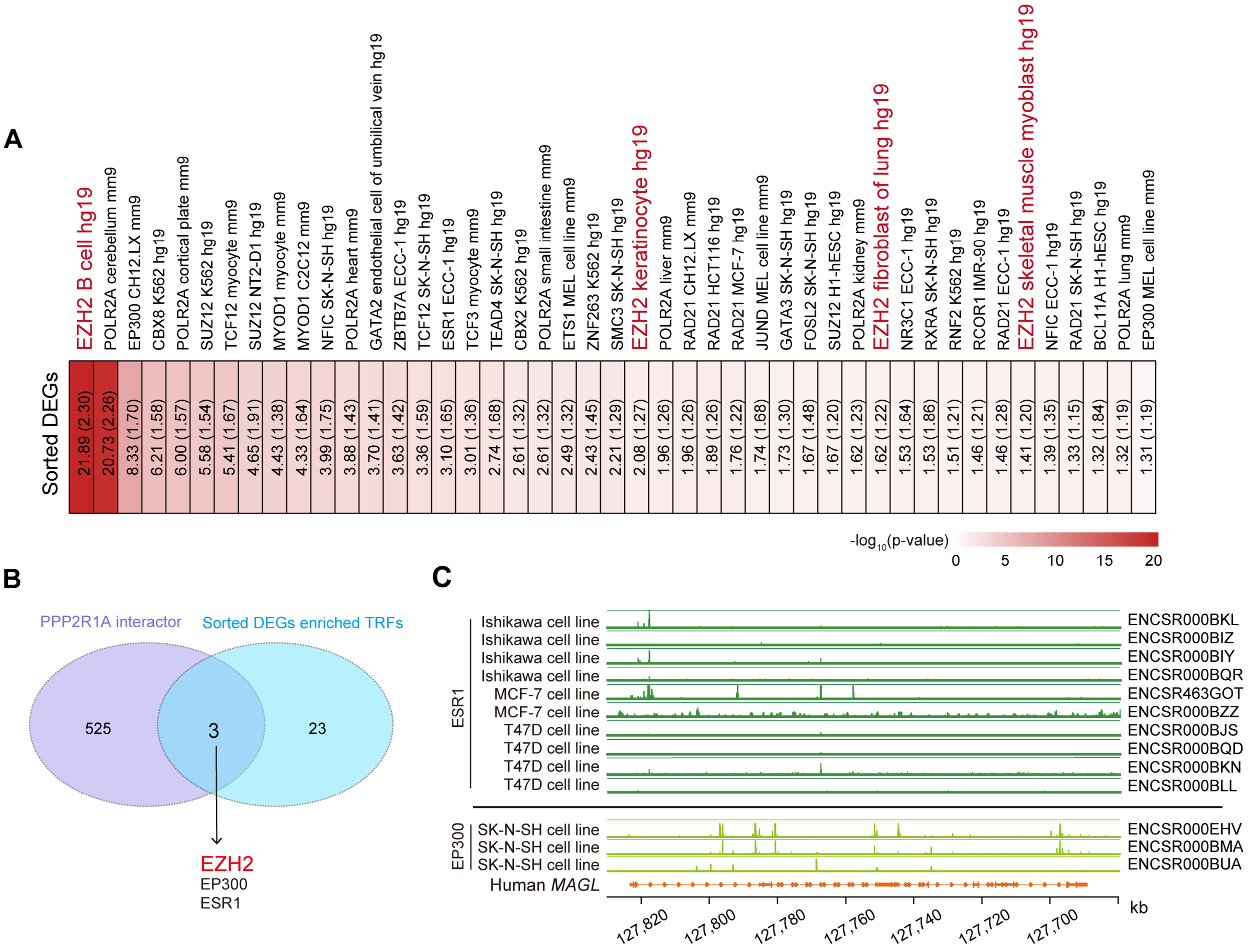

Supplemental Figure 12. Transcription regulatory factor (TRF) overrepresentation analyses identify EZH2 as a key candidate for regulating *Magl* transcription.

(**A**) TRF overrepresentation analyses based on ENCODE TRFs Chip-seq gene set library**.** DEGs identified from sorted RNA-Seq exhibited partially significant enrichment profiles for upstream regulators. The numbers outside the parentheses indicate the -$\log_{10}$ (p value), and the values in parentheses represent the odds ratio (OR).

(**B**) Intersectional analysis suggested that EZH2, EP300, and ESR1 might interact with PPP2R1A and regulate the transcription of DEGs. The purple oval represents proteins that interact with PPP2R1A, while the blue oval represents the enriched upstream TRFs according to the DEGs identified in sorted RNA-seq.

(**C**) The promoter region of *Magl* exhibited negligible or minimal binding signals for ESR1 and EP300.

Statistical comparisons were performed using one-tailed Fisher's exact test (**A**) (Supplemental Table 3).

Supplemental Figure 13

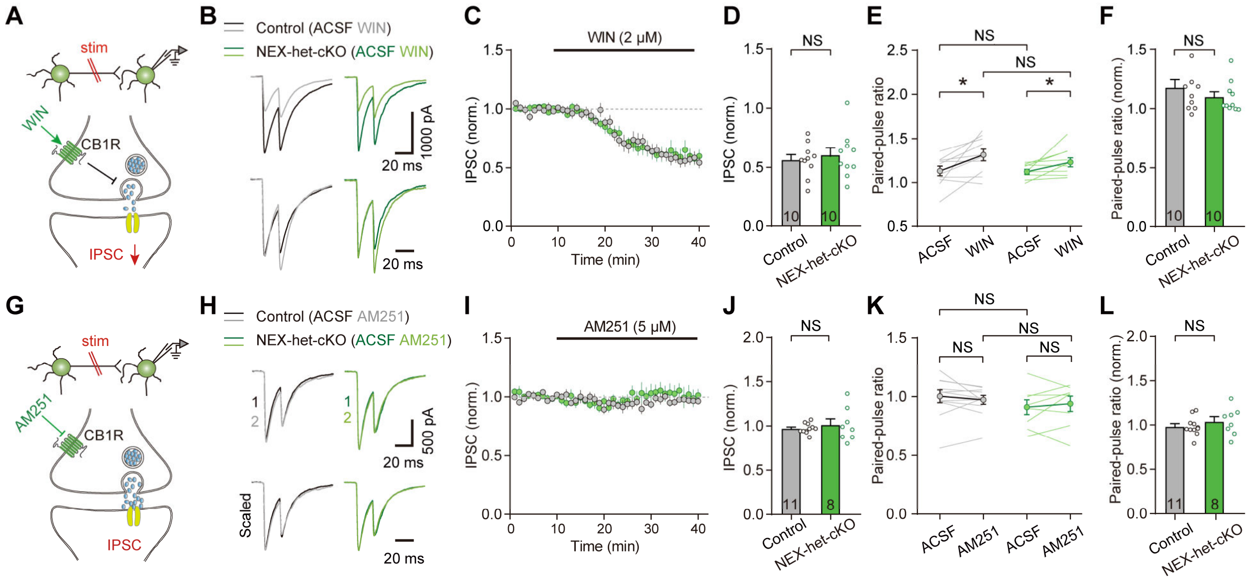

Supplemental Figure 13. ***Ppp2r1a* haploinsufficiency does not affect endocannabinoid signal at inhibitory synapses.**

(**A-F**) The inhibitory effect of CB1 receptor activation at inhibitory synapses remained unchanged in NEX-het-cKO mice (Control, n = 10 neurons; NEX-het-cKO, n = 10 neurons). (**A**) Schematic of WIN experiment. (**B**) Representative IPSC traces before and after WIN (2 µM) application. (**C**) Normalized time course summary of average IPSC amplitude during WIN application. (**D**) NEX-het-cKO mice showed unchanged WIN-mediated inhibition of IPSC amplitude. (**E**) The increase in IPSC PPRs induced by WIN was similar between control and NEX-het-cKO mice. (**F**) NEX-het-cKO mice showed a normal increase in PPR of IPSCs induced by WIN.

(**G-L**) There was no tonic eCB release at the inhibitory synapses in the mPFC (Control, n = 11 neurons; NEX-het-cKO, n = 8 neurons). (**G**) Schematic of AM251 experiment. (**H**) Representative IPSC traces before and after AM251 (5 µM) application. (**I**) Same as panel (**C**) but for AM251. (**J**) AM251 did not significantly alter IPSC amplitude in the mPFC in either genotype. (**K**) AM251 application did not alter IPSC PPRs. (**L**) Similar as panel (**F**) but for AM251.

Statistical comparisons were performed using two-tailed unpaired Student's *t*-test (**D-E**, **K-L**), two-tailed Mann-Whitney test (**F**, **K**), two-tailed unpaired Student's t-test with Welch's correction (**J**), two-tailed Wilcoxon test (**K**) and two-tailed paired Student's *t*-test (**E**, **K**). All data are presented as mean ± SEM. NS, non-significant, $*P < 0.05$.

Supplemental Figure 14

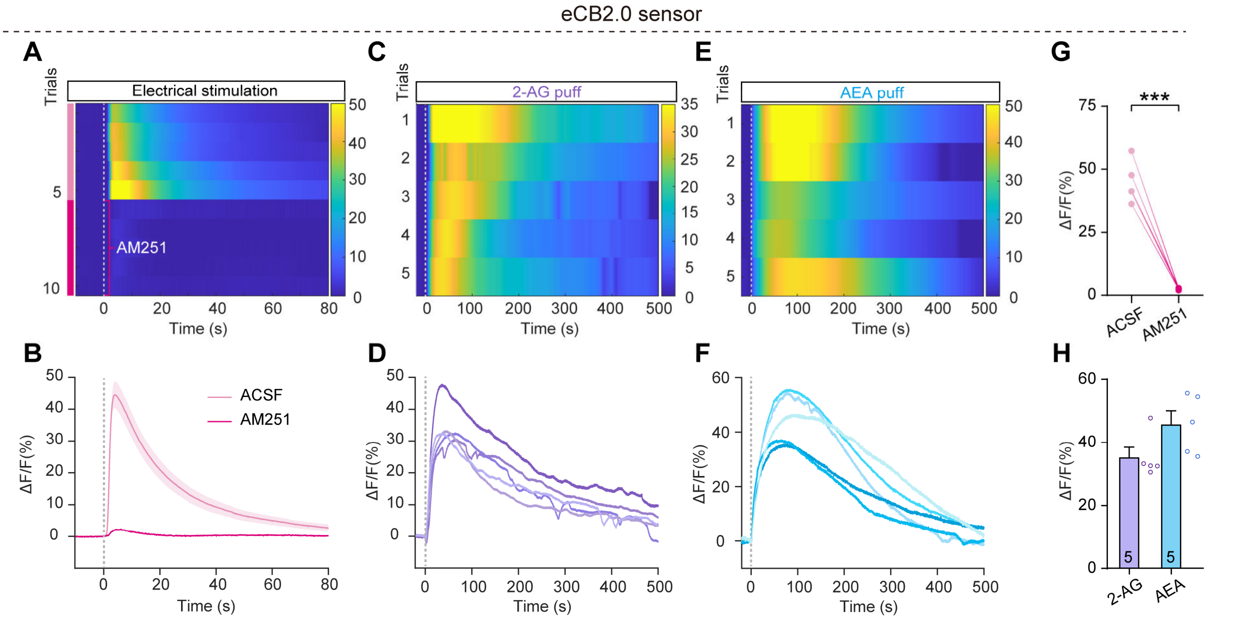

Supplemental Figure 14. Fluorescent $GRAB_{eCB2.0}$ signals in the mPFC increase specifically in response to electrical stimulation or puff application of 2-AG and AEA.

(**A-B**) Heatmap (**A**) and time course summary (**B**) illustrate that electrical stimulation-evoked $GRAB_{eCB2.0}$ signals were completely blocked by AM251 (5 μM) treatment. Pre-treatment trials (#1-5) are shown in light red, and post-AM251 trials (#6-10) are shown in dark red.

(**C-D**) Heatmap (**C**) and time course summary (**D**) demonstrate that $GRAB_{eCB2.0}$ sensor was highly responsive to puff application of 2-AG (200 μM).

(**E-F**) Same as panel (**C-D**) but for AEA (100 μM) application.

(**G**) Line chart illustrating the selective inhibition of fluorescent $GRAB_{eCB2.0}$ signals by AM251 (n = 5 slices from 1 mouse).

(**H**) Summary graph showing amplitudes of $GRAB_{eCB2.0}$ signals in response to puff application of 2-AG or AEA (2-AG, n = 5 slices from 1 mouse; AEA, n = 5 slices from 1 mouse).

Statistical comparisons were performed using two-tailed paired Student's *t*-test (**G**). All data are presented as mean ± SEM. $***P < 0.001$.

Supplemental Figure 15

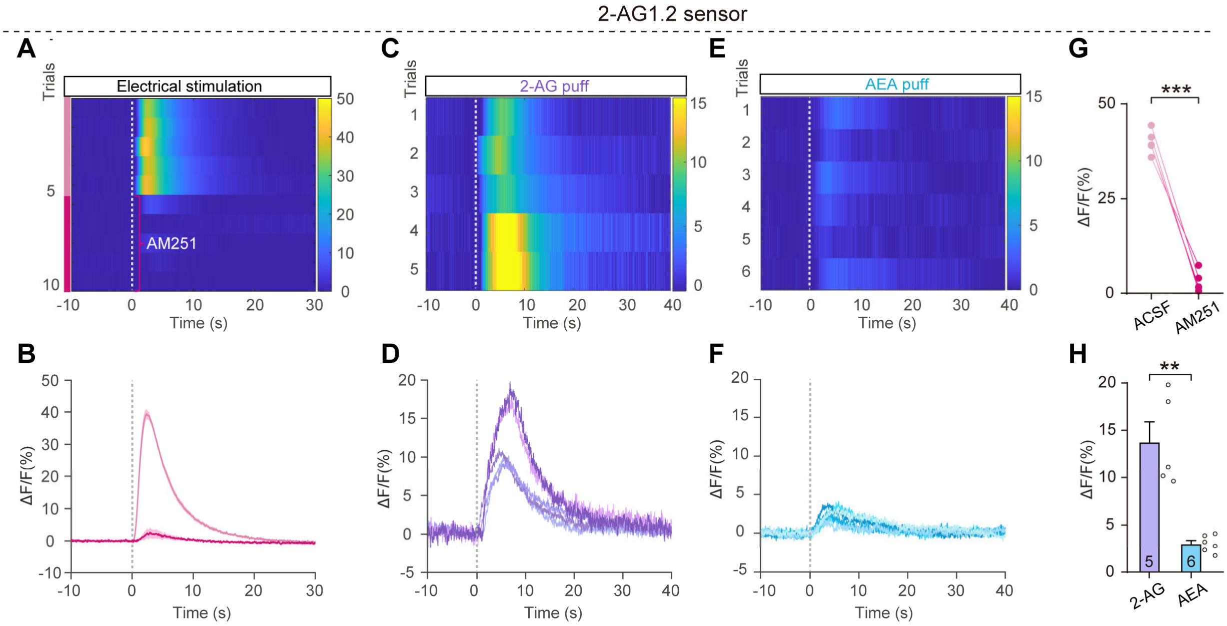

Supplemental Figure 15. High specificity of 2AG sensor GRAB$_{2\text{-AG1.2}}$.

(**A-B**) Heatmap (**A**) and time course summary (**B**) illustrate that electrical stimulation-evoked fluorescent GRAB$_{2\text{-AG1.2}}$ (also referred to as 2-AG1.2) signals were blocked by AM251 (5 µM). Pre-treatment trials (#1-5) are shown in light red, and post-AM251 trials (#6-10) are shown in dark red.

(**C-D**) Heatmap (**C**) and time course summary (**D**) show that GRAB$_{2\text{-AG1.2}}$ sensor was highly responsive to puff application of 2-AG (400 µM).

(**E-F**) Heatmap (**E**) and time course summary (**F**) indicate that GRAB$_{2\text{-AG1.2}}$ sensor was poorly responsive to the application of AEA (100 µM).

(**G**) Line chart demonstrates the selective inhibition of GRAB$_{2\text{-AG1.2}}$ signals by AM251 treatment (n = 5 slices from 1 mouse).

(**H**) Summary graph shows that the GRAB$_{2\text{-AG1.2}}$ sensor has higher sensitivity to 2-AG treatment compared to AEA treatment (2-AG, n = 5 slices from 1 mouse; AEA, n = 6 slices from 1 mouse).

Statistical comparisons were performed using two-tailed paired Student's *t*-test (**G**) and two-tailed unpaired Student's *t*-test with Welch's correction (**H**). All data are presented as mean ± SEM. $^{**}P < 0.01$, $^{***}P < 0.001$.

Supplemental Figure 16

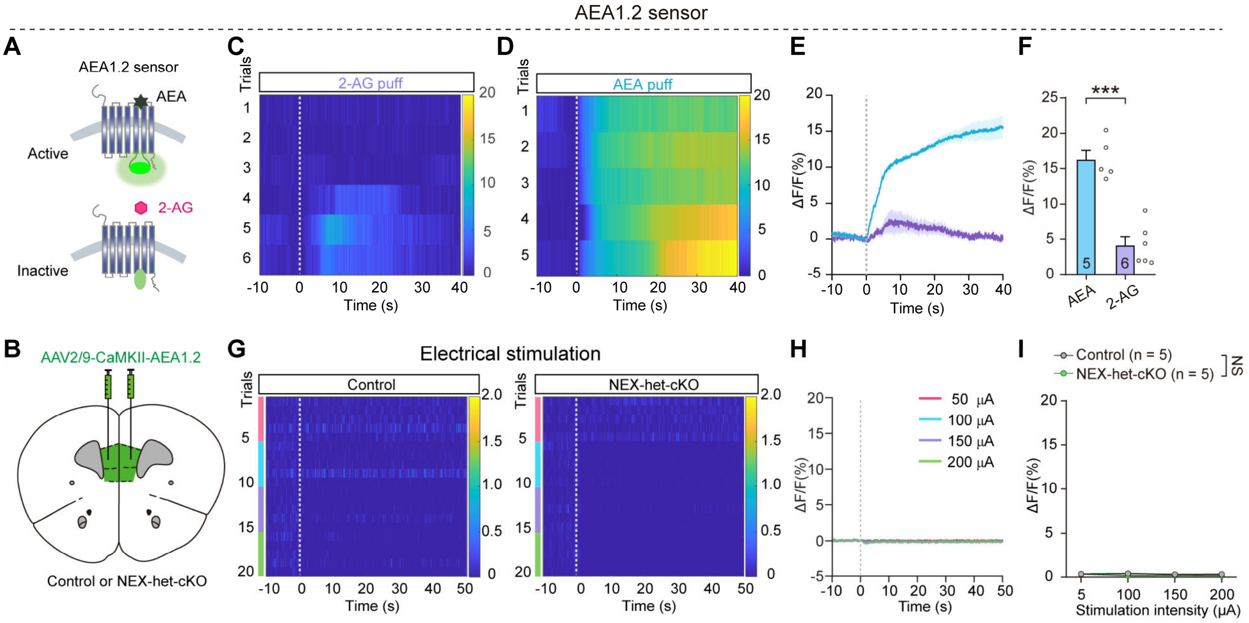

Supplemental Figure 16. GRAB$_{AEA1.2}$ reveals lack of AEA release in the mPFC upon electrical stimulation.

(**A-F**) GRAB$_{AEA1.2}$ specifically responded to AEA instead of 2-AG (AEA, n = 5 slices from 1 mouse; 2-AG, n = 6 slices from 1 mouse). (**A**) Schematic of virus injection. (**B**) Schematic of GRAB$_{AEA1.2}$ (also referred to as AEA1.2) activation. (**C**) Heatmap of GRAB$_{AEA1.2}$ signals in response to 2-AG (400 μM) puff application. (**D**) Similar to panel (**C**), but for AEA (100 μM) application. (**E**) Time course summary showing GRAB$_{AEA1.2}$ signals in response to AEA or 2-AG puff application. (**F**) Summary graph showing that GRAB$_{AEA1.2}$ has higher sensitivity to AEA treatment compared to 2-AG treatment.

(**G-I**) Electrical stimulation in brain slices did not elicit detectable responses of GRAB$_{AEA1.2}$ signals (Control, n = 5 mice; NEX-het-cKO, n = 5 mice). (**G**) Heatmap showing no changes in GRAB$_{AEA1.2}$ signals in response to electrical stimulation in both control (left) and NEX-het-cKO (right) mice. (**H**) Time course summary indicating no AEA release in response to electrical stimulation in mPFC slices of control mice. (**I**) Summary graph showing no detectable GRAB$_{AEA1.2}$ signals.

Statistical comparisons were performed using two-tailed unpaired Student's *t*-test (**F**) and two-way ANOVA followed by Bonferroni *post-hoc* test (**I**). All data are presented as mean ± SEM. NS, non-significant, $***P < 0.001$.

Supplemental Figure 17

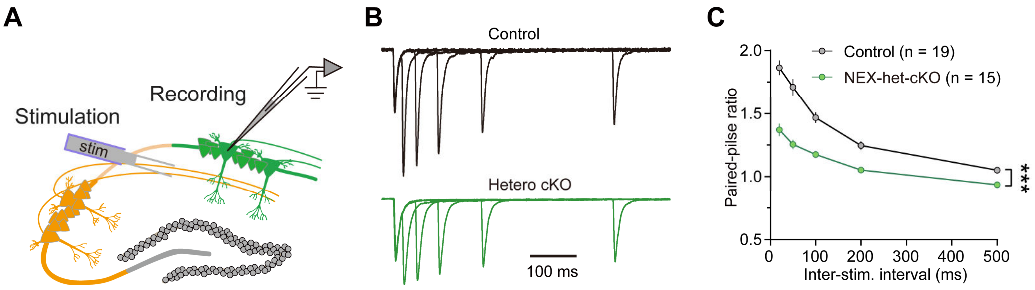

Supplemental Figure 17. *Ppp2r1a* haploinsufficiency increases presynaptic release probability of hippocampal CA3-CA1 synapses in NEX-het-cKO mice.

(**A**) Schematic of electrophysiological recording in the dCA1.

(**B**) Representative traces of paired-pulse ratio (PPR) of EPSCs in response to stimulation of the CA3-CA1 pathway.

(**C**) Summary graph showed decreased PPR in the dCA1 of NEX-het-cKO mice (Control, n = 19 neurons; NEX-het-cKO, n = 15 neurons).

Statistical comparisons were performed using two-way ANOVA followed by Bonferroni *post-hoc* test (**C**). All data are presented as mean ± SEM. ***$P < 0.001$.

Supplemental Figure 18

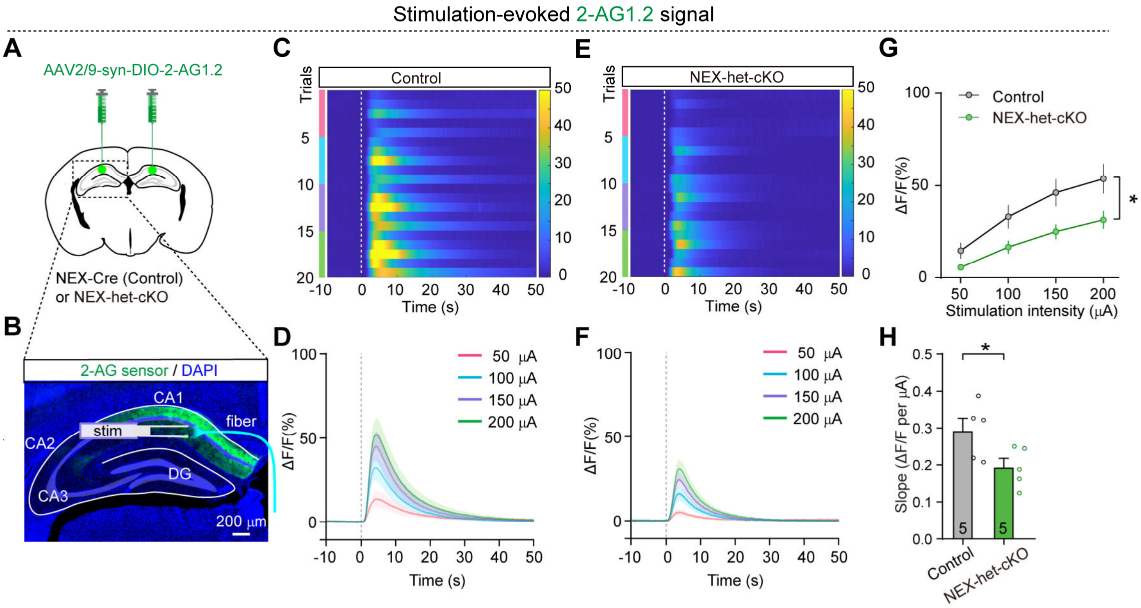

Supplemental Figure 18. 2-AG release is impaired in the hippocampal CA1 region of NEX-het-cKO mice.

(**A**) Schematic of AAV-Syn-DIO-2-AG1.2 injection in the dCA1 of NEX-Cre (Control) or NEX-het-cKO mice.

(**B**) Representative fiber photometry recordings of $GRAB_{2\text{-}AG1.2}$ signals from the dCA1 region.

(**C**) Heatmap of $GRAB_{2\text{-}AG1.2}$ signals from individual mice aligned to stimulation onset, with stimulation intensities indicated by different colors.

(**D**) Average traces of $GRAB_{2\text{-}AG1.2}$ signals across all animals from control group.

(**E-F**) Same as panel (**C-D**) but for NEX-het-cKO mice.

(**G**) Input-output curve of $GRAB_{2\text{-}AG1.2}$ signal amplitude plotted against stimulation intensity revealed significantly reduced 2-AG release in NEX-het-cKO mice (Control, n = 5 mice; NEX-het-cKO, n = 5 mice).

(**H**) Reduced slope of input/output relationship in NEX-het-cKO mice.

Statistical comparisons were performed using two-way ANOVA followed by Bonferroni *post-hoc* test (**G**) and two-tailed unpaired Student's *t*-test (**H**). All data are presented as mean ± SEM. $^*P < 0.05$

Supplemental Figure 19

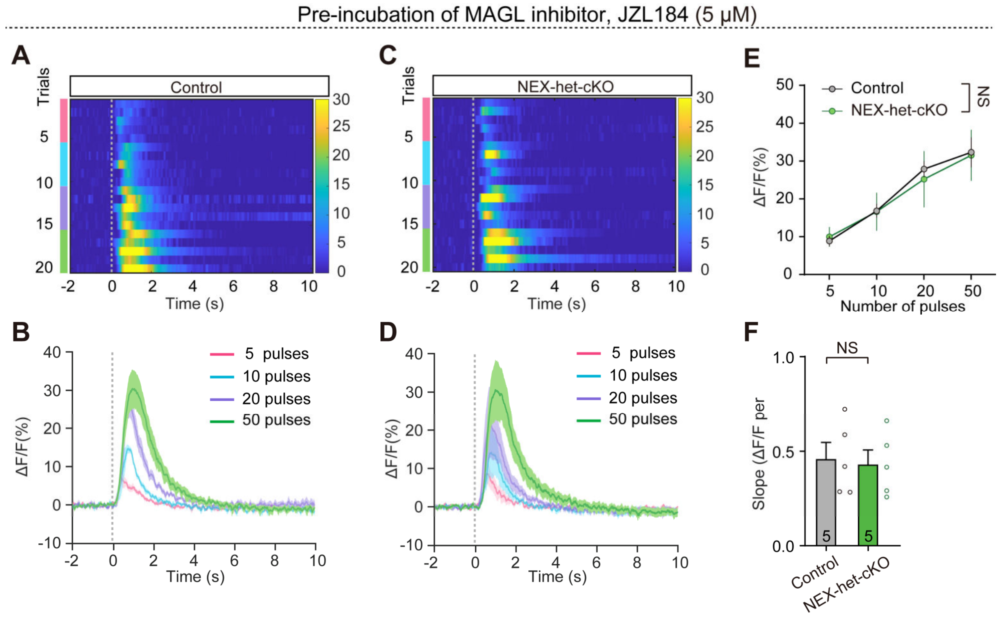

Supplemental Figure 19. JZL184 eliminates differences in presynaptic calcium signals between control and NEX-het-cKO mice.

(**A-B**) Following pre-incubation with JZL184 (5 µM), GCaMP signals in control mice were recorded during train stimulation (5, 10, 20, and 50 pulses). Fiber photometry experiments were performed under the same conditions as in **Figure 4F-4M**. (**A**) Heatmap of GCaMP signals from individual mice aligned to stimulation onset, with number of pulses indicated by different colors. (**B**) Average traces of GCaMP signals across all control mice.

(**C-D**) Similar to panel (**A-B**), but for NEX-het-cKO mice.

(**E**) Input-output plot of GCaMP signals versus number of pulses illustrating that JZL184 abolished the previously observed enhancement of axonal Ca^{2+} transients in NEX-het-cKO mice (Control, n = 5 mice; NEX-het-cKO, n = 5 mice).

(**F**) Slope of input/output relationship.

Statistical comparisons were performed using two-tailed unpaired Student's *t*-test (**F**) and two-way ANOVA followed by Bonferroni *post-hoc* test (**E**). All data are presented as mean ± SEM. NS, non-significant.

Supplemental Table 4 | Information of mouse lines and reagents

REAGENT or RESOURCE	SOURCE	IDENTIFIER
**Experimental models: Strains / Cell lines**		
Mouse: *Ppp2r1a* cKO	Jackson Laboratory	JAX#: 017441<br>RRID: IMSR_JAX:017441
Mouse: NEX-Cre	Mouse Genome Informatics	MGI#:2668659<br>RRID: MGI: 2668659
Mouse: CMV-Cre	Jackson Laboratory	JAX#: 006054<br>RRID: IMSR_JAX:006054
Mouse: Rosa26-LSL-H2B-GFP (LSL-H2B-GFP)	Jackson Laboratory	JAX#: 036761<br>RRID: IMSR_JAX:036761
Cell: N2a (Neuro-2a)	Procell	CL-0168
**Antibodies**		
GluA1 Monoclonal antibody	Proteintech	Cat #67642-1-Ig
GluA2 Polyclonal antibody	Proteintech	Cat #11994-1-AP
GluN1 Polyclonal antibody	Proteintech	Cat #27676-1-AP
GluN2A Polyclonal antibody	Proteintech	Cat #19953-1-AP
GluN2B Polyclonal antibody	Proteintech	Cat #21920-1-AP
PSD-95 Monoclonal Antibody	Thermo Fisher Scientific	Cat #MA1-045
PLCβ3 Monoclonal antibody	Proteintech	Cat #66668-1-Ig
MAGL Polyclonal antibody	Proteintech	Cat #14986-1-AP
FAAH Polyclonal antibody	Proteintech	Cat #17909-1-AP
COX2 Polyclonal antibody	Thermo Fisher Scientific	Cat #12375-1-AP
PPP2R1A Polyclonal antibody	Proteintech	Cat #15882-1-AP
CB1R Polyclonal antibody	Proteintech	Cat #17978-1-AP
NAPE-PLD Polyclonal antibody	Abcam	Cat #ab95397
β-actin Monoclonal antibody	Proteintech	Cat #66009-1-Ig
β-tubulin Monoclonal antibody	Thermo Fisher Scientific	Cat #66240-1-Ig
GAPDH Monoclonal antibody	Proteintech	Cat #60004-1-Ig
NAAA Monoclonal Antibody	Santa Cruz	Cat #sc-100470
EZH2 Monoclonal antibody	Abcam	Cat No. ab307646
EZH2 Monoclonal antibody	Proteintech	Cat No. 66476-1-Ig
PP2A-Cα Polyclonal Antibody	Immunoway	Cat #YT3830
vGAT Polyclonal Antibody	Synaptic Systems	Cat #131004
NeuN Monoclonal antibody	Abcam	Cat #ab177487
Goat anti-guinea Pig Alexa Fluor 488	Invitrogen	Cat #A-11073

Goat anti-rabbit Alexa Fluo 546	Invitrogen	Cat #A-11035
Goat anti-mouse Alexa Fluor Plus 800	Thermo Fisher Scientific	Cat #A32730
Goat anti-rabbit Alexa Fluor Plus 800	ThermoFisher Scientific	Cat #A32735
Goat anti-guinea Pig DyLight 800	ThermoFisher Scientific	Cat #SA5-10100
**AAV vectors**		
AAV2/9-CAG-DIO-GCamp6m	Taitool	NA
AAV2/9-EF1α-DIO-eCB2.0	Brain Case	NA
AAV2/9-Syn-DIO-2AG1.2	Brain Case	NA
AAV2/9-CaMKII-AEA1.2	Brain Case	NA
AAV2/9-Ef1α-DIO-EYFP	Brain Case	NA
**Chemicals**		
Picrotoxin	Tocris	Cat #1128
Tetrodotoxin citrate	Tocris	Cat #1069
CNQX disodium salt	Tocris	Cat # 1045
D-AP5	Tocris	Cat # 0106
(+)-MK801 maleate (MK801)	Tocris	Cat # 0924
QX 314 bromide	Tocris	Cat # 1014
WIN 55,212-2 mesylate (WIN)	Tocris	Cat # 1038
AM251	Tocris	Cat # 1117
(S)-3,5-DHPG	MCE	Cat # HY-12598
DO34	MCE	Cat # HY-117771
2-Arachidonoyl Glycerol (2-AG)	GlpBio	Cat # GC16403
Anandamide (AEA)	GlpBio	Cat # GC35339
JZL184	Tocris	Cat # 3836
URB597	Tocris	Cat # 4612
Okadaic acid sodium salt (OA)	Abcam	Cat # ab141831
EPZ011989	MCE	Cat. No. HY-16986
Cycloheximide (CHX)	MCE	Cat. No. HY-12320
**Commercial kits**		
Arcturus PicoPure RNA Isolation Kit	Thermo Fisher Scientific	Cat # KIT0204
Agilent RNA 6000 Pico Kit	Agilent	Cat # 5067-1513
Agilent High Sensitivity DNA Kit	Agilent	Cat # 5067- 4626
Qubit dsDNA HS Kit	ThermoFisher Scientific	Cat. No. Q32851
Qubit RNA assay kits	ThermoFisher Scientific	Cat. No. Q32855
SMARTer Stranded Total RNA-Seq Kit v2 Pico Input Mammalian	Takara	Cat # 634412

BCA Protein Colorimetric Assay Kit	Elabscience	Cat # E-BC-K318-M
One-Step PAGE Gel Fast Preparation Kit	Vazyme	Cat # E302-E305
Syn-PER™ reagent	Thermo Fisher Scientific	Cat # 87793
HiScript III RT SuperMix	Vazyme	Cat # R323-01
ChamQ Universal SYBR qPCR Master Mix	Vazyme	Cat # Q711
TruSeq RNA Sample Preparation Kit v2	Illumina	Cat # RS-122-2001,
Experion DNA 1K Analysis Kit	BIO-RAD	Cat # 7007107
Revert 700 Total Protein Stain Kit	LI-COR	Cat # 926-11010

Oligonucleotides

**Research or Resource**	**Sequence**
q-PCR primer sequence of *β-actin* mRNA:	*β-actin* - Forward: 5′-GGCTGTATTCCCCTCCATCG-3′<br>*β-actin* - Reverse: 5′-CCAGTTGGTAACAATGCCATGT-3′
q-PCR primer sequence of *Magl* mRNA:	*Magl* - Forward: 5′- CGGACTTCCAAGTTTTTGTCAGA-3′<br>*Magl* - Reverse: 5′- GCAGCCACTAGGATGGAGATG-3′
q-PCR primer sequence of *Faah* mRNA:	*Faah* - Forward: 5′- GAGGCTCCCCTCTGGGTTTA-3′<br>*Faah* - Reverse: 5′- GCCAGGCTATCCACATCCC-3′
Genotyping primers for *Ppp2r1a* cKO mouse:	*Ppp2r1a* - Forward: 5′-AGG ACA AGT CCT GGC GTG-3′<br>*Ppp2r1a* - Reverse: 5′-GAA TTA AAC CCA GGA CCC CTG-3′
Genotyping primers for NEX-Cre mouse:	NEX-Cre -common- Forward: 5′- GAGTCCTGGAATCAGTCTTTTTC-3′<br>NEX-Cre - wildtype - Reverse: 5′-AGAATGTGGAGTAGGGTGAC-3′<br>NEX-Cre - mutant - Reverse: 5′-CCGCATAACCAGTGAAACAG-3′
Genotyping primers for CMV-Cre mouse:	CMV-Cre -Forward: 5′- CAT CCA CGC TGT TTT GAC CT-3′<br>CMV-Cre - Reverse: 5′- AGG CAA ATT TTG GTG TAC GG-3′
Genotyping primers for LSL-H2B-GFP mouse:	H2B-GFP - wild type - Forward: 5′- CTG GCT TCT GAG GAC CG-3′<br>H2B-GFP - wild type- Reverse: 5′-CAG GAC AAC GCC CAC ACA-3′<br>H2B-GFP - mutant - Forward: 5′-ACC TCC AGG GAG ATC CAG AC-3′<br>H2B-GFP - mutant - Reverse: 5′- CTG AAC TTG TGG CCG TTT AC-3′
Genotyping primers for *Ppp2r1a* KO mouse:	*Ppp2r1a* - Forward: 5′-ACAGTAGTGCGAGACAAGGC-3′<br>*Ppp2r1a* - Reverse: 5′- CGTTATACATGGTTGTGAGCAAAC-3′

Supplemental Table 5 | Summary of all experimental designs

(Related to Figures 1-10 and S1-S19)

Figures	Aims	Mouse lines / Cell lines	Virus injection	Type of data
Figure S1	To examine the spatiotemporal expression pattern of PPP2R1A	WT	N/A	Western blotting
	To investigate the mRNA expression patterns of *Ppp2r1a* in different cell types	N/A	N/A	Re-analysis of single-nucleus RNA-Seq data
Figure 1	To elaborate on the conditional knockout strategy for the *Ppp2r1a* gene	Hom-*Ppp2r1a*-cKO (Hom-cKO)<br>NEX-Cre	N/A	N/A
	To investigate the lethality of homozygous *Ppp2r1a* deletion in NEX+ neurons	Het-*Ppp2r1a*-cKO (Het-cKO)<br>Hom-*Ppp2r1a*-cKO (Hom-cKO)<br>NEX-Cre; het-*Ppp2r1a*-cKO (NEX-het-cKO)<br>NEX-Cre; hom-*Ppp2r1a*-cKO (NEX-hom-cKO)		Genotyping
	To study the effect of *Ppp2r1a* knockout in forebrain excitatory neurons on the weight of mice	Het-*Ppp2r1a*-cKO (Ctrl)<br>NEX-Cre; het-*Ppp2r1a*-cKO (NEX-het-cKO)		Weight measurements
	To compare the behavioral performance of control and NEX-het-cKO mice	Het-*Ppp2r1a*-cKO (Ctrl)<br>NEX-Cre; het-*Ppp2r1a*-cKO (NEX-het-cKO)		Behavior
Figure S2	To reveal the spatial distribution of NEX+ neurons in the brain	NEX-Cre; LSL-H2B-GFP	N/A	Morphology
	To study the effect of *Ppp2r1a* knockout in excitatory neurons on the weight and size of the brain	Het-*Ppp2r1a*-cKO (Ctrl)<br>NEX-Cre; het-*Ppp2r1a*-cKO (NEX-het-cKO)		Weight and size measurements
Figure S3<br>Figure S4	To compare the behavioral performance of control and NEX-het-cKO mice	Het-*Ppp2r1a*-cKO (Ctrl)<br>NEX-Cre; het-*Ppp2r1a*-cKO (NEX-het-cKO)	N/A	Behavior
Figure 2	To record the excitatory synaptic inputs to layer 5 pyramidal neurons in control and NEX-het-cKO mice	Het-*Ppp2r1a*-cKO (Ctrl)<br>NEX-Cre; het-*Ppp2r1a*-cKO (NEX-het-cKO)	N/A	Slice physiology
	To record the inhibitory synaptic inputs to layer 5 pyramidal neurons in control and NEX-het-cKO mice			
	To compare the E/I balance in layer 5 pyramidal neurons of control and NEX-het-cKO mice			
Figure S5	To quantify the number of inhibitory synapses in the mPFC of control and NEX-het-cKO mice	Het-*Ppp2r1a*-cKO (Ctrl)<br>NEX-Cre; het-*Ppp2r1a*-cKO (NEX-het-cKO)	N/A	Histology
Figure S6	To measure the reversal potentials of EPSCs and IPSCs	Het-*Ppp2r1a*-cKO (Ctrl)	N/A	Slice physiology

Figure 3	To compare the dendritic complexity and synaptic density of layer 5 pyramidal neurons in control and NEX-het-cKO mice	Het-*Ppp2r1a*-cKO (Ctrl)<br>NEX-Cre; het-*Ppp2r1a*-cKO (NEX-het-cKO)	N/A	Slice physiology<br>Morphology
Figure S7	To reconstruct the morphology of layer 5 pyramidal neurons in control and NEX-het-cKO mice	Het-*Ppp2r1a*-cKO (Ctrl)<br>NEX-Cre; het-*Ppp2r1a*-cKO (NEX-het-cKO)	N/A	Slice physiology<br>Morphology
Figure 4	To record the presynaptic release probability in layer 5 pyramidal neurons of control and NEX-het-cKO mice	Het-*Ppp2r1a*-cKO (Ctrl)<br>NEX-Cre; het-*Ppp2r1a*-cKO (NEX-het-cKO)	N/A	Slice physiology
	To record the Ca^{2+} transients of the vCA1 → mPFC projections in control and NEX-het-cKO mice		Bilateral injection of AAV-CAG-DIO - GCaMP6m into the vCA1	Fiber photometry recording
Figure S8	To examine the protein expression levels of postsynaptic glutamate receptors in control and NEX-het-cKO mice	Het-*Ppp2r1a*-cKO (Ctrl)<br>NEX-Cre; het-*Ppp2r1a*-cKO (NEX-het-cKO)	N/A	Western blotting
Figure S9	To quantify the proportion of neurons expressing GCaMP6m in the vHIP of control and NEX-het-cKO mice	NEX-Cre (Ctrl)<br>NEX-Cre; het-*Ppp2r1a*-cKO (NEX-het-cKO)	N/A	Histology
Figure 5<br>Figure S10	To analyze the transcriptomic changes in NEX^{+} neurons of control and NEX-het-cKO mice	NEX-Cre (Ctrl)<br>NEX-Cre; het-*Ppp2r1a*-cKO (NEX-het-cKO)	Bilateral injection of AAV-Ef1α-DIO-EYFP into the mPFC	FACS<br>Sorted RNA sequencing<br>GO and KEGG enrichment analyses
Figure 6	To examine the protein/mRNA expression levels of eCB-related enzymes in control and NEX-het-cKO mice	Wildtype (Ctrl)<br>Het-*Ppp2r1a*-KO<br>N2A cell line	N/A	Western blot<br>RT-qPCR
Figure 7<br>Figure S11<br>Figure S12	To determine the transcription regulatory factor (TRF) through which PPP2R1A regulates *Magl* transcription.	NEX-Cre (Ctrl)<br>NEX-Cre; het-*Ppp2r1a*-cKO	Bilateral injection of AAV-Ef1α-DIO-EYFP into the mPFC	TFs motif enrichment analyses<br>Chip-Seq analyses<br>TFs Overrepresentation analyses
		WT<br>Het-*Ppp2r1a*-KO<br>N2A cell line	N/A	Western blot<br>RT-qPCR
Figure 8	To compare the strength of eCB signaling in excitatory synapses from control and NEX-het-cKO mice	Het-*Ppp2r1a*-cKO (Ctrl)<br>NEX-Cre; het-*Ppp2r1a*-cKO (NEX-het-cKO)	N/A	Slice physiology
Figure S13	To compare the strength of eCB signaling in inhibitory synapses from control and NEX-het-cKO mice	Het-*Ppp2r1a*-cKO (Ctrl)<br>NEX-Cre; het-*Ppp2r1a*-cKO (NEX-het-cKO)	N/A	Slice physiology
Figure 9	To record the $GRAB_{eCB2.0}$ signal in the mPFC of control and NEX-het-cKO mice	Het-*Ppp2r1a*-cKO (Ctrl)<br>NEX-Cre; het-*Ppp2r1a*-cKO (NEX-het-cKO)	N/A	Slice physiology
		NEX-Cre (Ctrl)<br>NEX-Cre; het-*Ppp2r1a*-cKO (NEX-het-cKO)	Bilateral injection of AAV-EF1α-DIO-eCB2.0 into the mPFC	Fiber photometry recording

Figure S14	To examine the responses of $GRAB_{eCB2.0}$ sensor to AM251, 2-AG and AEA in the mPFC of NEX-Cre mice	NEX-Cre	Bilateral injection of AAV-EF1α-DIO-eCB2.0 into the mPFC	Fiber photometry recording
Figure 10	To assess the contribution of 2-AG to the $GRAB_{eCB2.0}$ signal in the mPFC of control and NEX-het-cKO mice	NEX-Cre (Ctrl)	Bilateral injection of AAV-EF1α-DIO-eCB2.0 into the mPFC	Fiber photometry recording
	To record the $GRAB_{2\text{-}AG1.2}$ signal in the mPFC of control and NEX-het-cKO mice	Het-*Ppp2r1a*-cKO (Ctrl) NEX-Cre; het-*Ppp2r1a*-cKO (NEX-het-cKO)	Bilateral injection of AAV-Syn-DIO-2AG1.2 into the mPFC	
	To test the effect of JZL184 and URB597 on EPSC PPRs in the mPFC of control and NEX-het-cKO mice	Het-*Ppp2r1a*-cKO (Ctrl) NEX-Cre; het-*Ppp2r1a*-cKO (NEX-het-cKO)	N/A	Slice physiology
	To test the effect of JZL184 on the behavioral performance of control and NEX-het-cKO mice	Het-*Ppp2r1a*-cKO (Ctrl) NEX-Cre; het-*Ppp2r1a*-cKO (NEX-het-cKO)	N/A	Behavior
Figure S15	To examine the specificity of $GRAB_{2\text{-}AG1.2}$ sensor in the mPFC	NEX-Cre (Ctrl)	Bilateral injection of AAV-Syn-DIO-2AG1.2 into the mPFC	Fiber photometry recording
Figure S16	To examine the responses of $GRAB_{AEA1.2}$ sensor to electrical stimulation, AEA and 2-AG.	Het-*Ppp2r1a*-cKO (Ctrl) NEX-Cre; het-*Ppp2r1a*-cKO (NEX-het-cKO)	Bilateral injection of AAV-CaMKII-AEA1.2 into the mPFC	Fiber photometry recording
Figure S17	To record EPSC PPRs in dCA1 pyramidal neurons of control and NEX-het-cKO mice	Het-*Ppp2r1a*-cKO (Ctrl) NEX-Cre; het-*Ppp2r1a*-cKO (NEX-het-cKO)	N/A	Slice physiology
Figure S18	To record the $GRAB_{2\text{-}AG1.2}$ signal in the dCA1 of control and NEX-het-cKO mice	NEX-Cre (Ctrl)	Bilateral injection of AAV-Syn-DIO-2AG1.2 into the dCA1	Fiber photometry recording
Figure S19	To study the effect of JZL184 on the presynaptic Ca^{2+} transients of of control and NEX-het-cKO mice	NEX-Cre (Ctrl) NEX-Cre; het-*Ppp2r1a*-cKO (NEX-het-cKO)	Bilateral injection of AAV-CAG-DIO - GCaMP6m into the vCA1	Fiber photometry recording

Supplementary Table 6 | Summary of statistical analyses

Figure	Sample size (n)	Statistical test	Comparison	*P* values
Figure 1B	Observed value (n = 81 mice)<br>Theoretical value (n = 108 mice)	Chi-square test	Observed value vs. Expected value	$\chi^2 = 29.16$, $P < 0.0001$ ***
Figure 1E	Control (n = 13 mice)<br>NEX-het-KO (n = 15 mice)	Two-way ANOVA	Control vs. NEX-het-KO	$F_{1,26} = 2.335$, $P = 0.1386$ NS
		Post-hoc Bonferroni test	P7: Control vs. NEX-het-KO	$t_{130} = 0.03943$, $P > 0.9999$ NS
			P14: Control vs. NEX-het-KO	$t_{130} = 1.271$, $P > 0.9999$ NS
			P21: Control vs. NEX-het-KO	$t_{130} = 2.365$, $P = 0.0975$ NS
			P28: Control vs. NEX-het-KO	$t_{130} = 2.138$, $P = 0.1721$ NS
			P56: Control vs. NEX-het-KO	$t_{130} = 0.6274$, $P > 0.9999$ NS
Figure 1G	Control (n = 13 mice)<br>NEX-het-cKO (n = 12 mice)	Two-tailed unpaired Student's t-test	Control vs. NEX-het-cKO	$t_{23} = 3.096$, $P = 0.0051$ **
Figure 1I	Control (n = 17 mice)<br>NEX-het-cKO (n = 20 mice)	Two-way ANOVA	Number of errors: Control vs. NEX-het-cKO	$F_{1,35} = 47.37$, $P < 0.0001$ ***
		Post-hoc Bonferroni test	day 1: Control vs. NEX-het-cKO	$t_{280} = 1.423$, $P > 0.9999$ NS
			day 2: Control vs. NEX-het-cKO	$t_{280} = 2.640$, $P = 0.0700$ NS
			day 3: Control vs. NEX-het-cKO	$t_{280} = 2.539$, $P = 0.0932$ NS
			day 4: Control vs. NEX-het-cKO	$t_{280} = 4.378$, $P = 0.0001$ ***
			day 5: Control vs. NEX-het-cKO	$t_{280} = 1.646$, $P = 0.8071$ NS
			day 6: Control vs. NEX-het-cKO	$t_{280} = 1.964$, $P = 0.4040$ NS
			day 7: Control vs. NEX-het-cKO	$t_{280} = 2.270$, $P = 0.1917$ NS
			day 8: Control vs. NEX-het-cKO	$t_{280} = 1.854$, $P = 0.5183$ NS
Figure 1J		Two-way ANOVA	Time: Control vs. NEX-het-cKO	$F_{1,35} = 10.45$, $P = 0.0027$ **
		Post-hoc Bonferroni test	day 1: Control vs. NEX-het-cKO	$t_{280} = 3.940$, $P = 0.0008$ ***
			day 2: Control vs. NEX-het-cKO	$t_{280} = 2.525$, $P = 0.0971$ NS
			day 3: Control vs. NEX-het-cKO	$t_{280} = 0.7150$, $P > 0.9999$ NS
			day 4: Control vs. NEX-het-cKO	$t_{280} = 2.048$, $P = 0.3317$ NS
			day 5: Control vs. NEX-het-cKO	$t_{280} = 0.8198$, $P > 0.9999$ NS
			day 6: Control vs. NEX-het-cKO	$t_{280} = 1.966$, $P = 0.4020$ NS
			day 7: Control vs. NEX-het-cKO	$t_{280} = 1.219$, $P > 0.9999$ NS
			day 8: Control vs. NEX-het-cKO	$t_{280} = 1.173$, $P > 0.9999$ NS
Figure 1L	Control (n = 14 mice)<br>NEX-het-cKO (n = 16 mice)	Two-way ANOVA	**Number of primary errors** (Acquisition): Control vs. NEX-het-cKO	$F_{1,28} = 7.806$, $P = 0.0093$ **
			**Number of primary errors** (Reversal): Control vs. NEX-het-cKO	$F_{1,28} = 16.99$, $P = 0.0003$ ***
		Post-hoc Bonferroni test	day 1: Control vs. NEX-het-cKO<br>day 2: Control vs. NEX-het-cKO<br>day 3: Control vs. NEX-het-cKO	$t_{84} = 1.219$, $P = 0.6789$ NS<br>$t_{84} = 2.039$, $P = 0.1337$NS<br>$t_{84} = 2.021$, $P = 0.1394$ NS
			day 4: Control vs. NEX-het-cKO<br>day 5: Control vs. NEX-het-cKO<br>day 6: Control vs. NEX-het-cKO	$t_{84} = 1.662$, P = 0.3008 NS<br>$t_{84} = 3.331$, P = 0.0039 **<br>$t_{84} = 2.994$, P = 0.0108 *
Figure 1M		Two-way ANOVA	**Primary latency** (Acquisition): Control vs. NEX-het-cKO	$F_{1,28} = 1.112$, $P = 0.3007$ NS
			**Primary latency** (Reversal): Control vs. NEX-het-cKO	$F_{1,28} = 1.467$, $P = 0.2359$ NS

		Post-hoc Bonferroni test	day 1: Control vs. NEX-het-cKO<br>day 2: Control vs. NEX-het-cKO<br>day 3: Control vs. NEX-het-cKO	$t_{84} = 0.2191$, $P > 0.9999$ NS<br>$t_{84} = 0.9064$, $P > 0.9999$ NS<br>$t_{84} = 0.8609$, $P > 0.9999$ NS
			day 4: Control vs. NEX-het-cKO<br>day 5: Control vs. NEX-het-cKO<br>day 6: Control vs. NEX-het-cKO	$t_{84} = 0.1411$, $P > 0.9999$ NS<br>$t_{84} = 1.568$, $P = 0.3619$ NS<br>$t_{84} = 0.8323$, $P > 0.9999$ NS
Figure 1N		Two-way ANOVA	**Primary path** (Acquisition):<br>Control vs. NEX-het-cKO	$F_{1, 28} = 2.917$, $P = 0.0987$ NS
			**Primary path** (Reversal):<br>Control vs. NEX-het-cKO	$F_{1, 28} = 6.615$, $P = 0.0157$ *
		Post-hoc Bonferroni test	day 1: Control vs. NEX-het-cKO<br>day 2: Control vs. NEX-het-cKO<br>day 3: Control vs. NEX-het-cKO	$t_{84} = 0.4436$, $P > 0.9999$ NS<br>$t_{84} = 1.100$, $P = 0.8236$ NS<br>$t_{84} = 1.331$, $P = 0.5602$ NS
			day 4: Control vs. NEX-het-cKO<br>day 5: Control vs. NEX-het-cKO<br>day 6: Control vs. NEX-het-cKO	$t_{84} = 0.07215$, $P > 0.9999$ NS<br>$t_{84} = 2.574$, $P = 0.0355$ *<br>$t_{84} = 1.824$, $P = 0.2151$ NS
Figure 1O		Two-tailed unpaired Student's t-test	Direct: Control vs. NEX-het-cKO	$t_{28} = 5.667$, $P < 0.0001$ ***
			Serial: Control vs. NEX-het-cKO	$t_{28} = 3.288$, $P = 0.0027$ **
			Mixed: Control vs. NEX-het-cKO	$t_{28} = 1.325$, $P = 0.1959$ NS
Figure 2B	Control (n = 25 neurons from 4 mice)<br>NEX-het-cKO (n = 20 neurons from 4 mice)	Two-tailed Mann-Whitney test	Frequency:<br>Control vs. NEX-het-cKO	$U = 106$, $P = 0.0007$ ***
		Kolmogorov-Smirnov test	Cumulative distributions:<br>Control vs. NEX-het-cKO	$D = 0.1298$, $P < 0.0001$ ***
Figure 2C		Two-tailed unpaired Student's t-test	Amplitude:<br>Control vs. NEX-het-cKO	$t_{43} = 1.723$, $P = 0.0921$ NS
		Kolmogorov-Smirnov test	Cumulative distributions:<br>Control vs. NEX-het-cKO	$D = 0.08903$, $P < 0.0001$ ***
Figure 2E	Control (n = 20 neurons from 3 mice)<br>NEX-het-cKO (n = 18 neurons from 3 mice)	Two-way ANOVA	Control vs. NEX-het-cKO	$F_{1, 36} = 9.839$, $P = 0.0034$ **
		Post-hoc Bonferroni test	50 μA: Control vs. NEX-het-cKO	$t_{180} = 0.7181$, $P = 0.9596$ NS
			75 μA: Control vs. NEX-het-cKO	$t_{180} = 1.865$, $P = 0.2807$ NS
			100 μA: Control vs. NEX-het-cKO	$t_{180} = 3.068$, $P = 0.0123$ *
			125 μA: Control vs. NEX-het-cKO	$t_{180} = 3.604$, $P = 0.002$ **
			150 μA: Control vs. NEX-het-cKO	$t_{180} = 4.415$, $P < 0.0001$ ***
Figure 2F		Two-tailed Mann-Whitney test	Control vs. NEX-het-cKO	$U = 84$, $P = 0.0043$ **
Figure 2H	Control (n = 22 neurons from 3 mice)<br>NEX-het-cKO (n = 19 neurons from 3 mice)	Two-way ANOVA	Control vs. NEX-het-cKO	$F_{1, 39} = 13.66$, $P = 0.0007$ ***
		Post-hoc Bonferroni test	50 μA: Control vs. NEX-het-cKO	$t_{195} = 1.231$, $P = 0.711$ NS
			75 μA: Control vs. NEX-het-cKO	$t_{195} = 2.74$, $P = 0.0331$ *
			100 μA: Control vs. NEX-het-cKO	$t_{195} = 3.833$, $P = 0.0009$ ***
			125 μA: Control vs. NEX-het-cKO	$t_{195} = 4.373$, $P < 0.0001$ ***
			150 μA: Control vs. NEX-het-cKO	$t_{195} = 4.914$, $P < 0.0001$ ***
Figure 2I		Two-tailed unpaired Student's t-test with Welch's correction	Control vs. NEX-het-cKO	$t_{27.65} = 3.632$, $P = 0.0011$ **
Figure 2K	Control (n = 19 neurons from 4 mice)<br>NEX-het-cKO (n = 15 neurons from 4 mice)	Two-tailed unpaired Student's t-test	Frequency:<br>Control vs. NEX-het-cKO	$t_{32} = 2.081$, $P = 0.0456$ *
		Kolmogorov-Smirnov test	Cumulative distributions:<br>Control vs. NEX-het-cKO	$D = 0.06775$, $P < 0.0001$ ***
Figure 2L		Two-tailed unpaired Student's t-test	Amplitude:<br>Control vs. NEX-het-cKO	$t_{32} = 2.733$, $P = 0.0101$ **
		Kolmogorov-Smirnov test	Cumulative distributions:<br>Control vs. NEX-het-cKO	$D = 0.1297$, $P < 0.0001$ ***

Figure 2O	Control (n = 15 neurons from 3 mice) NEX-het-cKO (n = 16 neurons from 3 mice)	Two-tailed unpaired Student's t-test with Welch's correction	Control vs. NEX-het-cKO	$t_{22.46} = 4.469$, $P = 0.0002$ ***
Figure 3C	Control (n = 13 neurons from 4 mice) NEX-het-cKO (n = 10 neurons from 5 mice)	Two-way ANOVA with Geisser-Greenhouse correction	Control vs. NEX-het-cKO	$F_{1,21} = 0.03868$, $P = 0.8460$ NS
Figure 3D			Control vs. NEX-het-cKO	$F_{1,21} = 0.06349$, $P = 0.8035$ NS
Figure 3F	Control (n = 9 neurons from 4 mice) NEX-het-cKO (n = 12 neurons from 5 mice)	Two-tailed unpaired Student's t-test	Control vs. NEX-het-cKO	$t_{19} = 1.198$, $P = 0.2455$ NS
Figure 3H	Control (n = 7 neurons from 4 mice) NEX-het-cKO (n = 8 neurons from 4 mice)	Two-tailed unpaired Student's t-test	Control vs. NEX-het-cKO	$t_{13} = 0.8319$, $P = 0.4205$ NS
Figure 3J	Control (n = 13 neurons from 6 mice) NEX-het-cKO (n = 10 neurons from 4 mice)	Two-tailed unpaired Student's t-test	Control vs. NEX-het-cKO	$t_{21} = 0.4969$, $P = 0.6244$ NS
Figure 4B	Control (n = 20 neurons from 4 mice) NEX-het-cKO (n = 20 neurons from 4 mice)	Two-way ANOVA	Control vs. NEX-het-cKO	$F_{1,38} = 28.93$, $P < 0.0001$ ***
		Post-hoc Bonferroni test	20 ms: Control vs. NEX-het-cKO	$t_{190} = 6.571$, $P < 0.0001$ ***
			50 ms: Control vs. NEX-het-cKO	$t_{190} = 3.846$, $P = 0.0008$ ***
			100 ms: Control vs. NEX-het-cKO	$t_{190} = 2.533$, $P = 0.0606$ NS
			200 ms: Control vs. NEX-het-cKO	$t_{190} = 2.022$, $P = 0.2228$ NS
			500 ms: Control vs. NEX-het-cKO	$t_{190} = 1.701$, $P = 0.4530$ NS
Figure 4E	Control (n = 8 neurons from 4 mice) NEX-het-cKO (n = 8 neurons from 5 mice)	Two-tailed unpaired Student's t-test with Welch's correction	Control vs. NEX-het-cKO	$t_{8.869} = 2.556$, $P = 0.0312$ *
Figure 4L	Control (n = 6 mice) NEX-het-cKO (n = 5 mice)	Two-way ANOVA	Control vs. NEX-het-cKO	$F_{1,9} = 9.923$, $P = 0.0117$ *
		Post-hoc Bonferroni test	5 pulses: Control vs. NEX-het-cKO	$t_{36} = 0.3696$, $P > 0.9999$ NS
			10 pulses: Control vs. NEX-het-cKO	$t_{36} = 1.37$, $P = 0.7166$ NS
			20 pulses: Control vs. NEX-het-cKO	$t_{36} = 3.518$, $P = 0.0048$ **
			50 pulses: Control vs. NEX-het-cKO	$t_{36} = 4.953$, $P < 0.0001$ ***
Figure 4M	Control (n = 6 mice) NEX-het-cKO (n = 5 mice)	Two-tailed unpaired Student's t-test	Control vs. NEX-het-cKO	$t_9 = 3.116$, $P = 0.0124$ *

Figure 5B	Control (n = 3 mice)<br>NEX-het-cKO (n = 4 mice)	Negative binomial distribution model of DESeq2	Supplementary Table 1	Supplementary Table 1
Figure 5D	Control (n = 3 mice)<br>NEX-het-cKO (n = 4 mice)	Two-tailed unpaired Student's t-test	*Plcb1*: Control vs. NEX-het-cKO	$t_5 = 2.400$, $P = 0.0616$ NS
			Plcb2: Control vs. NEX-het-cKO	$t_5 = 0.9254$, $P = 0.3972$ NS
			Plcb3: Control vs. NEX-het-cKO	$t_5 = 1.961$, $P = 0.1072$ NS
			Plcb4: Control vs. NEX-het-cKO	$t_5 = 2.743$, $P = 0.0407$ *
			Dagla: Control vs. NEX-het-cKO	$t_5 = 1.477$, $P = 0.1998$ NS
			Daglb: Control vs. NEX-het-cKO	$t_5 = 0.08450$, $P = 0.9359$ NS
			Napepld: Control vs. NEX-het-cKO	$t_5 = 2.912$, $P = 0.0333$ *
			Magl: Control vs. NEX-het-cKO	$t_5 = 2.796$, $P = 0.0382$ *
			Abhd6: Control vs. NEX-het-cKO	$t_5 = 0.7698$, $P = 0.4762$ NS
			Faah: Control vs. NEX-het-cKO	$t_5 = 4.277$, $P = 0.0079$ **
			Naaa: Control vs. NEX-het-cKO	$t_5 = 2.854$, $P = 0.0356$ *
			Cox2: Control vs. NEX-het-cKO	$t_5 = 1.589$, $P = 0.173$ NS
			Cnr1: Control vs. NEX-het-cKO	$t_5 = 1.041$, $P = 0.3454$ NS
Figure 6B	Control (n = 3 mice)<br>NEX-het-cKO (n = 3 mice)	Two-tailed unpaired Student's t-test	MAGL: Control vs. het-KO	$t_4 = 3.533$, $P = 0.0242$ *
	Control (n = 3 mice)<br>NEX-het-cKO (n = 3 mice)		FAAH: Control vs. het-KO	$t_4 = 0.3999$, $P = 0.7097$ NS
	Control (n = 3 mice)<br>NEX-het-cKO (n = 3 mice)		NAAA: Control vs. het-KO	$t_4 = 0.1143$, $P = 0.9145$ NS
	Control (n = 3 mice)<br>NEX-het-cKO (n = 3 mice)		COX2: Control vs. het-KO	$t_4 = 1.307$, $P = 0.2614$ NS
	Control (n = 6 mice)<br>NEX-het-cKO (n = 6 mice)		NAPE-PLD: Control vs. het-KO	$t_{10} = 1.204$, $P = 0.2565$ NS
	Control (n = 3 mice)<br>NEX-het-cKO (n = 3 mice)		PLCβ3: Control vs. het-KO	$t_4 = 0.5498$, $P = 0.6117$ NS
	Control (n = 7 mice)<br>NEX-het-cKO (n = 7 mice)		CB1R: Control vs. het-KO	$t_{12} = 1.631$, $P = 0.1289$ NS
	Control (n = 5 mice)<br>NEX-het-cKO (n = 5 mice)		PPP2R1A: Control vs. het-KO	$t_8 = 3.771$, $P = 0.0055$ **
Figure 6D	Control (n = 3)<br>OA (n = 3)	Two-tailed unpaired Student's t-test	MAGL: Control vs. OA	$t_4 = 4.705$, $P = 0.0093$ **
	Control (n = 3)<br>OA (n = 3)		FAAH: Control vs. OA	$t_4 = 2.085$, $P = 0.1055$ NS
	Control (n = 3)<br>OA (n = 3)		NAAA: Control vs. OA	$t_4 = 2.005$, $P = 0.1154$ NS

	Control (n = 6) OA (n = 6)		COX2: Control vs. OA	$t_{10} = 1.842$, $P = 0.0953$ NS
	Control (n = 3) OA (n = 3)		NAPE-PLD: Control vs. OA	$t_{4} = 1.150$, $P = 0.3141$ NS
	Control (n = 6) OA (n = 6)		PLCβ3: Control vs. OA	$t_{10} = 1.931$, $P = 0.0823$ NS
	Control (n = 6) OA (n = 6)		CB1R: Control vs. OA	$t_{10} = 0.0061$, $P = 0.9953$ NS
Figure 6E	Control (n = 6) OA (n = 6)	Two-tailed unpaired Student's t-test	*Magl*: Control vs. OA	$t_{10} = 4.742$, $P = 0.0008$ ***
	Control (n = 6) OA (n = 6)		*Faah*: Control vs. OA	$t_{10} = 2.048$, $P = 0.0678$ NS
Figure 6G	Control (n = 3) PP2Ac OE (n = 3)	Two-tailed unpaired Student's t-test	PP2Ac: Control vs. PP2Ac OE	$t_{4} = 8.641$, $P = 0.001$ ***
	Control (n = 3) PP2Ac OE (n = 3)		MAGL: Control vs. PP2Ac OE	$t_{4} = 3.390$, $P = 0.0275$ *
	Control (n = 3) PP2Ac OE (n = 3)		FAAH: Control vs. PP2Ac OE	$t_{4} = 1.881$, $P = 0.1332$ NS
Figure 7F	Control (n = 5) EPZ011989 (n = 5)	Two-way ANOVA	Control vs. EPZ011989	$F_{1,8} = 113.3$, $P < 0.0001$ ***
		Post hoc Bonferroni test	1 day: Control vs. EPZ011989	$t_{24} = 3.818$, $P = 0.0025$ **
			2 days: Control vs. EPZ011989	$t_{24} = 5.172$, $P < 0.0001$ ***
			3 days: Control vs. EPZ011989	$t_{24} = 13.55$, $P < 0.0001$ ***
Figure 7G	Control (n = 4) EPZ011989 (n = 4)	Two-tailed unpaired Student's t-test	Control vs. EPZ011989	$t_{6} = 8.975$, $P = 0.0001$ ***
Figure 7H	Control (n = 3 mice) Het-KO (n = 3 mice)	Two-tailed unpaired Student's t-test	Control vs. Het-KO	$t_{4} = 2.870$, $P = 0.0455$ *
Figure 7I	Control (n = 6) OA (n = 6)	Two-tailed unpaired Student's t-test	Control vs. OA	$t_{10} = 2.833$, $P = 0.0178$ *
Figure 7J	Control (n = 3) PP2Ac OE (n = 3)	Two-tailed unpaired Student's t-test	Control vs. PP2Ac OE	$t_{4} = 4.868$, $P = 0.0082$ **
Figure 7K	Control (n = 3 mice) Het-KO (n = 3 mice)	Two-way ANOVA	Control vs. Het-KO	$F_{1,4} = 11.30$, $P = 0.0283$ *
		Post hoc Bonferroni test	4 h: Control vs. Het-KO	$t_{16} = 5.181$, $P = 0.0004$ ***
			8 h: Control vs. Het-KO	$t_{16} = 1.54$, $P = 0.572$ NS
			12 h: Control vs. Het-KO	$t_{16} = 4.195$, $P = 0.0027$ **
Figure 7L	Control (n = 3 mice) OA (n = 3 mice)	Two-way ANOVA	Control vs. OA	$F_{1,4} = 51.90$, $P = 0.0020$ **
		Post hoc Bonferroni test	4 h: Control vs. OA	$t_{16} = 5.415$, $P = 0.0002$ ***
			8 h: Control vs. OA	$t_{16} = 4.227$, $P = 0.0026$ **
			12 h: Control vs. OA	$t_{16} = 11.45$, $P < 0.0001$ ***
Figure 8E	Control (n = 20 neurons from 4 mice) NEX-het-cKO (n = 23 neurons from 4 mice)	Two-tailed unpaired Student's t-test	Control vs. NEX-het-cKO	$t_{41} = 3.132$, $P = 0.0032$ **
Figure 8I	Control (n = 15 neurons from 10 mice) NEX-het-cKO (n = 12 neurons from 6 mice)	One-way ANOVA	ANOVA results	$F_{2,31} = 31.61$, $P < 0.0001$ ***
		Post hoc Bonferroni test	Control vs. NEX-het-cKO	$t_{31} = 4.413$, $P = 0.0002$ ***
			Control vs. Control + AM251	$t_{31} = 7.788$, $P < 0.0001$ ***
Figure 8J		Wilcoxon matched-pairs signed rank test	Control: ACSF vs. S-DHPG	$W = 112.0$, $P = 0.0004$ ***

	Control + AM251 (n = 7 neurons from 4 mice)	Two-tailed paired Student's t-test	NEX-het-cKO: ACSF vs. S-DHPG	$t_{11} = 2.222$, $P = 0.0482$ *
		Two-tailed unpaired Student's t-test	ACSF: Control vs. NEX-het-cKO	$t_{25} = 2.649$, $P = 0.0138$ *
		Two-tailed Mann-Whitney test	S-DHPG: Control vs. NEX-het-cKO	$U = 30$, $P = 0.0026$ **
Figure 9D	Control (n = 12 neurons from 7 mice) NEX-het-cKO (n = 12 neuron from 5 mice)	Two-tailed unpaired Student's t-test	Control vs. NEX-het-cKO	$t_{22} = 0.4167$, $P = 0.681$ NS
Figure 9E		Wilcoxon matched-pairs signed rank test	Control: ACSF vs. WIN	$W = 78$, $P = 0.0005$ ***
		Two-tailed paired Student's t-test	NEX-het-cKO: ACSF vs. WIN	$t_{11} = 4.320$, $P = 0.0012$ **
		Two-tailed unpaired Student's t-test	ACSF: Control vs. NEX-het-cKO	$t_{22} = 3.853$, $P = 0.0009$ ***
		Two-tailed Mann-Whitney test	WIN: Control vs. NEX-het-cKO	$U = 51$, $P = 0.2415$ NS
Figure 9F		Two-tailed unpaired Student's t-test with Welch's correction	Control vs. NEX-het-cKO	$t_{16.70} = 1.873$, $P = 0.0787$ NS
Figure 9J	Control (n = 13 neurons from 8 mice) NEX-het-cKO (n = 12 neurons from 6 mice)	Two-tailed Mann-Whitney test	Control vs. NEX-het-cKO	$U = 34$, $P = 0.016$ *
Figure 9K		Two-tailed paired Student's t-test	Control: ACSF vs. AM251	$t_{12} = 2.334$, $P = 0.0378$ *
		Wilcoxon matched-pairs signed rank test	NEX-het-cKO: ACSF vs. AM251	$W = 28$, $P = 0.3013$ NS
		Two-tailed Mann-Whitney test	ACSF: Control vs. NEX-het-cKO	$U = 16$, $P = 0.0003$ ***
		Two-tailed unpaired Student's t-test	AM251: Control vs. NEX-het-cKO	$t_{23} = 1.590$, $P = 0.1254$ NS
Figure 9L		Two-tailed unpaired Student's t-test	Control vs. NEX-het-cKO	$t_{23} = 2.289$, $P = 0.0316$ *
Figure 9S	Control (n = 7 mice) NEX-het-cKO (n = 6 mice)	Two-way ANOVA	Control vs. NEX-het-cKO	$F_{1, 11} = 9.610$, $P = 0.0101$ *
		Post-hoc Bonferroni test	25 µA: Control vs. NEX-het-cKO	$t_{44} = 0.01064$, $P > 0.9999$ NS
			50 µA: Control vs. NEX-het-cKO	$t_{44} = 0.5080$, $P > 0.9999$ NS
			100 µA: Control vs. NEX-het-cKO	$t_{44} = 3.146$, $P = 0.0119$ *
			150 µA: Control vs. NEX-het-cKO	$t_{44} = 5.387$, $P < 0.0001$ ***
Figure 9T		Two-tailed unpaired Student's t-test	Control vs. NEX-het-cKO	$t_{11} = 3.184$, $P = 0.0087$ **
Figure 10D	n = 5 mice	Two-tailed paired Student's t-test	ACSF vs. DO34	$t_4 = 7.359$; $P =0.0018$ **
Figure 10K	Control (n = 5 mice) NEX-het-cKO (n = 6 mice)	Two-way ANOVA	Control vs. NEX-het-cKO	$F_{1, 9} = 20.23$, $P = 0.0015$ **
		Post-hoc Bonferroni test	50 µA: Control vs. NEX-het-cKO	$t_{36} = 1.031$, $P > 0.9999$ NS
			100 µA: Control vs. NEX-het-cKO	$t_{36} = 3.178$, $P = 0.0122$ *
			150 µA: Control vs. NEX-het-cKO	$t_{36} = 4.506$, $P = 0.0003$ ***
			200 µA: Control vs. NEX-het-cKO	$t_{36} = 5.555$, $P < 0.0001$ ***
Figure 10L		Two-tailed unpaired Student's t-test	Control vs. NEX-het-cKO	$t_9 = 2.794$, $P = 0.0209$ *
Figure 10N	Control (n = 18 neurons from 3 mice) NEX-het-cKO (n = 21 neurons from 3 mice)	Two-way ANOVA	Control vs. NEX-het-cKO	$F_{1, 37} = 0.1797$, $P = 0.6741$ NS
		Post-hoc Bonferroni test	20 ms: Control vs. NEX-het-cKO	$t_{185} = 0.1121$, $P > 0.9999$ NS
			50 ms: Control vs. NEX-het-cKO	$t_{185} = 0.5333$, $P > 0.9999$ NS
			100 ms: Control vs. NEX-het-cKO	$t_{185} = 0.3575$, $P > 0.9999$ NS
			200 ms: Control vs. NEX-het-cKO	$t_{185} = 1.415$, $P = 0.7931$ NS
			500 ms: Control vs. NEX-het-cKO	$t_{185} = 0.07188$, $P > 0.9999$ NS
Figure 10P	Control (n = 17 neurons from 3	Two-way ANOVA	Control vs. NEX-het-cKO	$F_{1, 34} = 8.219$, $P = 0.0071$**
		Post-hoc	20 ms: Control vs. NEX-het-cKO	$t_{170} = 4.421$, $P < 0.0001$ ***

	mice) NEX-het-cKO (n = 19 neurons from 3 mice)	Bonferroni test	50 ms: Control vs. NEX-het-cKO	t_{170} = 3.68, P = 0.0016 **
			100 ms: Control vs. NEX-het-cKO	t_{170} = 1.643, P = 0.5107 NS
			200 ms: Control vs. NEX-het-cKO	t_{170} = 0.9413, P > 0.9999 NS
			500 ms: Control vs. NEX-het-cKO	t_{170} = 0.947, P > 0.9999 NS
Figure 10Q	Control (n = 13 mice) NEX-het-cKO (n = 10 mice) NEX-het-cKO + JZL18 4 (n = 10 mice)	Two-way ANOVA	**Number of primary errors** (Acquisition):<br>Control vs. NEX-het-cKO<br>NEX-het-cKO vs. NEX-het-cKO + JZL184	 <br>$F_{1,21}$ = 9.519, P = 0.0056 **<br>$F_{1,18}$ = 27.07, P < 0.0001 ***
			**Number of primary errors** (Reversal):<br>Control vs. NEX-het-cKO<br>NEX-het-cKO vs. NEX-het-cKO + JZL184	 <br>$F_{1,21}$ = 15.03, P = 0.0009 ***<br>$F_{1,18}$ = 14.74, P = 0.0012 **
		Post-hoc Bonferroni test	**Number of primary errors** (Acquisition):<br>day 1: Control vs. NEX-het-cKO<br>day 2: Control vs. NEX-het-cKO<br>day 3: Control vs. NEX-het-cKO<br>day 1: NEX-het-cKO vs. NEX-het-cKO + JZL184<br>day 2: NEX-het-cKO vs. NEX-het-cKO + JZL184<br>day 3: NEX-het-cKO vs. NEX-het-cKO + JZL184	 <br>t_{63} = 1.498, P = 0.4171 NS<br>t_{63} = 2.535, P = 0.0412 *<br>t_{63} = 1.854, P = 0.2053 NS<br>t_{54} = 3.502, P = 0.0028 **<br>t_{54} = 3.160, P = 0.0078 **<br>t_{54} = 2.690, P = 0.0284 *
			**Number of primary errors** (Reversal):<br>day 4: Control vs. NEX-het-cKO<br>day 5: Control vs. NEX-het-cKO<br>day 6: Control vs. NEX-het-cKO<br>day 4: NEX-het-cKO vs. NEX-het-cKO + JZL184<br>day 5: NEX-het-cKO vs. NEX-het-cKO + JZL184<br>day 6: NEX-het-cKO vs. NEX-het-cKO + JZL184	 <br>t_{63} = 1.705, P = 0.2793 NS<br>t_{63} = 2.576, P = 0.0371 *<br>t_{63} = 3.017, P = 0.0110 *<br>t_{54} = 2.220, P = 0.0919 NS<br>t_{54} = 2.540, P = 0.0419 *<br>t_{54} = 2.609, P = 0.0352 *
Figure 10R		Two-way ANOVA	**Primary latency** (Acquisition):<br>Control vs. NEX-het-cKO.<br>NEX-het-cKO vs. NEX-het-cKO + JZL184	 $F_{1,21}$ = 7.807, P = 0.0109 *<br>$F_{1,18}$ = 7.758, P = 0.0122 *
			**Primary latency** (Reversal):<br>Control vs. NEX-het-cKO<br>NEX-het-cKO vs. NEX-het-cKO + JZL184	 $F_{1,21}$ = 4.968, P = 0.0369 *<br>$F_{1,18}$ = 4.904, P = 0.0399 *
		Post-hoc Bonferroni test	**Primary latency** (Acquisition):<br>day 1: Control vs. NEX-het-cKO<br>day 2: Control vs. NEX-het-cKO<br>day 3: Control vs. NEX-het-cKO<br>day 1: NEX-het-cKO vs. NEX-het-cKO + JZL184<br>day 2: NEX-het-cKO vs. NEX-het-cKO + JZL184<br>day 3: NEX-het-cKO vs. NEX-het-cKO + JZL184	 t_{63} = 2.008, P = 0.1468 NS<br>t_{63} = 2.793, P = 0.0207 *<br>t_{63} = 2.243, P = 0.0852 NS<br>t_{54} = 2.371, P = 0.0640 NS<br>t_{54} = 2.804, P = 0.0210 *<br>t_{54} = 2.114, P = 0.1175 NS

			**Primary latency** (Reversal):<br>day 4: Control vs. NEX-het-cKO<br>day 5: Control vs. NEX-het-cKO<br>day 6: Control vs. NEX-het-cKO	 $t_{63} = 0.4126$, $P > 0.9999$ NS<br>$t_{63} = 2.321$, $P = 0.0705$ NS<br>$t_{63} = 2.670$, $P = 0.0289$ *
			day 4: NEX-het-cKO vs. NEX-het-cKO + JZL184<br>day 5: NEX-het-cKO vs. NEX-het-cKO + JZL184<br>day 6: NEX-het-cKO vs. NEX-het-cKO + JZL184	$t_{54} = 0.9071$, $P > 0.9999$ NS<br>$t_{54} = 2.250$, $P = 0.0856$ NS<br>$t_{54} = 2.466$, $P = 0.0507$ NS
Figure 10S		Two-way ANOVA	**Primary path** (Acquisition):<br>Control vs. NEX-het-cKO<br>NEX-het-cKO vs. NEX-het-cKO + JZL184	 $F_{1,21} = 6.908$, $P = 0.0157$ *<br>$F_{1,18} = 18.01$, $P = 0.0005$ ***
			**Primary path** (Reversal):<br>Control vs. NEX-het-cKO<br>NEX-het-cKO vs. NEX-het-cKO + JZL184	 $F_{1,21} = 12.42$, $P = 0.0020$ **<br>$F_{1,18} = 9.742$, $P = 0.0059$ **
		Post-hoc Bonferroni test	**Primary path** (Acquisition):<br>day 1: Control vs. NEX-het-cKO<br>day 2: Control vs. NEX-het-cKO<br>day 3: Control vs. NEX-het-cKO<br>day 1: NEX-het-cKO vs. NEX-het-cKO + JZL184<br>day 2: NEX-het-cKO vs. NEX-het-cKO + JZL184<br>day 3: NEX-het-cKO vs. NEX-het-cKO + JZL184	 $t_{63} = 1.170$, $P = 0.7395$ NS<br>$t_{63} = 1.793$, $P = 0.2332$ NS<br>$t_{63} = 2.211$, $P = 0.0921$ NS<br>$t_{54} = 3.131$, $P = 0.0084$ **<br>$t_{54} = 2.820$, $P = 0.0201$ *<br>$t_{54} = 2.677$, $P = 0.0295$ *
			**Primary path** (Reversal):<br>day 4: Control vs. NEX-het-cKO<br>day 5: Control vs. NEX-het-cKO<br>day 6: Control vs. NEX-het-cKO<br>day 4: NEX-het-cKO vs. NEX-het-cKO + JZL184<br>day 5: NEX-het-cKO vs. NEX-het-cKO + JZL184<br>day 6: NEX-het-cKO vs. NEX-het-cKO + JZL184	 $t_{63} = 0.8390$, $P > 0.9999$ NS<br>$t_{63} = 1.703$, $P = 0.2807$ NS<br>$t_{63} = 2.884$, $P = 0.0161$ *<br>$t_{54} = 0.8419$, $P > 0.9999$ NS<br>$t_{54} = 1.737$, $P = 0.2643$ NS<br>$t_{54} = 2.415$, $P = 0.0574$ NS
Figure 10T		Kruskal-Wallis test	ANOVA results (**Direct**):	$P = 0.0062$ **
		Post-hoc Dunn's test	Control vs. NEX-het-cKO<br>Control vs. NEX-het-cKO + JZL184<br>NEX-het-cKO vs. NEX-het-cKO + JZL184	$P = 0.0369$ *<br>$P > 0.9999$ NS<br>$P = 0.0080$ **
		One-way ANOVA	ANOVA results (**Serial**):	$F_{2,30} = 0.9735$, $P = 0.3894$ NS
		Post-hoc Bonferroni test	Control vs. NEX-het-cKO<br>Control vs. NEX-het-cKO + JZL184<br>NEX-het-cKO vs. NEX-het-cKO + JZL184	$t_{30} = 1.361$, $P = 0.5513$ NS<br>$t_{30} = 0.3133$, $P > 0.9999$ NS<br>$t_{30} = 0.9851$, $P = 0.9974$ NS
		One-way ANOVA	ANOVA results (**Mixed**):	$F_{2,30} = 2.876$, $P = 0.0720$ NS
		Post-hoc Bonferroni test	Control vs. NEX-het-cKO<br>Control vs. NEX-het-cKO + JZL184<br>NEX-het-cKO vs. NEX-het-cKO + JZL184	$t_{30} = 1.260$, $P = 0.6526$ NS<br>$t_{30} = 1.291$, $P = 0.6202$ NS<br>$t_{30} = 2.398$, $P = 0.0687$ NS
Figure S1A	n = 5 mice	One-way ANOVA	ANOVA results	$F_{6,28} = 11.41$, $P < 0.0001$ ***

		Post-hoc Bonferroni test	Brain vs. Spinal cord<br>Brain vs. Heart<br>Brain vs. Lung<br>Brain vs. Liver<br>Brain vs. Spleen<br>Brain vs. Kidney	$t_{28} = 2.769$, $P = 0.2073$ NS<br>$t_{28} = 4.763$, $P = 0.0011$ **<br>$t_{28} = 3.585$, $P = 0.0265$ *<br>$t_{28} = 6.848$, $P < 0.0001$ ***<br>$t_{28} = 5.729$, $P < 0.0001$ ***<br>$t_{28} = 6.336$, $P < 0.0001$ ***
Figure S1B	n = 5 mice	One-way ANOVA	ANOVA results	$F_{6,28} = 5.865$, $P = 0.0005$ ***
		Post-hoc Bonferroni test	Cortex vs. Thalamus<br>Hippocampus vs. Thalamus<br>Thalamus vs. Striatum<br>Others	$t_{28} = 4.372$, $P = 0.0032$ **<br>$t_{28} = 4.518$, $P = 0.0022$ **<br>$t_{28} = 3.732$, $P = 0.0180$ *<br>$P > 0.05$ NS
Figure S1C	n = 7 mice	One-way ANOVA	ANOVA results	$F_{7,48} = 9.354$, $P < 0.0001$ ***
		Post-hoc Bonferroni test	E14 vs. P30<br>E14 vs. P60<br>E18 vs. P30<br>E18 vs. P60<br>P0 vs. P30<br>P0 vs. P60<br>P7 vs. P30<br>P14 vs. P30<br>P21 vs. P30<br>Others	$t_{48} = 5.541$, $P < 0.0001$***<br>$t_{48} = 3.331$, $P = 0.0467$ *<br>$t_{48} = 6.203$, $P < 0.0001$***<br>$t_{48} = 3.993$, $P = 0.0062$ **<br>$t_{48} = 6.235$, $P < 0.0001$ ***<br>$t_{48} = 4.025$, $P = 0.0056$ **<br>$t_{48} = 4.829$, $P = 0.0004$ ***<br>$t_{48} = 4.493$, $P = 0.0012$ **<br>$t_{48} = 3.319$, $P = 0.0484$ *<br>$P > 0.05$ NS
Figure S2B	Control (n = 8 mice)<br>NEX-het-KO (n = 6 mice)	Two-tailed unpaired Student's t-test	Control vs. NEX-het-KO	$t_{12} = 1.005$, $P = 0.3347$ NS
Figure S2C		Two-tailed Mann-Whitney test	Control vs. NEX-het-KO	$U = 18$, $P = 0.4725$ NS
Figure S2D		Two-tailed Mann-Whitney test	Control vs. NEX-het-KO	$U = 16.50$, $P = 0.3939$ NS
Figure S3B	Control (n = 17 mice)<br>NEX-het-cKO (n = 17 mice)	Two-tailed unpaired Student's t-test	Total distance travelled: Control vs. NEX-het-cKO	$t_{32} = 0.9330$, $P = 0.3578$ NS
Figure S3C		Two-tailed unpaired Student's t-test	Number of visits to center: Control vs. NEX-het-cKO	$t_{32} = 1.403$, $P = 0.1702$ NS
Figure S3D		Two-tailed Mann-Whitney test	Time in center: Control vs. NEX-het-cKO	$U = 109$, $P = 0.2313$ NS
Figure S3E		Two-tailed unpaired Student's t-test	Distance in center: Control vs. NEX-het-cKO	$t_{32} = 1.846$, $P = 0.0742$ NS
Figure S3G	Control (n = 13 mice)<br>NEX-het-cKO (n = 12 mice)	Two-tailed Mann-Whitney test	Percentage of time in open arm: Control vs. NEX-het-cKO	$U = 66$, $P = 0.5382$ NS
Figure 1H		Two-tailed unpaired Student's t-test	Percentage of visits to open arm: Control vs. NEX-het-cKO	$t_{23} = 2.115$, $P = 0.0455$ *
Figure S3I	Control (n = 11 mice)<br>NEX-het-KO (n = 9 mice)	Two-tailed unpaired Student's t-test	Total grooming time: Control vs. NEX-het-KO	$t_{18} = 2.352$, $P = 0.0303$ *
Figure S3J		Two-tailed Mann-Whitney test	Number of grooming: Control vs. NEX-het-KO	$U = 33.5$, $P = 0.2361$ NS
Figure S3L	Control (n = 13 mice)<br>NEX-het-cKO (n = 11 mice)	Two-tailed unpaired Student's t-test	Stride length: Control vs. NEX-het-cKO	$t_{22} = 1.830$, $P = 0.0809$ NS
Figure S3M			Stride irregularity: Control vs. NEX-het-cKO	$t_{22} = 0.4032$, $P = 0.6907$ NS
Figure S3N			Front base: Control vs. NEX-het-cKO	$t_{22} = 1.372$, $P = 0.1838$ NS
Figure S3O			Hind base: Control vs. NEX-het-cKO	$t_{22} = 1.320$, $P = 0.2003$ NS
Figure S3P			Overlap: Control vs. NEX-het-cKO	$t_{22} = 0.2022$, $P = 0.8416$ NS

Figure S3Q			Overlap irregularity: Control vs. NEX-het-cKO	$t_{22} = 3.182$, $P = 0.0043$ **
Figure S3R	Control (n = 11 mice) NEX-het-KO (n = 9 mice)	Two-tailed unpaired Student's t-test	Control vs. NEX-het-KO	$t_{18} = 1.313$, $P = 0.2056$ NS
Figure S3S	Control (n = 18 mice) NEX-het-KO (n = 17 mice)	Two-tailed unpaired Student's t-test	Control vs. NEX-het-KO	$t_{33} = 1.066$, $P = 0.2941$ NS
Figure S3T	Control (n = 11 mice) NEX-het-KO (n = 13 mice)	Two-tailed unpaired Student's t-test	Control vs. NEX-het-KO	$t_{22} = 1.628$, $P = 0.1178$ NS
Figure S4B	Control (n = 12 mice) NEX-het-KO (n = 11 mice)	Two-tailed unpaired Student's t-test	Control: Empty vs. Stranger (S1) NEX-het-KO: Empty vs. Stranger (S1)	$t_{22} = 14.21$, $P < 0.0001$ *** $t_{20} = 10.89$, $P < 0.0001$ ***
Figure S4C			Social index: Control vs. NEX-het-KO	$t_{21} = 1.929$, $P = 0.0673$ NS
Figure S4E			Control: Familiar (S1) vs. Novel (S2) NEX-het-KO: Familiar (S1) vs. Novel (S2)	$t_{22} = 5.479$, $P < 0.0001$ *** $t_{20} = 4.958$, $P < 0.0001$ ***
Figure S4F			Discrimination index: Control vs. NEX-het-KO	$t_{21} = 0.09414$, $P = 0.9259$ NS
Figure S4H	Control (n = 12 mice) NEX-het-KO (n = 11 mice)	Two-tailed unpaired Student's t-test	Control: Familiar vs. Novel NEX-het-KO: Familiar vs. Novel	$t_{22} = 7.309$, $P < 0.0001$ *** $t_{20} = 2.47$, $P = 0.0226$ *
Figure S4I			Discrimination index: Control vs. NEX-het-KO	$t_{21} = 1.285$, $P = 0.2129$ NS
Figure S4K	Control (43/80 trials, n = 13 mice) NEX-het-KO (37/80 trials, n = 12 mice)	Chi-square tests	Control vs. NEX-het-KO	$\chi^2 = 0.2253$, $P = 0.635$ NS
Figure S5B	Control (n = 9 slices from 3 mice) NEX-het-KO (n = 9 slices from 3 mice)	Two-way ANOVA	Control vs. NEX-het-KO	$F_{1,16} = 7.389$, $P = 0.0152$ *
		Post-hoc Bonferroni test	Layer 1	$t_{48} = 2.745$, $P = 0.0255$ *
			layer 2/3	$t_{48} = 2.359$, $P = 0.0673$ NS
			layer 5	$t_{48} = 2.186$, $P = 0.1012$ NS
Figure S5C	Control (n = 9 slices from 3 mice) NEX-het-KO (n = 9 slices from 3 mice)	Two-way ANOVA	Control vs. NEX-het-KO	$F_{1,16} = 4.908$, $P = 0.0416$ *
		Post-hoc Bonferroni test	Layer 1	$t_{48} = 1.840$, $P = 0.2160$ NS
			layer 2/3	$t_{48} = 3.207$, $P = 0.0072$ **
			layer 5	$t_{48} = 0.5755$, $P > 0.9999$ NS
Figure S5D	Control (n = 9 slices from 3 mice) NEX-het-KO (n = 9 slices from 3 mice)	Two-way ANOVA	Control vs. NEX-het-KO	$F_{1,16} = 3.304$, $P = 0.0879$ NS
		Post-hoc Bonferroni test	Layer 1	$t_{48} = 1.934$, $P = 0.1771$ NS
			layer 2/3	$t_{48} = 1.204$, $P = 0.7034$ NS
			layer 5	$t_{48} = 1.947$, $P = 0.1722$ NS
Figure S8B	n = 3 mice	RM one-way ANOVA *Post-hoc* Bonferroni test	GluA1 (ANOVA): Total protein vs. Cytosolic protein Total protein vs. Synaptosome Cytosolic protein vs. Synaptosome	$F_{2,4} = 10.44$, $P = 0.0259$ * $t_4 = 2.207$, $P = 0.2757$ NS $t_4 = 2.361$, $P = 0.2328$ NS $t_4 = 4.568$, $P = 0.0308$ *
			GluA2 (ANOVA): Total protein vs. Synaptosome Total protein vs. Cytosolic protein Cytosolic protein vs. Synaptosome	$F_{2,4} = 75.89$, $P = 0.0007$ *** $t_4 = 4.642$, $P = 0.0292$ * $t_4 = 7.562$, $P = 0.0049$ ** $t_4 = 12.20$, $P = 0.0008$ ***

			Glu N1 (ANOVA):<br>Total protein vs. Synaptosome<br>Total protein vs. Cytosolic protein<br>Cytosolic protein vs. Synaptosome	$F_{2,4}$ = 67.50, P = 0.0008 ***<br>t_4 = 2.600, P = 0.1802 NS<br>t_4 = 8.507, P = 0.0031 **<br>t_4 = 11.11, P = 0.0011 **
			GluN2A (ANOVA):<br>Total protein vs. Synaptosome<br>Total protein vs. Cytosolic protein<br>Cytosolic protein vs. Synaptosome	$F_{2,4}$ = 50.05, P = 0.0015 **<br>t_4 = 3.443, P = 0.0787 NS<br>t_4 = 6.414, P = 0.0091 **<br>t_4 = 9.857, P = 0.0018 **
			GluN2B (ANOVA):<br>Total protein vs. Synaptosome<br>Total protein vs. Cytosolic protein<br>Cytosolic protein vs. Synaptosome	$F_{2,4}$ = 18.85, P = 0.0092 **<br>t_4 = 2.104, P = 0.3097 NS<br>t_4 = 3.944, P = 0.0507 NS<br>t_4 = 6.047, P = 0.0113 *
			PSD95 (ANOVA):<br>Total protein vs. Synaptosome<br>Total protein vs. Cytosolic protein<br>Cytosolic protein vs. Synaptosome	$F_{2,4}$ = 112.6, P = 0.0003 ***<br>t_4 = 3.578, P = 0.0696 NS<br>t_4 = 10.83, P = 0.0012 **<br>t_4 = 14.41, P = 0.0004 ***
Figure S8D	Control<br>(n = 6 mice)<br>NEX-het-KO<br>(n = 6 mice)	Two-tailed unpaired Student's t-test	GluA1: Control vs. NEX-het-KO	t_{10} = 0.2458, P = 0.8108 NS
	Control<br>(n = 6 mice)<br>NEX-het-KO<br>(n = 6 mice)		GluA2: Control vs. NEX-het-KO	t_{10} = 0.1539, P = 0.8807 NS
	Control<br>(n = 3 mice)<br>NEX-het-KO<br>(n = 3 mice)		Glu N1: Control vs. NEX-het-KO	t_4 = 0.1365, P = 0.898 NS
	Control<br>(n = 3 mice)<br>NEX-het-KO<br>(n = 3 mice)		GluN2A: Control vs. NEX-het-KO	t_4 = 0.6481, P = 0.5522 NS
	Control<br>(n = 3 mice)<br>NEX-het-KO<br>(n = 3 mice)		GluN2B: Control vs. NEX-het-KO	t_4 = 0.5712, P = 0.5984 NS
	Control<br>(n = 3 mice)<br>NEX-het-KO<br>(n = 3 mice)		PSD95: Control vs. NEX-het-KO	t_4 = 0.5310, P = 0.6235 NS
Figure S9B	Control<br>(n = 6 mice)	Two-tailed unpaired Student's t-test	Control vs. NEX-het-KO	t_9 = 0.8132, P = 0.4371 NS
Figure S9C	NEX-het-KO<br>(n = 5 mice)		Control vs. NEX-het-KO	t_9 = 0.9828, P = 0.3514 NS
Figure S10A	Control<br>(n = 3 mice)<br>NEX-het-cKO<br>(n = 4 mice)	Negative binomial distribution model of DESeq2	Supplementary Table 1	Supplementary Table 1
Figure S10C	Control<br>(n = 3 mice)<br>NEX-het-KO<br>(n = 4 mice)	Negative binomial distribution model of DESeq2	Control vs. NEX-het-KO	*FDR* < 0.05

Figure S10E	Upregulated genes (n = 1 160)<br>GO BP genes (n = 23 210)<br>GO CC genes (n = 23 436)<br>GO MF genes (n = 22 707)<br>KEGG genes (n = 8 893)	One-tailed Fisher's exact test	Supplementary Table 1	Supplementary Table 1
Figure S10F	Downregulated genes (n = 73)<br>GO BP genes (n = 23 210)<br>GO CC genes (n = 23 436)<br>GO MF genes (n = 22 707)<br>KEGG genes (n = 8 893)	One-tailed Fisher's exact test	Supplementary Table 1	Supplementary Table 1
Figure S12A	Supplementary Table 3	One-tailed Fisher's exact test	Supplementary Table 3	Supplementary Table 3
Figure S13D	Control (n = 10 neurons from 5 mice)<br>NEX-het-cKO (n = 10 neuron from 4 mice)	Two-tailed unpaired Student's t-test	Control vs. NEX-het-cKO	$t_{18} = 0.5426$, $P = 0.5941$ NS
Figure S13E		Two-tailed paired Student's t-test	Control: ACSF vs. WIN<br>NEX-het-cKO: ACSF vs. WIN	$t_9 = 2.907$, $P = 0.0174$ *<br>$t_9 = 2.336$, $P = 0.0443$ *
		Two-tailed unpaired Student's t-test	ACSF: Control vs. NEX-het-cKO<br>WIN: Control vs. NEX-het-cKO	$t_{18} = 0.1640$, $P = 0.8715$ NS<br>$t_{18} = 1.009$, $P = 0.3262$ NS
Figure S13F		Two-tailed Mann-Whitney test	Control vs. NEX-het-KO	$U = 40$, $P = 0.4813$ NS
Figure S13J	Control (n = 11 neurons from 5 mice)<br>NEX-het-cKO (n = 8 neuron from 4 mice)	Two-tailed unpaired Student's t-test with Welch's correction	Control vs. NEX-het-cKO	$t_{7.947} = 0.6228$, $P = 0.5509$ NS
Figure S13K		Wilcoxon matched-pairs signed rank test	Control: ACSF vs. S-DHPG	$W = -24$, $P = 0.3203$ NS
		Two-tailed paired Student's t-test	NEX-het-cKO: ACSF vs. S-DHPG	$t_7 = 0.6423$, $P = 0.5411$ NS
		Two-tailed unpaired Student's t-test	ACSF: Control vs. NEX-het-cKO	$t_{17} = 1.121$, $P = 0.2779$ NS
		Two-tailed Mann-Whitney test	S-DHPG: Control vs. NEX-het-cKO	$U = 44$, $P > 0.9999$ NS
Figure S13L		Two-tailed unpaired Student's t-test	Control vs. NEX-het-KO	$t_{17} = 0.9258$, $P = 0.3675$ NS
Figure S14G	n = 5 slices from 1 mouse	Two-tailed paired Student's t-test	ACSF vs. AM251	$t_4 = 11.59$, $P = 0.0003$ ***
Figure S15G	n = 5 slices from 1 mouse	Two-tailed paired Student's t-test	ACSF vs. AM251	$t_4 = 16.76$, $P < 0.0001$ ***
Figure S15H	2-AG (n = 5 slices from 1 mouse)<br>AEA (n = 6 slices from 1 mouse)	Two-tailed unpaired Student's t-test with Welch's correction	2-AG vs. AEA	$t_{4.232} = 4.941$, $P = 0.0067$ **

Figure S16F	AEA (n = 5 slices from 1 mouse) 2-AG (n = 6 slices from 1 mouse)	Two-tailed unpaired Student's t-test	2-AG vs. AEA	$t_9 = 6.938$, $P < 0.0001$ ***
Figure S16I	n = 5 slices from 1 mouse	Two-way ANOVA	Control vs. NEX-het-KO	$F_{1,8} = 2.870$, $P = 0.1287$ NS
		Post-hoc Bonferroni test	50 µA: Control vs. NEX-het-KO	$t_{5.47} = 0.2217$, $P > 0.9999$ NS
			100 µA: Control vs. NEX-het-KO	$t_{4.183} = 2.036$, $P = 0.4335$ NS
			150 µA: Control vs. NEX-het-KO	$t_{7.946} = 1.83$, $P = 0.4195$ NS
			200 µA: Control vs. NEX-het-KO	$t_{7.667} = 1.995$, $P = 0.3307$ NS
Figure S17C	Control (n =19 neurons from 3 mice) NEX-het-cKO (n = 15 neurons from 3 mice)	Two-way ANOVA	Control vs. NEX-het-cKO	$F_{1,32} = 39.90$, $P < 0.0001$ ***
		Post-hoc Bonferroni test	20 ms: Control vs. NEX-het-cKO	$t_{160} = 7.994$, $P < 0.0001$ ***
			50 ms: Control vs. NEX-het-cKO	$t_{160} = 7.373$, $P < 0.0001$ ***
			100 ms: Control vs. NEX-het-cKO	$t_{160} = 4.730$, $P < 0.0001$ ***
			200 ms: Control vs. NEX-het-cKO	$t_{160} = 3.161$, $P = 0.0094$ **
			500 ms: Control vs. NEX-het-cKO	$t_{160} = 1.869$, $P = 0.3176$ NS
Figure S18G	Control (n = 5 mice) NEX-het-KO (n = 5 mice)	Two-way ANOVA	Control vs. NEX-het-cKO	$F_{1,8} = 5.646$, $P = 0.0448$ *
		Post-hoc Bonferroni test	50 µA: Control vs. NEX-het-KO	$t_{32} = 1.193$, $P = 0.6693$ NS
			100 µA: Control vs. NEX-het-KO	$t_{32} = 2.167$, $P = 0.1428$ NS
			150 µA: Control vs. NEX-het-KO	$t_{32} = 2.783$, $P = 0.0354$ *
			200 µA: Control vs. NEX-het-KO	$t_{32} = 2.940$, $P = 0.0240$ *
Figure S18H		Two-tailed unpaired Student's t-test	Control vs. NEX-het-cKO	$t_8 = 2.342$, $P = 0.0473$ *
Figure S19E	Control (n = 5 mice) NEX-het-cKO (n = 5 mice)	Two-way ANOVA	Control vs. NEX-het-cKO	$F_{1,8} = 0.01265$, $P = 0.9132$ NS
		Post-hoc Bonferroni test	5 pulses: Control vs. NEX-het-cKO	$t_{32} = 0.1752$, $P > 0.9999$ NS
			10 pulses: Control vs. NEX-het-cKO	$t_{32} = 0.03752$, $P > 0.9999$ NS
			20 pulses: Control vs. NEX-het-cKO	$t_{32} = 0.4222$, $P > 0.9999$ NS
			50 pulses: Control vs. NEX-het-cKO	$t_{32} = 0.1270$, $P > 0.9999$ NS
Figure S19F	Control (n = 5 mice) NEX-het-cKO (n = 5 mice)	Two-tailed unpaired Student's t-test	Control vs. NEX-het-cKO	$t_8 = 0.2510$, $P = 0.8081$ NS

Supplemental References

1. Long JZ, Li W, Booker L, Burston JJ, Kinsey SG, Schlosburg JE, et al. Selective blockade of 2-arachidonoylglycerol hydrolysis produces cannabinoid behavioral effects. *Nat Chem Biol.* 2009;5(1):37-44.
2. Ding Z, Huang G, Wang T, Duan W, Li H, Wang Y, et al. Genetic Ablation of GIGYF1, Associated With Autism, Causes Behavioral and Neurodevelopmental Defects in Zebrafish and Mice. *Biological psychiatry.* 2023;94(10):769-79.
3. Love MI, Huber W, and Anders S. Moderated estimation of fold change and dispersion for RNA-seq data with DESeq2. *Genome Biology.* 2014;15(12).
4. Yu G, Wang L-G, Han Y, and He Q-Y. clusterProfiler: an R Package for Comparing Biological Themes Among Gene Clusters. *Omics-a Journal of Integrative Biology.* 2012;16(5):284-7.
5. Imrichova H, Hulselmans G, Atak ZK, Potier D, and Aerts S. i-cisTarget 2015 update: generalized cis-regulatory enrichment analysis in human, mouse and fly. *Nucleic Acids Research.* 2015;43(W1):W57-W64.
6. Oughtred R, Rust J, Chang C, Breitkreutz BJ, Stark C, Willems A, et al. The BioGRID database: A comprehensive biomedical resource of curated protein, genetic, and chemical interactions. *Protein Sci.* 2021;30(1):187-200.
7. Shannon P, Markiel A, Ozier O, Baliga NS, Wang JT, Ramage D, et al. Cytoscape: a software environment for integrated models of biomolecular interaction networks. *Genome Res.* 2003;13(11):2498-504.
8. Dunham I, Kundaje A, Aldred SF, Collins PJ, Davis C, Doyle F, et al. An integrated encyclopedia of DNA elements in the human genome. *Nature.* 2012;489(7414):57-74.
9. Kundu S, Ji F, Sunwoo H, Jain G, Lee JT, Sadreyev RI, et al. Polycomb Repressive Complex 1 Generates Discrete Compacted Domains that Change during Differentiation (vol 65, pg 432, 2017). *Molecular Cell.* 2018;71(1):191-.
10. Kloet SL, Makowski MM, Baymaz HI, van Voorthuijsen L, Karemaker ID, Santanach A, et al. The dynamic interactome and genomic targets of Polycomb complexes during stem-cell differentiation. *Nature Structural & Molecular Biology.* 2016;23(7):682-90.
11. Yao Z, van Velthoven CTJ, Thuc Nghi N, Goldy J, Sedeno-Cortes AE, Baftizadeh F, et al. A taxonomy of transcriptomic cell types across the isocortex and hippocampal formation. *Cell.* 2021;184(12):3222-3241.
12. Saunders A, Macosko EZ, Wysoker A, Goldman M, Krienen FM, de Rivera H, et al. Molecular Diversity and Specializations among the Cells of the Adult Mouse Brain. *Cell.* 2018;174(4):1015-1030.
