# Supplementary material for: Ppp2r1a haploinsufficiency increases excitatory synaptic transmission and decreases spatial learning by impairing endocannabinoid signaling
Source: J Clin Invest. 2025 Aug 21;135(17):e185602. doi: 10.1172/JCI185602 (PMC12404745; doi:10.1172/JCI185602)

Full unedited gel for Figure 6A-B

CB1R

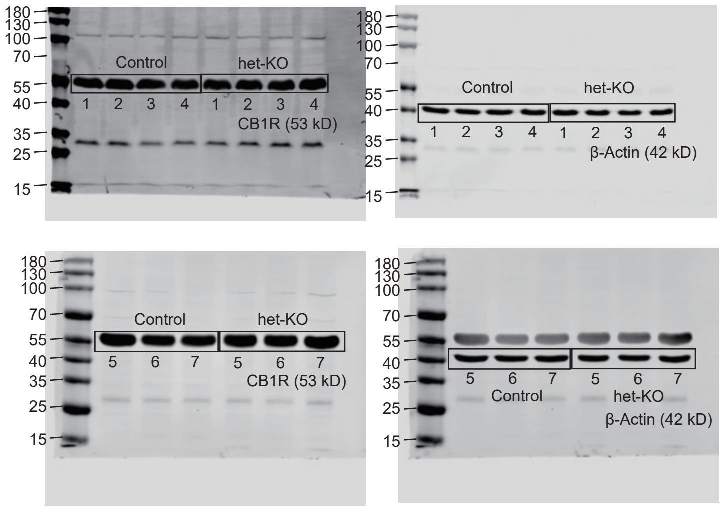

NAPD-PLD

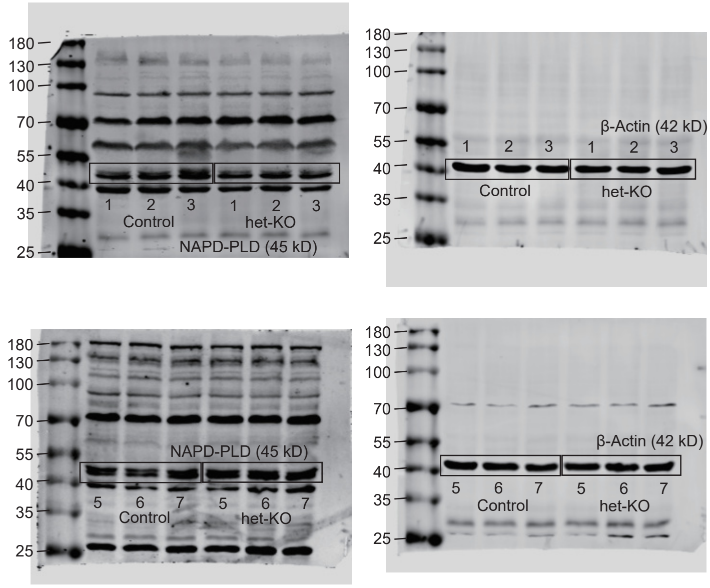

PPP2R1A

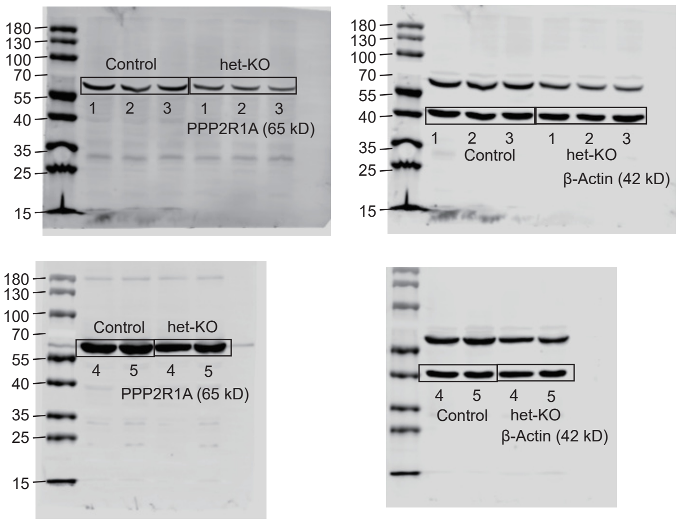

COX2

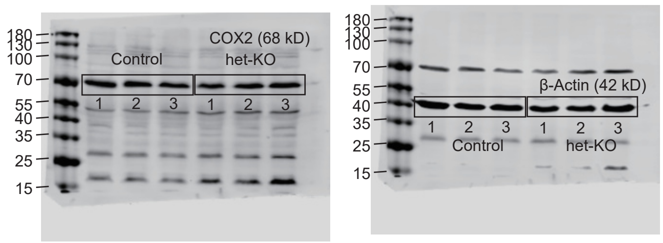

FAAH

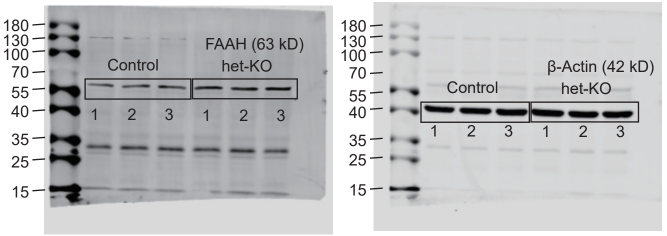

NAAA

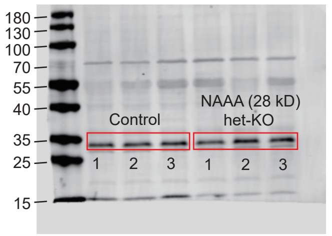

We first incubated the antibody against NAAA and imaged it (red box). Then, the antibodies against PLCβ3 and β-Actin were incubated and imaged (yellow box). NAAA and PLCβ3 shared the same loading control (β-Actin).

PLCβ3

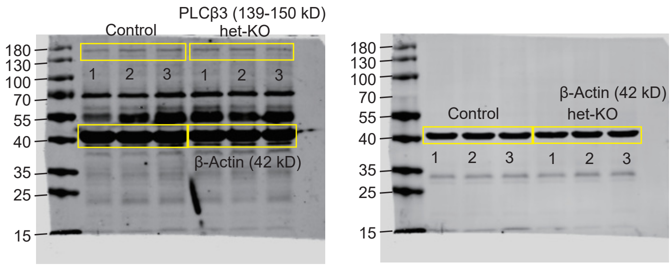

MAGL

The working concentration of the MAGL antibody is 1:600. Due to a limited amount of antibody, we selectively trimmed the top part of the membrane during the incubation period in our western blot experiment but imaged the entire blot in the final step. The signals for MAGL (red box) and tubulin (loading control, yellow box) remained unaffected.

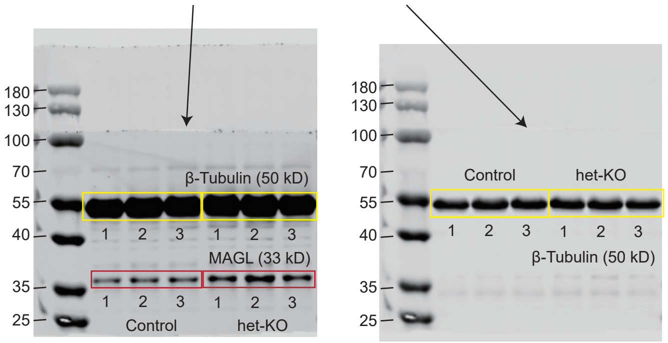

Full unedited gel for Figure 6C-D

CB1R

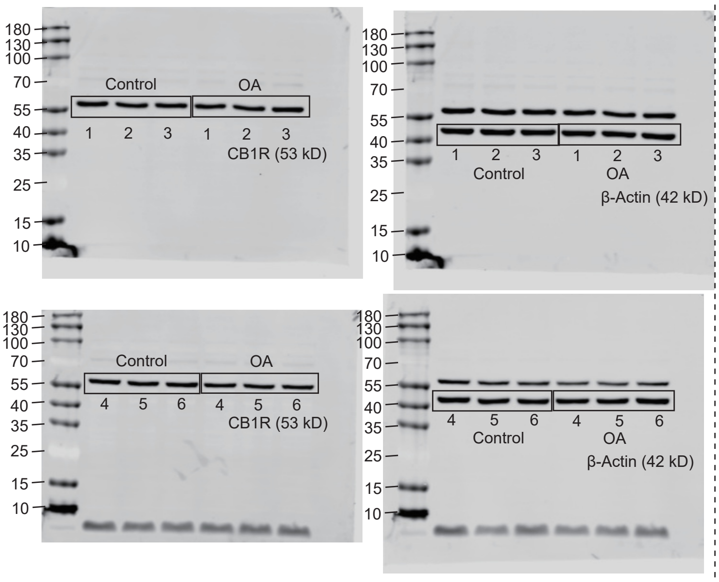

COX2

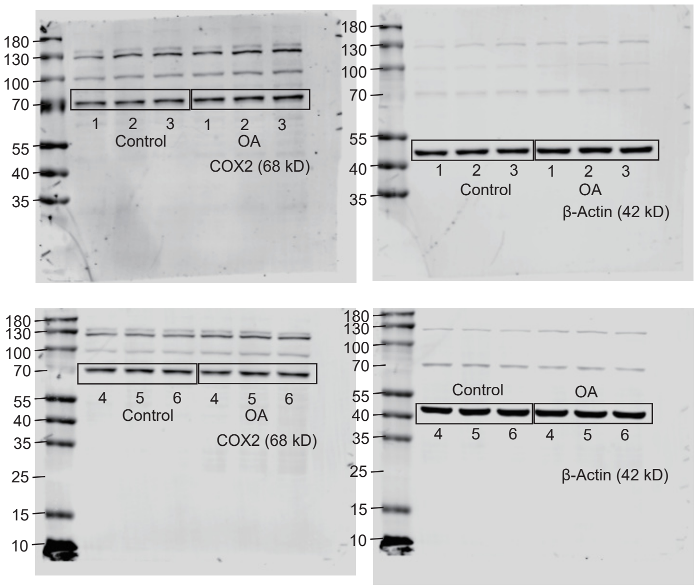

PLCβ3

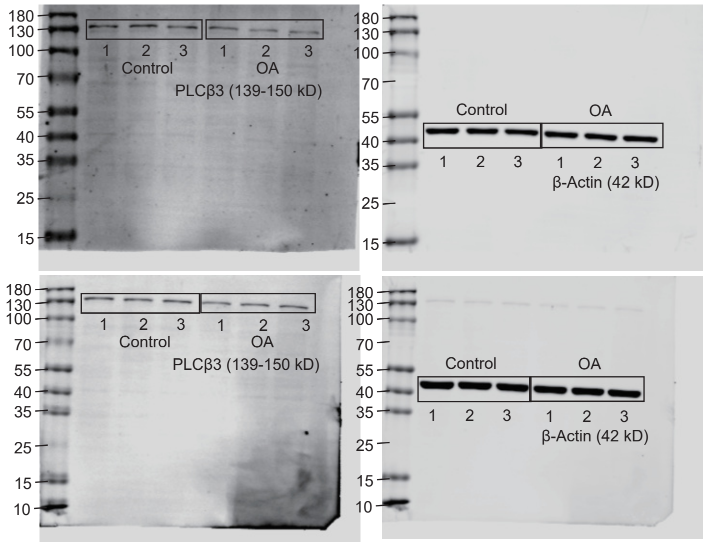

FAAH

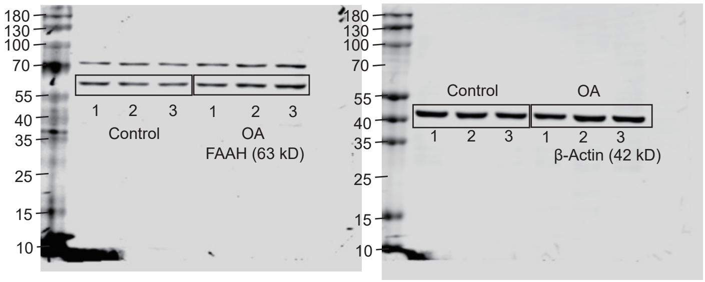

NAAA

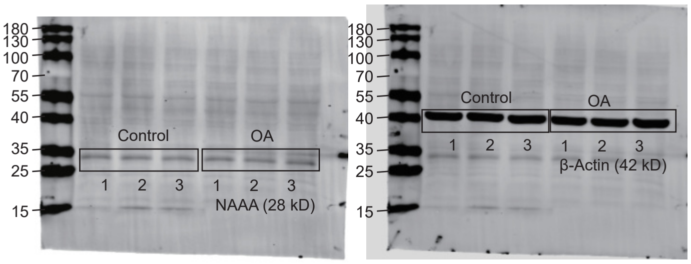

MAGL

The working concentration of the MAGL antibody is 1:600. Due to a limited amount of antibody, we selectively trimmed the top part of the membrane during the incubation period in our western blot experiment but imaged the entire blot in the final step. The signals for MAGL (red box) and tubulin (loading control, yellow box) remained unaffected.

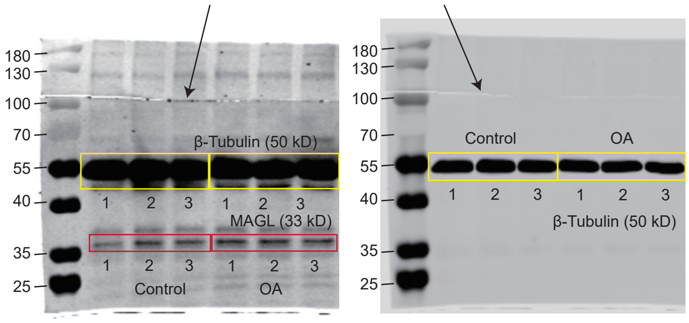

NAPD-PLD

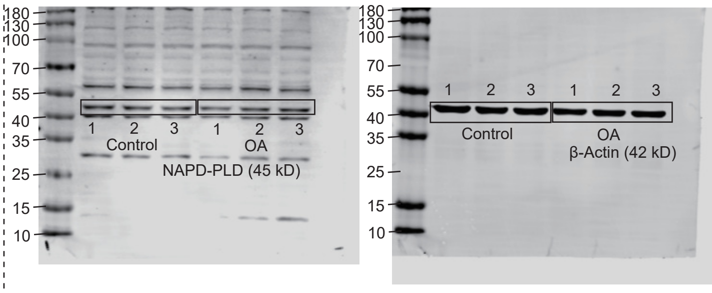

Full unedited gel for Figure 6F-G

PP2Ac (Figure 6F-G)

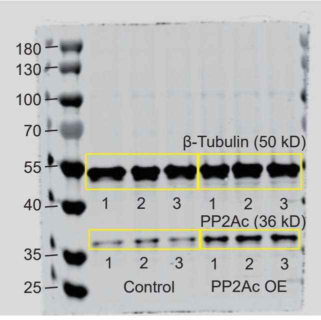

EZH2 (Figure 7J)

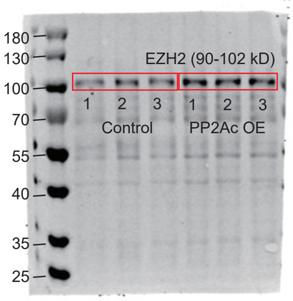

We first incubated the antibody against EZH2 and imaged it (red box). Then, the antibodies against PP2Ac and β-tubulin were incubated and imaged (yellow box). PP2Ac (Figure 6F-J) and EZH2 (Figure 7J) shared the same loading control (β-tubulin).

MAGL

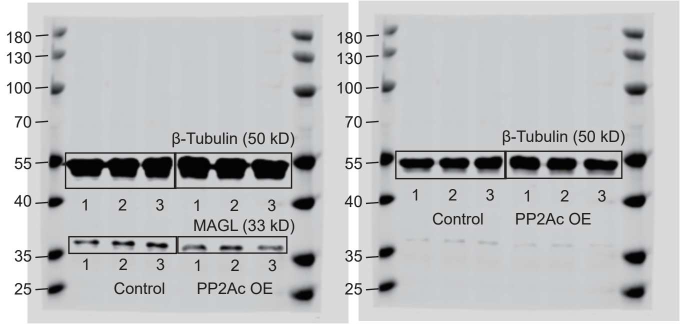

FAAH

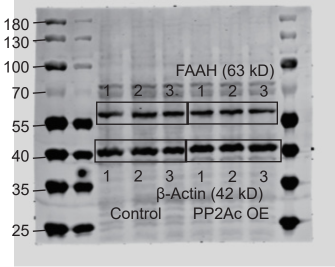

Full unedited gel for Figure 7

Figure 7G MAGL

The working concentration of the MAGL antibody is 1:600. Due to a limited amount of antibody, we selectively trimmed the top part of the membrane during the incubation period in our western blot experiment but imaged the entire blot in the final step. The signals for MAGL (red box) and tubulin (loading control, yellow box) remained unaffected.

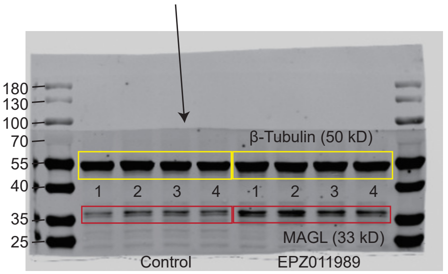

Figure 7H EZH2

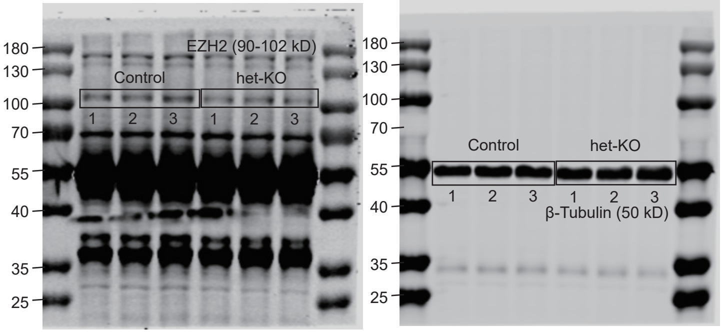

To visualize EZH2 in the left panel, higher exposure time and light intensity were applied during imaging.

Figure 7I EZH2

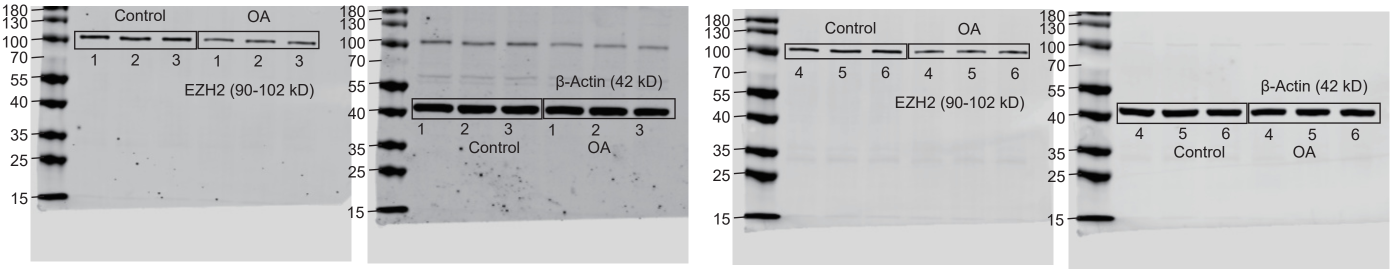

Figure 7K EZH2

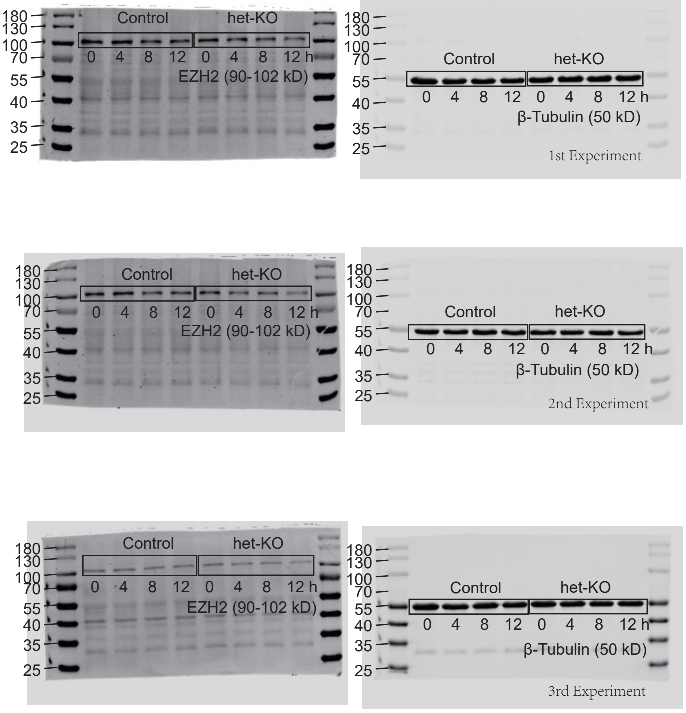

Figure 7L EZH2

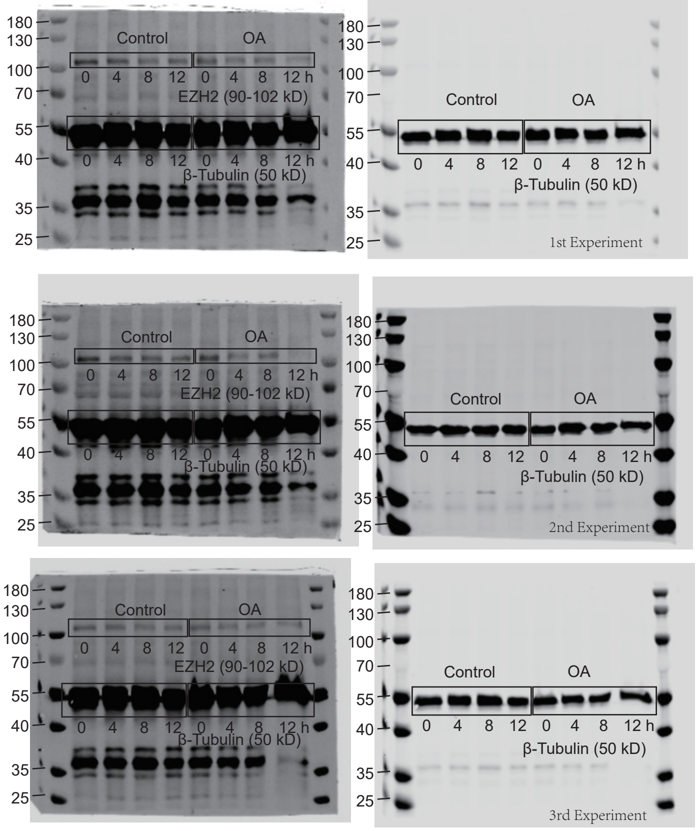

To visualize EZH2 in the left panel, higher exposure time and light intensity were applied during imaging.

Full unedited gel for Supplemental Figure 1

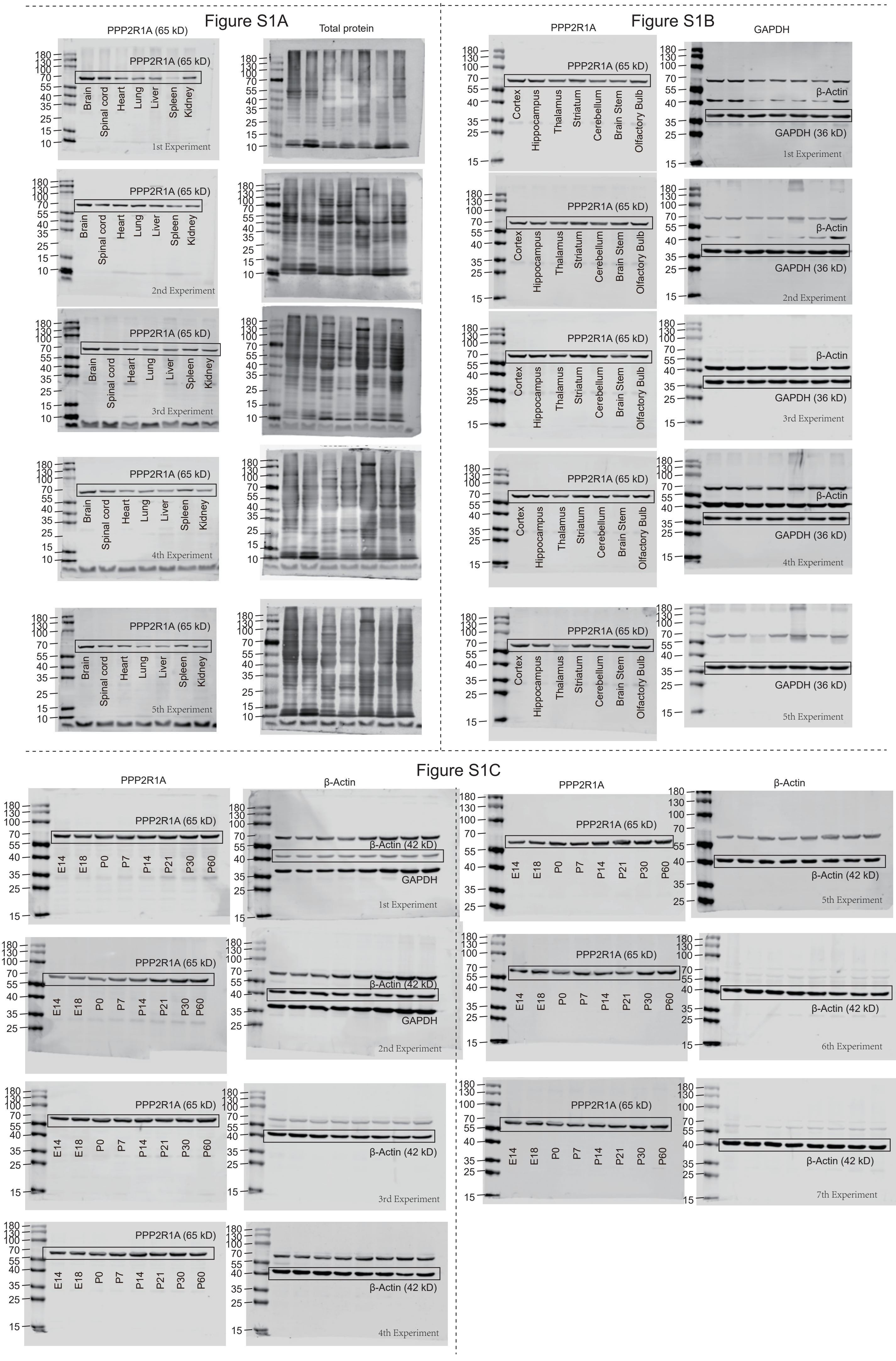

Full unedited gel for Supplemental Figure 8A-B

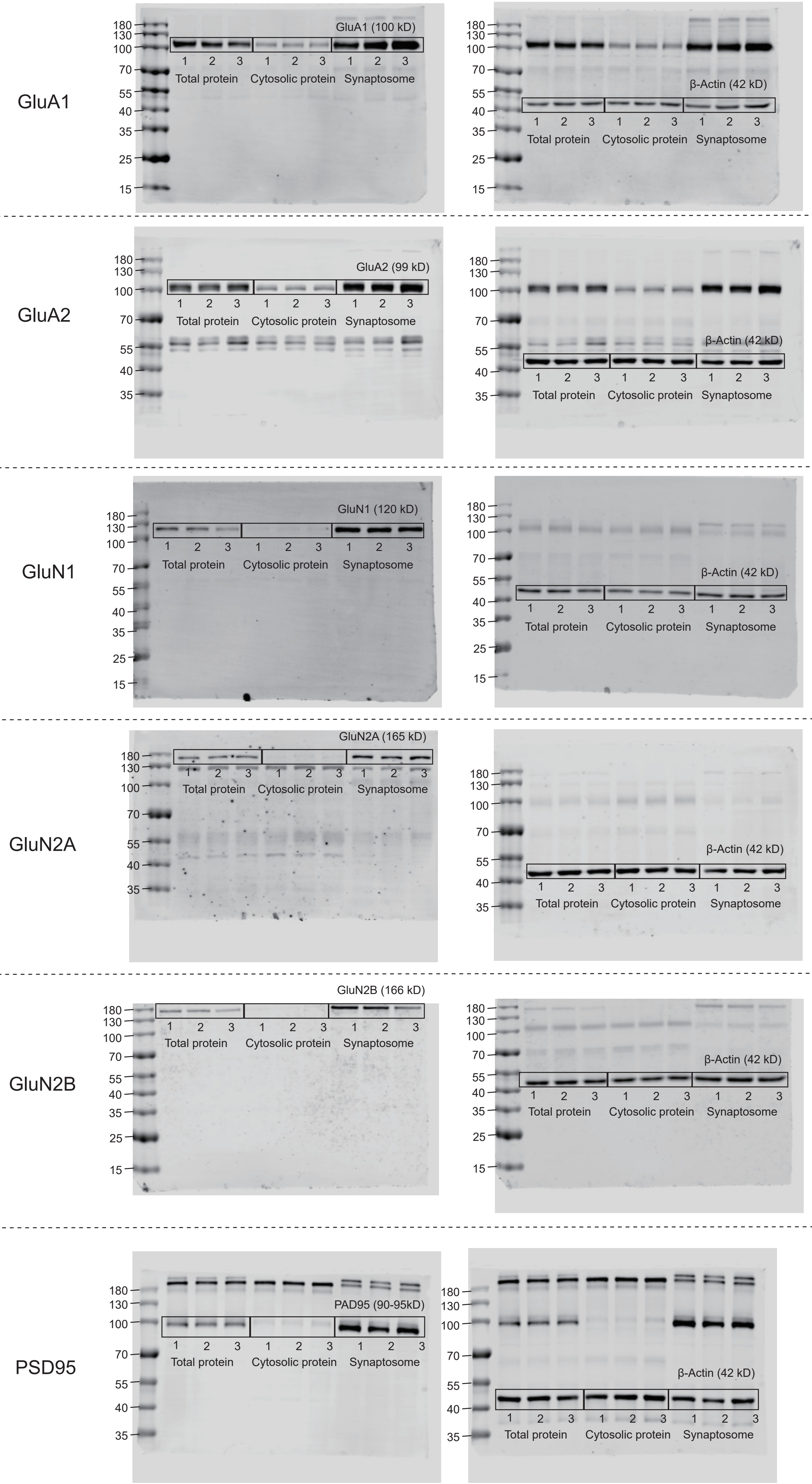

Full unedited gel for Supplemental Figure 8C-D

GluA1

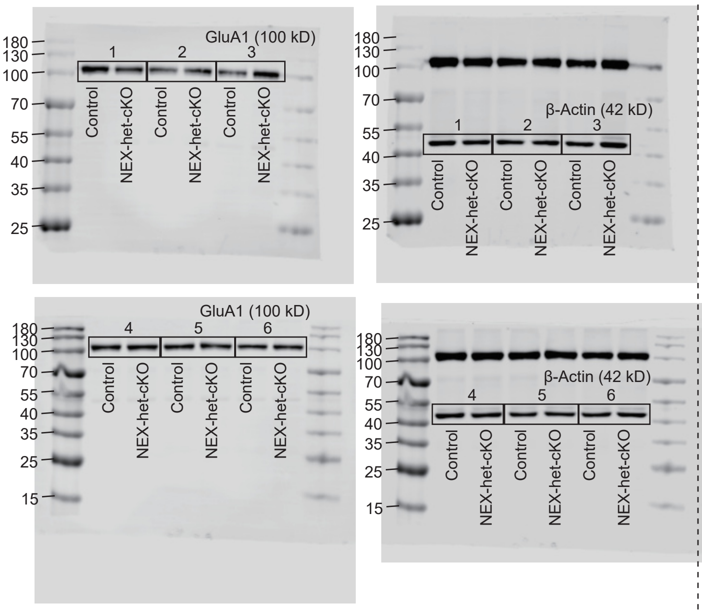

GluA2

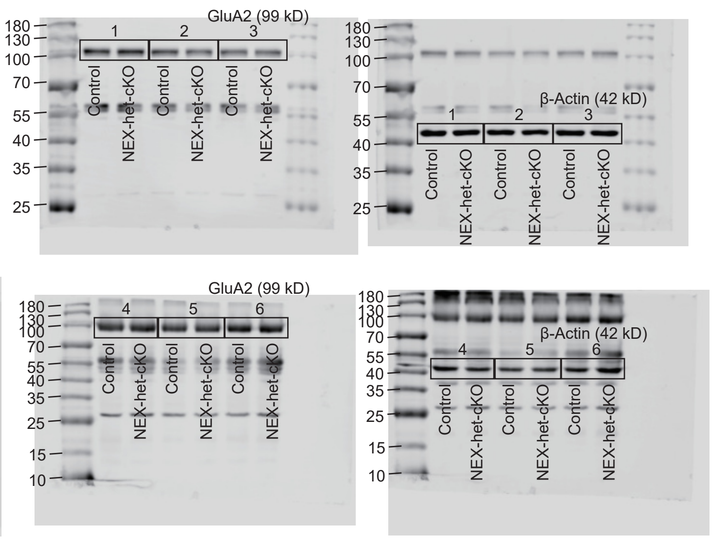

GluN1

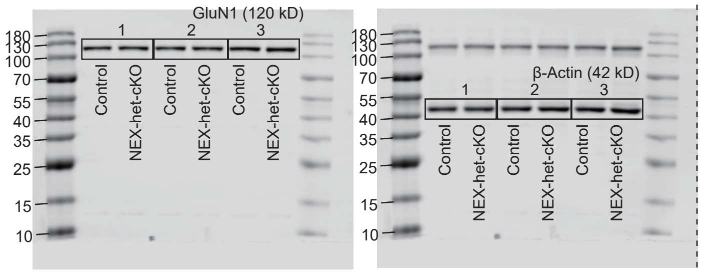

GluN2A

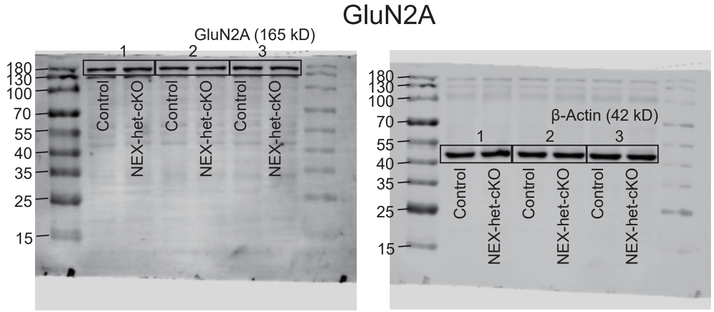

GluN2B

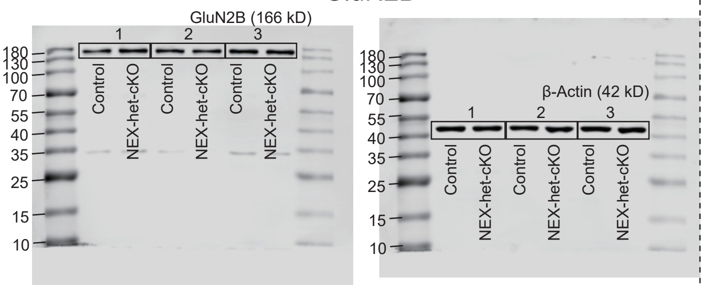

PSD95

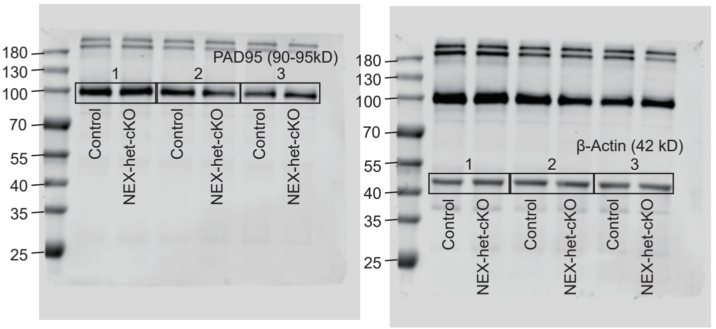

Supplement: Unedited blot and gel images [file jci-135-185602-s305.pdf]
